# Magnesium depletion score and erectile dysfunction: A cross-sectional and Mendelian randomization study

**DOI:** 10.1097/MD.0000000000049938

**Published:** 2026-07-24

**Authors:** Zhexin Zhang, Mo Yan, Siyuan Wu, Tongxi Li, Yong Wu, Yuezheng Li, Xuexue Hao, Chengyi Liu

**Affiliations:** a Department of Urology, Lu’an Hospital of Anhui Medical University, Lu’an People’s Hospital of Anhui Province, Lu’an, Anhui, China; b Department of Urology, Xiangya Hospital, Central South University, Changsha, Hunan, China; c Treatment Center of Kidney Disease, The First Affiliated Hospital of Henan University of Chinese Medicine, Zhengzhou, Henan, China; d Department of Orthopaedics, Heidelberg University Hospital, Heidelberg, Germany; e Guangdong Provincial Key Laboratory of Bone and Cartilage Regenerative Medicine, Nanfang Hospital, Southern Medical University, Guangzhou, Guangdong, China; f Department of Hepatobiliary Surgery, Union Hospital, Tongji Medical College, Huazhong University of Science and Technology, Wuhan, Hubei, China; g Departments of Neurosurgery, Xiangya Hospital, Central South University, Changsha, Hunan, China; h Department of Urology, Tianjin Medical University General Hospital, Tianjin, China.

**Keywords:** erectile dysfunction, magnesium depletion score, magnesium metabolism disorders, Mendelian randomization, NHANES

## Abstract

Erectile dysfunction (ED) is closely linked to vascular and metabolic disorders. Magnesium plays a crucial role in endothelial function, inflammation, and metabolism, but the relevance of chronic magnesium depletion to ED remains unclear. The magnesium depletion score (MDS) is a practical marker of long-term magnesium loss. This study examined the association between MDS and ED in United States adults and explored genetic evidence for magnesium metabolism using Mendelian randomization (MR). This cross-sectional study included 3692 men aged ≥ 20 years from National Health and Nutrition Examination Survey 2001 to 2004. MDS was calculated from renal function, diuretic and proton-pump inhibitor use, and alcohol consumption. Survey-weighted logistic regression, subgroup analysis, and restricted cubic splines were used to evaluate the association between MDS and ED, adjusting for demographic, lifestyle, and metabolic factors. Two-sample and multivariable MR analyses assessed genetic evidence for the relationship between magnesium metabolism disorders and ED, with adjustment for body mass index (BMI), type 2 diabetes, high-density lipoprotein cholesterol, and low-density lipoprotein cholesterol. Higher MDS was associated with increased odds of ED (fully adjusted odds ratio = 1.40; 95% confidence interval, 1.22–1.61; *P* = .002). Men with MDS ≥ 2 had more than twice the odds of ED compared with those with MDS < 2. Restricted cubic spline analysis demonstrated a nonlinear relationship, with the risk beginning to rise at approximately an MDS of 1 and increasing sharply when the MDS exceeded 3. Two-sample MR found no direct causal effect of magnesium metabolism disorders on ED. In multivariable MR, magnesium metabolism disorders showed a modest positive association with ED after adjustment for BMI, diabetes, and lipid traits (odds ratio = 1.01; 95% confidence interval, 1.00–1.02; *P* = .028). Genetically predicted higher BMI and diabetes were also associated with ED. Higher MDS was associated with increased odds of ED in United States adult men. MR findings provided limited but supportive evidence for a role of magnesium metabolism within a broader cardiometabolic context. Overall, MDS may serve as a potential risk marker for ED, but further prospective and interventional studies are needed to confirm its clinical relevance.

## 1. Introduction

Erectile dysfunction (ED) is one of the most prevalent male sexual disorders, affecting over 150 million men globally, with projections indicating that this number may exceed 320 million by 2025.^[1,2]^ This condition significantly impairs quality of life and psychosocial well-being, often serving as an early indicator of systemic vascular and metabolic diseases, including cardiovascular disease, diabetes, and metabolic syndrome.^[3]^ Consequently, identifying modifiable metabolic and vascular pathways that contribute to ED holds substantial clinical relevance.

Magnesium, an essential mineral, plays a crucial role in regulating vascular tone, glucose and lipid metabolism, and inflammatory responses that are central to erectile physiology.^[4]^ A deficiency in magnesium can lead to endothelial dysfunction, oxidative stress, and systemic inflammation,^[5]^ and has been associated with insulin resistance, metabolic syndrome, and diabetes.^[6]^ Nevertheless, the relationship between magnesium deficiency and ED remains unclear, as serum magnesium levels are tightly regulated and do not consistently indicate chronic deficiency.^[7]^

To address these limitations, the Magnesium Depletion Score (MDS) was developed as a composite index that incorporates renal function, the use of diuretics or proton-pump inhibitors (PPI), and alcohol intake.^[8,9]^ The MDS has been validated as a more accurate indicator of long-term magnesium deficiency and has been associated with inflammation, metabolic disorders, and cardiovascular mortality.^[10,11]^ However, its relevance to ED has not been previously evaluated.

Despite the increasing evidence linking magnesium deficiency to ED, observational studies remain susceptible to confounding factors and reverse causality, which limits the ability to make causal interpretations. Mendelian randomization (MR) addresses these challenges by utilizing genetic variants as instrumental variables to infer the causal relationship between an exposure and an outcome.^[12]^ These variants, typically single-nucleotide polymorphisms (SNPs) identified through genome-wide association studies (GWAS), are randomly allocated at conception and remain unaffected by environmental or behavioral influences. Consequently, MR simulates a natural randomized controlled trial, thereby minimizing residual confounding and eliminating reverse causation. The growing application of MR in epidemiology has enhanced causal inference for complex diseases, providing evidence that complements observational research and elucidates the biological mechanisms underlying disease risk.^[13]^

This study represents the first attempt to integrate National Health and Nutrition Examination Survey (NHANES)-based cross-sectional analysis with MR to investigate the relationship between magnesium depletion and ED. The objectives were to examine the association between MDS and ED in United States (U.S.) adult men and to evaluate the potential causal link between magnesium metabolism disorders and ED using 2-sample and multivariable MR (MVMR) analyses. These complementary approaches may help clarify whether magnesium-related metabolic disturbance is associated with ED and provide evidence for future prospective and interventional studies.

## 2. Methods

### 2.1. Study design and data source

This study adopted a cross-sectional design using data from the 2001 to 2004 cycles of NHANES, conducted by the National Center for Health Statistics under the U.S. Centers for Disease Control and Prevention. NHANES applies a complex, multistage probability sampling strategy to generate nationally representative estimates of the civilian, non-institutionalized U.S. population. The National Center for Health Statistics Institutional Review Board approved all protocols, and written informed consent was obtained from all participants.

**Figure 1.**
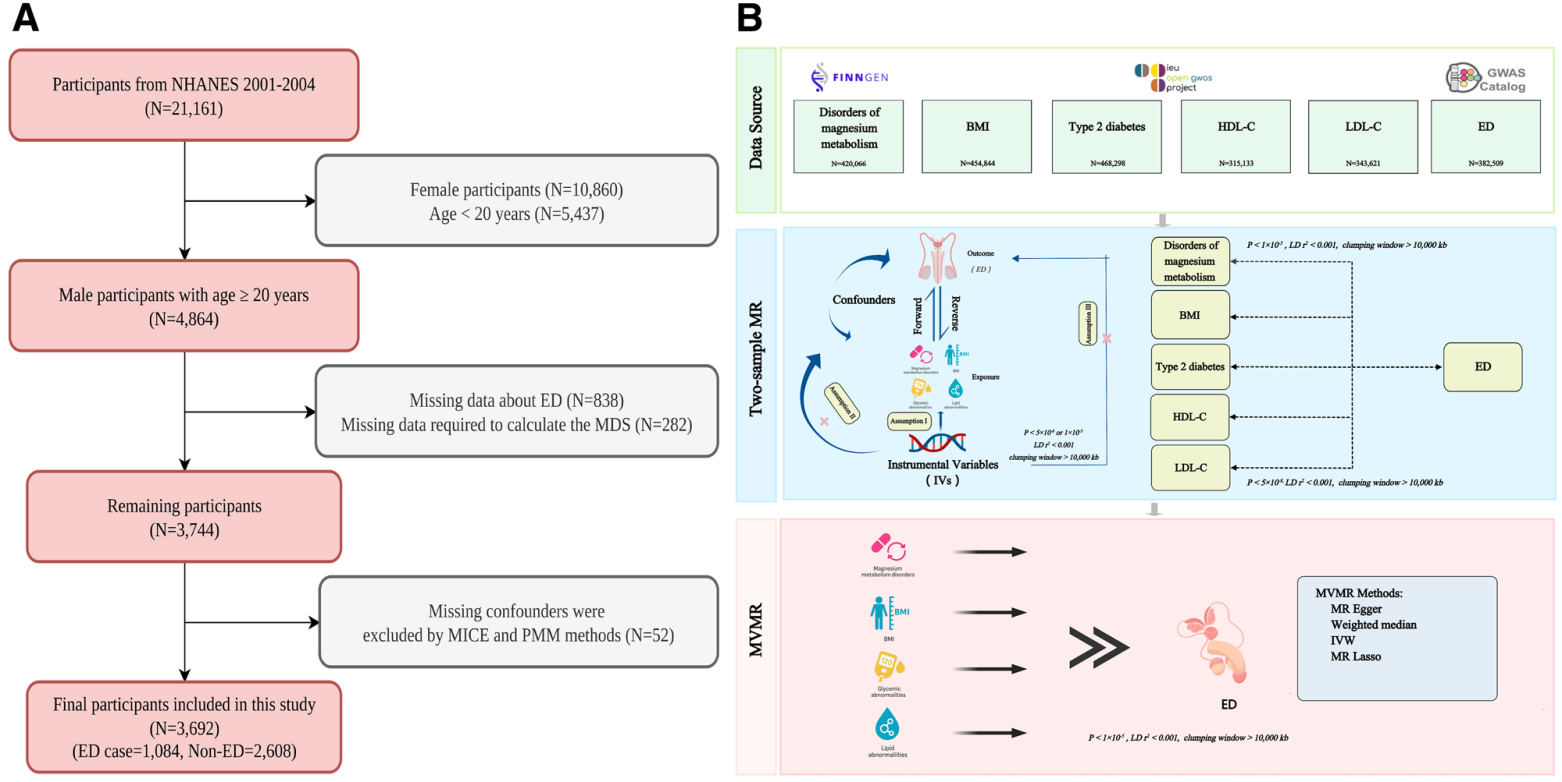
Study design and analytic framework. (A) Flowchart of participant selection in NHANES 2001 to 2004 (final analytic sample: 3692 men, including 1084 with ED and 2608 without ED); (B) 2-sample MR and MVMR framework evaluating the independent effects of magnesium metabolism, BMI, type 2 diabetes, HDL-C, and LDL-C on ED. BMI = body mass index, ED = erectile dysfunction, HDL-C = high-density lipoprotein cholesterol, IV = instrumental variable, IVW = inverse-variance weighted, LASSO = least shrinkage and selection operator, LD = liquid disequilibrium, LDL-C = low-density lipoprotein cholesterol, MDS = magnesium depletion score, MICE = multiple imputation by chained equations, MR = Mendelian randomization, MVMR = multivariable Mendelian randomization, N = number of participants, NHANES = National Health and Nutrition Examination Survey, PMM = predictive mean matching. Figure created with MedPeer (medpeer.cn).

Our analysis was restricted to men aged ≥ 20 years. Among 21,161 participants in NHANES 2001 to 2004, we excluded women (n = 10,860), those < 20 years (n = 5437), participants without ED information (n = 838), and those missing data required to calculate the MDS (n = 282). Missing covariate data were handled using multiple imputation by chained equations with 5 iterations and the predictive mean matching method to improve data robustness and minimize bias, following recent methodological recommendations.^[14]^ After excluding participants with incomplete covariate information (n = 52), a final analytic sample of 3692 men was included (Fig. 1A).

A 2-sample MR framework was applied to evaluate the causal relationship between magnesium metabolism and ED. Bidirectional analyses were performed where feasible to test for reverse causality. In addition, MVMR was conducted to examine whether magnesium metabolism exerts an independent effect on ED after accounting for body mass index (BMI), type 2 diabetes, high-density lipoprotein cholesterol (HDL-C), and low-density lipoprotein cholesterol (LDL-C). All procedures followed Strengthening the Reporting of Observational Studies in Epidemiology-MR guidelines.^[15]^ The overall study design is illustrated in Figure 1B.

### 2.2. Outcome variable in NHANES

The dependent variable was ED, measured in NHANES 2001 to 2004 by a single self-reported question based on that of the Massachusetts Male Aging Study.^[16]^ NHANES used a 1-item measure that has been found to be highly effective in research on ED in this data set, in contrast to the majority of clinical research, which has used the International Index of Erectile Function-5 questionnaire.^[17]^ Participants were questioned: “How often were you able to get and keep an erection that was sufficient for intercourse?” Available response categories were “always or almost always able,” “usually able,” “sometimes able,” and “never able.” In accord with previous analyses based on NHANES, we defined as normal erectile function men who responded that they were “always or almost always able” or “usually able,” and with ED those who responded that they were “sometimes able” or “never able.”^[16]^

### 2.3. Exposure variables in NHANES

The MDS was developed to assess chronic magnesium depletion risk, factoring in kidney function, diuretic use, PPI use, and alcohol consumption.^[10,18]^ First, calculation of kidney function was estimated with the estimated glomerular filtration rate (eGFR) as evaluated by serum creatinine and cystatin C and calculated by the following Chronic Kidney Disease Epidemiology Collaboration combined equation: eGFR = 135 × min (Scr/κ, 1)^α^ × max (Scr/κ, 1)^−0.544^ × min (Scys/0.8, 1)^−0.323^ × max (Scys/0.8, 1)^−0.778^ × 0.9961^Age^, where Scr is serum creatinine (mg/dL), Scys is cystatin C (mg/L), κ is 0.9, and α is −0.302 for men (all the participants in this analysis were male).^[19]^ One point was assigned for an eGFR between 60 to 90 mL/min/1.73 m^2^, and 2 points for an eGFR below 60 mL/min/1.73 m^2^. Additional points were given for current diuretic use, PPI use, and heavy drinking (> 2 drinks/day for men, with 1 drink equivalent to approximately 14 g of ethanol). The MDS ranged from 0 to 5, with higher scores indicating increased risk. Participants lacking data for any component were excluded.

### 2.4. Definition of covariates in NHANES

Covariates were selected based on established determinants of ED and encompassed demographic, lifestyle, dietary, and comorbidity factors. Demographic variables included age, categorized as < 40, 40 to 59, and ≥ 60 years; race (Mexican American, Non-Hispanic White, Non-Hispanic Black, Other Hispanic, and Other); education level (< 9th grade, 9–11th grade, high-school graduate, some college or Associate’s degree, and college graduate or above), and poverty-to-income ratio (PIR) classified as < 2, 2 to 3.99, and ≥ 4.^[20]^ Lifestyle factors included smoking; vigorous and moderate physical activity, defined as engaging in ≥ 10 minutes of exercise in the past 30 days according to intensity; and waist circumference (WC) dichotomized as < 102 cm or ≥ 102 cm.^[21]^ Dietary covariates comprised total energy intake (kcal/day) and dietary fiber intake (g/day). Comorbidities included hypertension, defined as systolic blood pressure ≥ 130 mm Hg, diastolic blood pressure ≥ 90 mm Hg, self-reported physician diagnosis, or antihypertensive medication use; diabetes, defined by self-report, insulin or oral hypoglycemic medication use, glycated hemoglobin (HbA1c) ≥ 6.5%, or fasting plasma glucose ≥ 126 mg/dL; and self-reported coronary heart disease (CHD) and congestive heart failure (CHF). Additional comorbidity-related covariates included HDL-C (categorized as ≥ 60, 40–59, and < 40 mg/dL), triglycerides (TG < 150, 150–199, and ≥ 200 mg/dL), serum C-reactive protein (CRP), and mental-health status, determined by any visit to a mental-health professional in the past year.^[22]^ Detailed covariate measurement protocols are available on the NHANES website ().

### 2.5. GWAS data sources

The outcome, ED, was obtained from the Million Veteran Program (N = 382,509 men of European ancestry, accession GCST90476161).^[23]^ Exposures included disorders of magnesium metabolism (FinnGen R12; N = 420,066; https://r12.finngen.fi/), BMI (United Kingdom [UK] Biobank; N = 454,884, accession ukb-b-2303),^[24]^ type 2 diabetes (UK Biobank; N = 468,298, accession ebi-a-GCST90029024),^[25]^ HDL-C (UK Biobank; N = 315,133, accession ebi-a-GCST90018956),^[26]^ and LDL-C (UK Biobank; N = 343,621, accession ebi-a-GCST90018961).^[26]^ All datasets consisted predominantly of European-ancestry participants. Only publicly available summary statistics were used; original studies had ethics approval and informed consent. All GWAS data are summarized in Table S1, Supplemental Digital Content 1.

### 2.6. Instrument selection

Instrumental variables were required to satisfy 3 assumptions: they must be associated with disorders of magnesium metabolism, BMI, type 2 diabetes, HDL-C, and LDL-C (relevance condition); they must be independent of any confounding factors (exclusion restriction condition); and they should influence the outcome solely through their effects on ED (exchangeability condition).^[27]^ SNPs reaching genome-wide significance (*P* < 5 × 10^−8^) were selected; when fewer than 3 were available, a relaxed threshold (*P* < 1 × 10^−5^) was applied.^[28]^ Linkage disequilibrium clumping was conducted at *R*^2^ < 0.001 within a 10,000 kb window using European reference data. SNPs with *F*-statistics < 10 or palindromic alleles at intermediate frequencies were excluded.^[29]^ All effect alleles were harmonized between the exposure and the outcome datasets for consistency, and Steiger filtering was then implemented to ensure that the alleles had the correct direction.^[30]^ These procedures were consistently applied to forward and reverse MR. After linkage disequilibrium clumping, harmonization, and exclusion of weak or ambiguous variants, the final numbers of independent SNPs included in the forward MR analyses were 5 for disorders of magnesium metabolism, 306 for BMI, 49 for type 2 diabetes, 201 for HDL-C, and 124 for LDL-C. In the reverse MR analyses, the numbers of ED-associated SNPs retained after harmonization varied across outcomes, including 18 for disorders of magnesium metabolism, 12 for BMI, 16 for type 2 diabetes, 17 for HDL-C, and 16 for LDL-C.

### 2.7. Statistical analysis

Analyses incorporated the NHANES sampling design by using strata, primary sampling units, and examination weights. Continuous variables were presented as weighted means with standard deviations and assessed via survey-weighted linear regression. Categorical variables were expressed as weighted percentages and evaluated using the Rao–Scott chi-square test. Associations between MDS and ED were examined with survey-weighted logistic regression, treating MDS both as a continuous and as a categorical variable (≥ 2 vs < 2). Univariable logistic regressions were first performed, followed by multivariable models with progressive adjustment for covariates. Subgroup analyses were conducted across predefined strata including age (< 40, 40–59, ≥ 60 years), PIR (< 2, 2–3.99, ≥ 4), race, education level, WC (< 102 vs ≥ 102 cm), fiber density, mental-health status, physical activity (moderate and vigorous activity), smoking, HDL-C (≥ 60, 40–59, < 40 mg/dL), TG (< 150, 150–199, ≥ 200 mg/dL), hypertension, and diabetes. Fiber density was calculated as dietary fiber intake (g) divided by total energy intake (kcal) and multiplied by 1000, yielding g/1000 kcal. Multiplicative interaction terms between MDS and each stratification variable were included to formally test for effect modification. Dose-response relationships were examined using restricted cubic spline (RCS) models with 3 to 6 knots placed at prespecified percentiles. The optimal specification was chosen by Akaike Information Criterion (AIC). Nonlinearity was tested using Wald chi-square tests. Sensitivity analyses included: additionally adjusting for CHF and CHD, and repeating RCS analyses with different knot specifications to confirm robustness.^[31]^ Results are presented as odds ratios (ORs) with 95% confidence intervals (CIs). A 2-sided *P* < .05 was considered statistically significant.

For the 2-sample MR analyses, causal estimates were primarily obtained using the inverse-variance weighted (IVW) method under a random-effects framework. Complementary methods included MR-Egger regression, weighted median, simple mode, and weighted mode.^[32]^ Between-instrument heterogeneity was assessed using Cochran *Q* statistic, and directional pleiotropy using the MR-Egger intercept.^[33]^ MR-pleiotropy residual sum and outliet was employed to identify pleiotropy and correct for outliers, and radial MR was applied to detect influential SNPs and reestimate causal effects after outlier removal.^[34]^ For MVMR, IVW, MR-Egger, weighted median, and least shrinkage and selection operator regression were applied to evaluate whether magnesium metabolism exerts an effect on ED independent of BMI, type 2 diabetes, and lipids.^[35]^ Sensitivity analyses included leave-one-out analyses, scatter plots, forest plots, funnel plots, and density plots to assess the influence of single variants, visualize SNP-level effects, and detect pleiotropy.^[28]^ Robustness was determined by consistency across estimators, before and after outlier correction, and in sensitivity analyses.

All statistical analyses were performed in R (version 4.5.1; R Foundation for Statistical Computing). NHANES analyses used the survey, tableone, rms, car, pROC, and ggplot2 packages; MR analyses used TwoSampleMR, ieugwasr/plinkbinr, MRPRESSO, RadialMR, MendelianRandomization, MVMR, and plotting with ggplot2 and ggsci.

## 3. Results

### 3.1. Baseline characteristics in NHANES

The characteristics of the study participants are presented in Table 1. A total of 3692 male participants from the 2001 to 2004 NHANES cycles were included, comprising 1084 individuals with ED and 2608 without ED. Participants with ED were markedly older than those without ED (mean age 61.3 ± 15.6 years vs 41.2 ± 13.4 years) and had greater WC (105.4 ± 15.3 cm vs 99.1 ± 14.2 cm). They also showed a substantially higher prevalence of hypertension (66.0% vs 36.9%) and diabetes (27.2% vs 6.4%).

**Table 1:** Baseline characteristics of the study population according to erectile dysfunction status.

Variable	Overall	ED		*P* value
	(n = 3692)	No (n = 2608)	Yes (n = 1084)	
Age, yrs, mean ± SD	45.17 ± 16.00	41.23 ± 13.42	61.28 ± 15.60	< .001
Education, n (weighted %)				< .001
< 9th grade	485 (5.8)	238 (3.9)	247 (13.4)	
9–11th grade	538 (10.8)	360 (9.8)	178 (14.9)	
High-school grad	912 (26.9)	689 (27.7)	223 (23.3)	
Some college or Associate’s degree	965 (29.7)	736 (31.0)	229 (24.3)	
College graduate or above	792 (26.8)	585 (27.5)	207 (24.1)	
Race, n (weighted %)				.252
Mexican American	754 (7.7)	536 (8.0)	218 (6.8)	
Non-Hispanic Black	660 (9.1)	501 (9.3)	159 (8.2)	
Non-Hispanic White	2036 (74.6)	1388 (74.1)	648 (76.8)	
Other Hispanic	130 (4.5)	93 (4.3)	37 (5.3)	
Other Race	112 (4.0)	90 (4.3)	22 (2.9)	
PIR, mean ± SD	3.22 ± 1.57	3.29 ± 1.57	2.95 ± 1.56	.003
Current smoking, n (weighted %)				< .001
No	1486 (42.8)	1165 (45.9)	321 (30.1)	
Yes	2206 (57.2)	1443 (54.1)	763 (69.9)	
Moderate activity, n (weighted %)				< .001
No	1873 (43.7)	1253 (41.8)	620 (51.5)	
Yes	1819 (56.3)	1355 (58.2)	464 (48.5)	
Vigorous activity, n (weighted %)				< .001
No	2446 (60.4)	1544 (55.6)	902 (80.2)	
Yes	1246 (39.6)	1064 (44.4)	182 (19.8)	
Hypertension, n (weighted %)				< .001
No	1875 (57.3)	1562 (63.1)	313 (34.0)	
Yes	1817 (42.7)	1046 (36.9)	771 (66.0)	
Diabetes, n (weighted %)				< .001
No	3159 (89.5)	2384 (93.6)	775 (72.8)	
Yes	533 (10.5)	224 (6.4)	309 (27.2)	
Mental-health visit, n (weighted %)				.047
No	3467 (93.0)	2458 (93.5)	1009 (90.9)	
Yes	225 (7.0)	150 (6.5)	75 (9.1)	
WC, cm, mean ± SD	100.32 ± 14.61	99.07 ± 14.17	105.43 ± 15.26	< .001
Total energy intake, kcal/d, mean ± SD	2665.99 ± 1123.23	2781.71 ± 1133.46	2192.61 ± 943.08	< .001
Dietary fiber intake, g/d, mean ± SD	17.84 ± 10.90	18.14 ± 11.01	16.61 ± 10.35	.001
HDL-C, mg/dL, mean ± SD	47.10 ± 12.78	47.17 ± 12.91	46.81 ± 12.22	.512
TG, mg/dL, mean ± SD	164.99 ± 174.56	163.17 ± 181.95	172.43 ± 140.22	.221
CRP, mg/dL, mean ± SD	0.34 ± 0.79	0.31 ± 0.76	0.45 ± 0.88	< .001
MDS, mean ± SD	0.44 ± 0.74	0.30 ± 0.59	0.99 ± 0.99	< .001

Values are presented as survey-weighted mean ± SD for continuous variables and unweighted n (survey-weighted %) for categorical variables.

CRP = C-reactive protein, ED = erectile dysfunction, HDL-C = high-density lipoprotein cholesterol, MDS = magnesium depletion score, n = number of participants, PIR = poverty-to-income ratio, SD = standard deviation, TG = triglycerides, WC = waist circumference.

*P* values were calculated using survey-weighted linear regression for continuous variables and the Rao–Scott *χ*^2^ test for categorical variables. A 2-sided *P* < .05 was considered statistically significant.

Socioeconomic and lifestyle differences were evident between groups. Men with ED had lower education levels and PIRs, were more likely to be current smokers (69.9% vs 54.1%), and were less likely to engage in moderate or vigorous physical activity. Dietary analysis indicated that men with ED had lower total energy intake (2192.6 ± 943.1 kcal/day vs 2781.7 ± 1133.5 kcal/day) and lower dietary fiber intake (16.6 ± 10.4 g/day vs 18.1 ± 11.0 g/day).

Serum CRP levels were significantly higher in participants with ED (0.45 ± 0.88 mg/dL vs 0.31 ± 0.76 mg/dL), whereas HDL-C and TG levels did not differ significantly between groups. Notably, the mean MDS was substantially higher among men with ED than those without ED (0.99 ± 0.99 vs 0.30 ± 0.59), indicating a greater degree of chronic magnesium deficiency in affected individuals.

### 3.2. Multivariable regression in NHANES

Table 2 presents the results of the weighted multivariable logistic regression analyses. When analyzed as a continuous variable, MDS exhibited a significant positive association with ED across all models. In the unadjusted model, each 1-unit increase in MDS was linked to nearly a threefold increase in the likelihood of ED (Model 1: OR = 2.96; 95% CI, 2.65–3.30). Following progressive adjustments for demographic, lifestyle, and clinical covariates, this association remained robust. In the fully adjusted model (Model 4), each 1-unit increase in MDS was associated with a 40% higher odds of ED (OR = 1.40; 95% CI, 1.22–1.61). When MDS was categorized, participants with an MDS of 2 or greater had more than twice the odds of ED compared with those with an MDS below 2 (Model 4: OR = 2.17; 95% CI, 1.69–2.78). The positive association persisted across all intermediate models, suggesting that the relationship between MDS and ED was not fully explained by the adjusted demographic, lifestyle, and clinical factors.

**Table 2:** Association between MDS and erectile dysfunction in multivariable logistic regression models.

Variable	Model 1		Model 2		Model 3		Model 4	
	OR (95% CI)	*P* value	OR (95% CI)	*P* value	OR (95% CI)	*P* value	OR (95% CI)	*P* value
MDS, per 1-point increase	2.96 (2.65–3.30)	< .001	1.52 (1.32–1.74)	< .001	1.44 (1.25–1.66)	< .001	1.40 (1.22–1.61)	.002
MDS < 2	Reference	-	Reference		Reference		Reference	-
MDS ≥ 2	8.46 (6.95–10.29)	< .001	2.50 (1.90–3.28)	< .001	2.27 (1.74–2.98)	< .001	2.17 (1.69–2.78)	< .001

Model 1: unadjusted. Model 2: adjusted for age, race, education, and PIR. Model 3: additionally adjusted for alcohol consumption, smoking status, and moderate and vigorous physical activity. Model 4: further adjusted for WC, hypertension, diabetes, dietary fiber intake, total energy intake, serum CRP, HDL-C, TG, and mental-health status. Estimates are derived from survey-weighted logistic regression models accounting for the NHANES complex sampling design.

CI = confidence interval, CRP = C-reactive protein, ED = erectile dysfunction, HDL = high-density lipoprotein cholesterol, MDS = magnesium depletion score, NHANES = National Health and Nutrition Examination Survey, OR = odds ratio, PIR = poverty-to-income ratio, TG = triglycerides, WC = waist circumference.

Two-sided *P* values are reported; *P* < .05 was considered statistically significant.

In Model 4, several covariates were also independently associated with ED (Table S2, Supplemental Digital Content 2). Older age showed a strong positive correlation with ED (OR = 1.08; 95% CI, 1.07–1.09). Lower educational attainment, particularly being a high-school graduate or below, was linked to a higher probability of ED. Socioeconomic disadvantage, reflected by a lower PIR, was also associated with greater ED prevalence.

Among modifiable factors, higher WC modestly increased ED risk (OR = 1.01; 95% CI, 1.00–1.02), and current smoking remained an independent predictor (OR = 1.30; 95% CI, 1.06–1.60). Diabetes was associated with more than double the odds of ED (OR = 2.19; 95% CI, 1.58–3.03). In contrast, hypertension, dietary fiber intake, and total energy intake were not significant predictors after adjustment. Serum CRP, HDL-C, and TG were likewise unrelated to ED in the multivariable model. Notably, mental-health consultation within the past year remained independently associated with ED (OR = 1.88; 95% CI, 1.29–2.75), underscoring the psychosocial contribution to erectile function.

### 3.3. Subgroup analysis in NHANES

**Figure 2.**
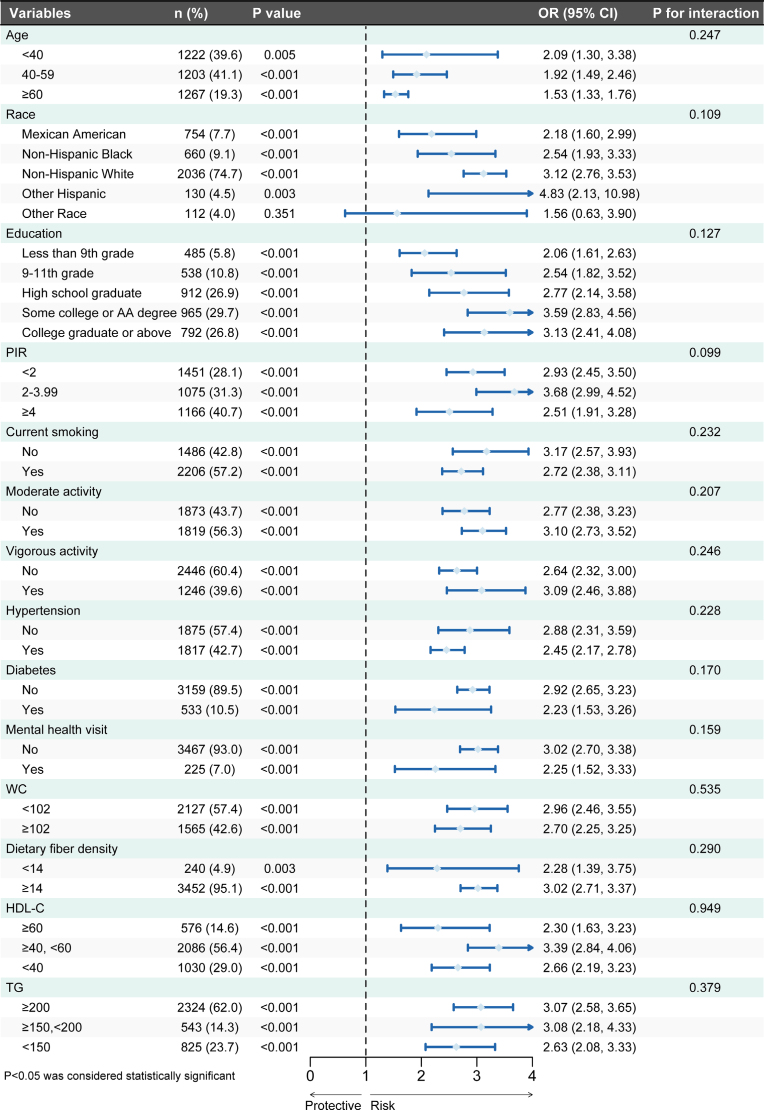
Subgroup analyses of the association between MDS and erectile dysfunction. Survey-weighted logistic regression models were used to evaluate the association between MDS and ED across predefined subgroups, including age, race, education, PIR, current smoking, physical activity, hypertension, diabetes, mental-health visit, WC, dietary fiber density, HDL-C, and TG. ORs with 95% CIs are shown for each subgroup, with interaction *P* values presented to test for effect modification. Models were progressively adjusted for demographic, lifestyle, and clinical covariates as described in the methods. No significant effect modification was observed. *P* < .05 was considered statistically significant. CI = confidence interval, ED = erectile dysfunction, HDL-C = high-density lipoprotein cholesterol, MDS = magnesium depletion score, PIR = poverty-to-income ratio, TG = triglycerides, WC = waist circumference.

Subgroup analyses confirmed the robust positive association between MDS and ED across various demographic, socioeconomic, lifestyle, and clinical subgroups. Consistent relationships were noted across age, race, education, and PIR categories. This association held regardless of smoking status, physical activity, WC, fiber density, mental-health status, and across strata defined by hypertension, diabetes, HDL-C, and TG. No significant effect modification was identified in any subgroup (Fig. 2).

### 3.4. Dose-response analysis in NHANES

**Figure 3.**
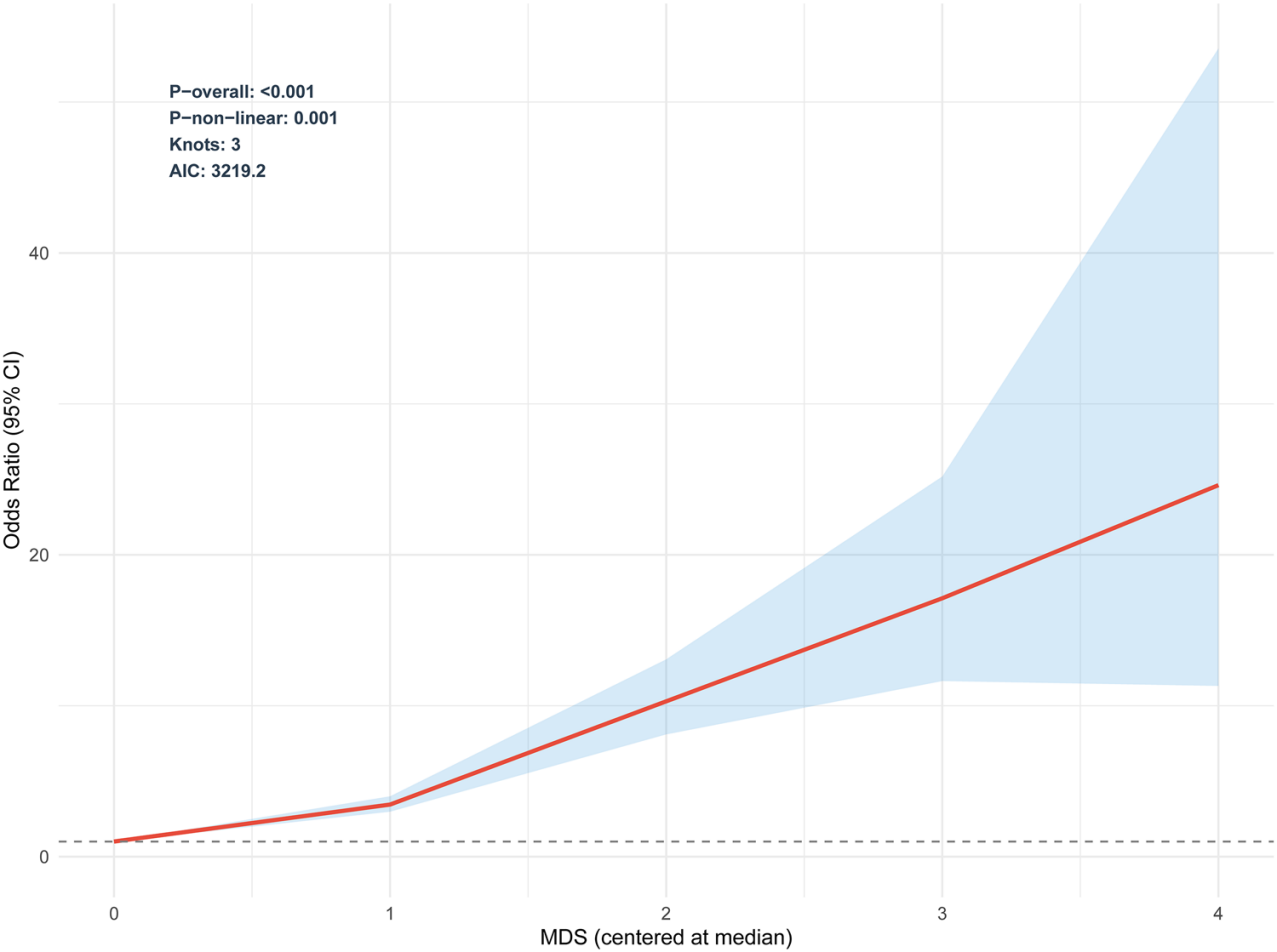
RCS analysis of the association between MDS and erectile dysfunction. The spline curve shows a significant nonlinear association (*P*_overall_ < .001, *P*_nonlinear_ = .001), with knots placed at 3 prespecified percentiles (AIC = 3219.2). ORs and 95% CIs are plotted relative to the median-centered MDS. The risk of ED increased progressively from approximately an MDS value of 1, with a steeper rise observed when MDS exceeded 3. AIC = Akaike information criterion, CI = confidence interval, ED = erectile dysfunction, MDS = magnesium depletion score, OR = odds ratio, RCS = restricted cubic spline.

RCS analysis demonstrated a significant nonlinear association between MDS and ED (*P*_overall_ < .001; *P*_nonlinear_ = .001). The best-fitting model was identified with 3 knots according to the lowest AIC. As shown in Figure 3, the odds of ED began to increase noticeably from around an MDS value of 1 and rose more sharply when MDS exceeded 3, with the fitted curve and corresponding 95% CIs supporting a robust nonlinear trend.

### 3.5. Sensitivity analyses in NHANES

The association between MDS and ED remained robust after additional adjustment for CHF and CHD (Tables S2 and S3, Supplemental Digital Content 2). Subgroup analyses incorporating CHF and CHD also yielded consistent associations (Table S4, Supplemental Digital Content 3). RCS sensitivity analyses with models using 3 to 6 knots demonstrated consistent nonlinear associations between MDS and ED, with the 3-knot model offering the best fit, evidenced by the lowest AIC (Fig. S1, Supplemental Digital Content 4).

### 3.6. Instrument selection and directionality

Instruments for the exposures related to ED (Table S5, Supplemental Digital Content 5) and for ED related to the exposures (Table S6, Supplemental Digital Content 6) were selected based on strict criteria. Steiger directionality tests consistently confirmed alignment of the causal direction in both forward and reverse MR analyses.

### 3.7. Forward and reverse 2-sample MR analyses

In forward MR, as detailed in Table S7, Supplemental Digital Content 7, genetically predicted disorders of magnesium metabolism were not associated with ED (IVW OR = 1.00, 95% CI: 0.98–1.01, *P* = .597). In contrast, higher genetically predicted BMI was associated with a greater risk of ED (IVW OR = 1.16, 95% CI: 1.11–1.20, *P* < .001). Genetic liability to type 2 diabetes showed a strong positive association with ED (IVW OR = 5.02, 95% CI: 3.25–7.76, *P* < .001). Lipid traits were directionally inverse: higher HDL-C was associated with lower ED risk (IVW OR = 0.94, 95% CI: 0.92–0.97, *P* < .001), and higher LDL-C was also associated with lower ED risk (IVW OR = 0.93, 95% CI: 0.94–0.96, *P* < .001).

In reverse MR, genetic predisposition to ED did not show statistically significant associations with magnesium metabolism disorders, BMI, type 2 diabetes, HDL-C, and LDL-C. Results from additional MR methods were directionally consistent and predominantly nonsignificant.

### 3.8. MVMR analyses

As shown in Table 3, in MVMR analyses adjusting for BMI, type 2 diabetes, HDL-C, and LDL-C, disorders of magnesium metabolism showed a consistent positive association with ED across all estimators. Using IVW, each genetically predicted unit increase in liability to magnesium metabolism disorders was associated with higher odds of ED (OR = 1.01, 95% CI: 1.00–1.02, *P* = .028).

**Table 3:** MVMR analysis of magnesium metabolism disorders and metabolic traits in relation to erectile dysfunction.

Exposure	Method	*P* value	OR (95% CI)
Disorders of magnesium metabolism	MR-Egger	.030	1.01 (1.00–1.02)
	Weighted median	.005	1.01 (1.00–1.02)
	IVW	.028	1.01 (1.00–1.02)
	MR-LASSO	.026	1.01 (1.00–1.01)
BMI	MR-Egger	.012	1.15 (1.03–1.28)
	Weighted median	.002	1.12 (1.04–1.19)
	IVW	< .001	1.13 (1.05–1.21)
	MR-LASSO	.001	1.08 (1.03–1.13)
Type 2 diabetes	MR-Egger	.001	3.16 (1.63–6.14)
	Weighted median	< .001	3.90 (2.17–7.02)
	IVW	.001	3.22 (1.66–6.22)
	MR-LASSO	< .001	4.65 (3.06–7.06)
HDL-C	MR-Egger	.276	0.97 (0.92–1.02)
	Weighted median	.225	0.97 (0.92–1.02)
	IVW	.304	0.97 (0.92–1.03)
	MR-LASSO	.060	0.97 (0.93–1.00)
LDL-C	MR-Egger	.012	0.93 (0.88–0.98)
	Weighted median	.022	0.94 (0.88–0.99)
	IVW	.013	0.93 (0.88–0.98)
	MR-LASSO	< .001	0.93 (0.90–0.97)

MVMR models were adjusted for BMI, type 2 diabetes, HDL-C, and LDL-C. ORs are expressed per genetically predicted unit increase in each exposure.

BMI = body mass index, CI = confidence interval, HDL-C = high-density lipoprotein cholesterol, IVW = inverse-variance weighted, LASSO = least shrinkage and selection operator, LDL-C = low-density lipoprotein cholesterol, MR = Mendelian randomization, MVMR = multivariable Mendelian randomization, OR = odds ratio.

Statistical significance was defined as a 2-sided *P* < .05.

Other exposures also demonstrated independent associations with ED. Higher genetically predicted BMI (IVW OR = 1.13, 95% CI: 1.05–1.21, *P* < .001) and type 2 diabetes (IVW OR = 3.22, 95% CI: 1.66–6.22, *P* = .001) were positively associated with ED, whereas LDL-C was inversely associated (IVW OR = 0.93, 95% CI 0.85–0.99, *P* = .013). No significant associations were observed for HDL-C.

### 3.9. Sensitivity analyses in MR

MVMR analyses were conducted using 4 complementary estimation methods: IVW, MR-Egger, weighted median, and MR-least shrinkage and selection operator, to ensure consistency of the causal estimates under different model assumptions (Table 3). The effect directions and magnitudes across these methods were highly concordant, supporting the robustness of the results.

No evidence of heterogeneity or horizontal pleiotropy was detected, as indicated by nonsignificant Cochran *Q* statistics, MR-Egger intercepts, and MR-pleiotropy residual sum and outliet global tests (Table S7, Supplemental Digital Content 7). Furthermore, graphical sensitivity diagnostics, including leave-one-out analyses, scatter plots, forest plots, funnel plots, and density plots, demonstrated stable effect estimates and the absence of influential outlier variants (Fig. S2, Supplemental Digital Content 8). Collectively, these findings indicate that the 2-sample MR and MVMR estimates were stable and not driven by pleiotropy or single-variant bias.

## 4. Discussion

In this integrated observational and genetic study, we identified a strong association between higher MDS and increased odds of ED in U.S. men. Participants with an MDS of ≥ 2 exhibited approximately double the odds of ED compared to those with lower scores. Furthermore, RCS analysis suggested a nonlinear increasing pattern between MDS and ED, with higher odds of ED observed at increasing MDS levels, particularly among participants with an MDS of 3 or higher. These findings indicate that MDS may reflect a magnesium depletion-related vascular-metabolic risk profile associated with ED. Although univariable 2-sample MR did not support a direct causal effect of magnesium metabolism disorders on ED, MVMR showed a modest association after accounting for BMI, type 2 diabetes, and lipid traits. These results suggest that magnesium metabolism may be involved in the cardiometabolic background of ED, rather than providing definitive evidence of a direct causal pathway.

Magnesium is essential for vascular tone, endothelial integrity, and metabolic regulation. It acts as a cofactor for endothelial nitric oxide synthase and supports nitric oxide-dependent vasodilation, thereby maintaining normal vascular relaxation.^[7,33]^ Evidence suggests that magnesium deficiency is associated with impaired nitric oxide signaling and oxidative inflammation, both of which are relevant to endothelial dysfunction and vasculogenic ED.^[4,36]^ Previous experimental and clinical studies have suggested that magnesium deficiency is associated with oxidative stress and arterial stiffness,^[36]^ and it counteracts the vasoconstrictor endothelin-1, thus preserving vascular function.^[4]^ Within this framework, the positive association between MDS and ED observed in our study is biologically plausible.

Magnesium plays a crucial role in metabolic regulation, particularly in insulin signaling and glucose transport.^[6]^ Low magnesium status has been associated with insulin resistance, type 2 diabetes, and dyslipidemia, which are also established correlates of ED.^[37,38]^ In the MVMR analysis, BMI and type 2 diabetes were independently associated with ED, while magnesium metabolism disorders showed a modest association after adjustment for these metabolic traits, suggesting a possible link between magnesium metabolism and the cardiometabolic profile of ED.

Inflammation may also be involved in the biological background underlying the association between magnesium depletion and ED. Although CRP was not independently associated with ED after comprehensive adjustment, a single inflammatory marker may not fully capture chronic low-grade inflammatory processes relevant to endothelial injury.^[39]^ Previous research suggests that oxidative stress and cytokine-driven inflammation may be involved in the pathophysiology of ED and magnesium deficiency.^[4]^ These findings underscore the multifaceted nature of endothelial dysfunction.

Our results support the potential utility of MDS as a practical indicator of long-term magnesium depletion risk. While serum magnesium levels are tightly regulated and may not accurately reflect true tissue stores, the MDS integrates factors such as renal function, the use of diuretics or PPI, and alcohol consumption, providing a more comprehensive assessment of chronic magnesium depletion.^[40,41]^ Prior research indicates that the MDS predicts inflammation, metabolic syndrome, and cardiovascular mortality more effectively than serum magnesium levels.^[18,42]^ Moreover, the nonlinear association observed between MDS and ED further suggests that higher MDS levels may capture a clinically relevant risk profile.

The findings from earlier studies align with this interpretation. Divergent findings between observational studies have been observed in serum concentrations of magnesium, which ranged from marginal to lower among young males with ED. However, serum concentrations of magnesium are not representative of chronic deficiency and are subject to confounding.^[43,44]^ Although our 2-sample MR also showed no relationship with ED, the MVMR model revealed a modest independent association between genetically proxied magnesium metabolism disorders and ED after adjusting for metabolic covariates. These findings suggest a possible relationship between magnesium metabolism and ED in a broader vascular-metabolic context, but they do not establish direct causality. Moreover, psychosocial factors remained independently associated with ED after multivariable adjustment, supporting evidence that emotional and behavioral determinants concurrently influence sexual function.^[45]^

Established cardiometabolic risk factors, including BMI, WC, and type 2 diabetes, have been consistently associated with ED in both observational and genetic models, aligning with previous studies.^[46,47]^ The lipid traits exhibited a more complex pattern. In 2-sample MR, genetically higher HDL-C appeared to be protective; however, this association disappeared in MVMR after adjusting for adiposity and glycemia, reinforcing the notion that HDL-C functions primarily as a biomarker rather than a causal factor.^[48]^ Conversely, LDL-C demonstrated an inverse association with ED in both MR and MVMR, contradicting its established atherogenic role. This paradox likely reflects collider or conditioning bias when adjusting for metabolic factors, residual pleiotropy of lipid-related variants, or hormonal mechanisms independent of atherosclerosis.^[49]^ Therefore, this inverse LDL-C signal should be interpreted as a potential artifact rather than genuine protection.

Clinically, these findings have significant implications. MDS may serve as a simple marker for identifying men with a higher likelihood of ED, particularly in the context of vascular and metabolic risk. An MDS score of 2 or higher indicates individuals with multiple depletion-related factors and a substantially elevated metabolic risk, which aligns with previous reports.^[18]^ Because several components of MDS are potentially modifiable, these findings may help generate hypotheses for future preventive or interventional studies. Appropriate evaluation of medication-related magnesium loss, alcohol consumption, and renal function may help identify individuals with a higher risk of magnesium depletion. These considerations may complement broader clinical assessment of ED-related cardiometabolic risk, including obesity, glycemic status, and cardiovascular health.

Future investigations should assess whether magnesium supplementation or dietary optimization can improve erectile and vascular outcomes. Current clinical guidelines for ED recommend cardiovascular risk modification and hormonal assessment, but they do not include magnesium monitoring.^[50]^ Randomized controlled trials are needed to determine whether magnesium repletion has any benefit for erectile or vascular outcomes. Given that ED often precedes overt cardiovascular disease, such interventions may offer dual benefits for both sexual and cardiometabolic health.

This study has notable strengths. Using nationally representative NHANES data with complex sampling weights and extensive covariate adjustment ensures generalizability. The MDS provides a pragmatic measure of magnesium deficiency derived from routinely available clinical information. Integration of observational analysis with 2-sample and MVMR minimized confounding and enhanced causal inference. Robust sensitivity analyses confirmed the stability of our results.

Nonetheless, several limitations warrant mention. The NHANES design is cross-sectional, precluding causal inference. In addition, ED was identified using a single self-reported question regarding the ability to achieve and maintain an erection sufficient for intercourse, rather than by clinical diagnosis or a validated multi-item instrument such as the International Index of Erectile Function-5. Although this approach has been widely used in NHANES-based ED studies, it may introduce recall bias, social desirability bias, and outcome misclassification, and it does not allow detailed assessment of ED severity or etiology. Residual confounding and measurement errors in MDS components are possible. The NHANES findings reflect U.S. men from 2001 to 2004 and may not generalize to contemporary or non-U.S. populations, while the MR analyses relied on instruments for clinically coded magnesium disorders rather than continuous magnesium levels, potentially introducing weak-instrument bias. Moreover, most GWAS participants were of European ancestry, further limiting external validity. Although no major pleiotropy was detected, residual bias cannot be entirely ruled out. Finally, the inverse LDL-C finding should be interpreted cautiously given its inconsistency with established cardiovascular evidence. Validation in multiethnic, modern cohorts and across life-course exposure windows is warranted to confirm these findings and enhance their generalizability.

Future research should validate MDS thresholds and temporal dynamics in prospective cohorts and evaluate whether targeted magnesium repletion improves vascular function and ED outcomes in randomized settings. Mechanistic studies on nitric oxide signaling, oxidative stress, and inflammation, together with improved genetic instruments for continuous magnesium status and analyses across diverse ancestries, will be critical to refine causal inference and broaden generalizability.

## 5. Conclusions

Higher MDS was associated with increased odds of ED among U.S. adult men, with a nonlinear increasing pattern. Although univariable MR did not support a direct causal effect of magnesium metabolism disorders on ED, the modest MVMR finding suggests that magnesium metabolism may be involved in ED within a broader cardiometabolic context. Overall, MDS may serve as a potential risk marker for ED, but prospective and interventional studies are needed to confirm its clinical relevance.

## Acknowledgments

We thank the participants and staff of NHANES for collecting and maintaining the survey data. We acknowledge the investigators and participants of the MVP, FinnGen, and the UK Biobank, and the curators of the GWAS Catalog and OpenGWAS, for making GWAS summary statistics publicly available. We also thank colleagues for constructive feedback on study design and analysis. The authors declare that no funding was received for this study and that they have no conflicts of interest.

## Author contributions

**Conceptualization:** Zhexin Zhang, Chengyi Liu.

**Data curation:** Zhexin Zhang.

**Formal analysis:** Zhexin Zhang, Mo Yan, Siyuan Wu, Yong Wu.

**Investigation:** Siyuan Wu, Yong Wu, Xuexue Hao.

**Methodology:** Mo Yan, Siyuan Wu.

**Software:** Mo Yan, Tongxi Li, Xuexue Hao.

**Supervision:** Chengyi Liu.

**Validation:** Yuezheng Li.

**Visualization:** Tongxi Li, Yuezheng Li.

**Writing – original draft:** Zhexin Zhang.

**Writing – review & editing:** Mo Yan, Chengyi Liu.



















## References

[1] Al-Madhagi H Tarabishi AA. Nutritional aphrodisiacs: biochemistry and pharmacology. Curr Res Food Sci. 2024;9:100783. 38974844 10.1016/j.crfs.2024.100783 PMC11225857

[2] Aytaç IA Mckinlay JB Krane RJ. The likely worldwide increase in erectile dysfunction between 1995 and 2025 and some possible policy consequences. BJU Int. 1999;84:50–6. 10444124 10.1046/j.1464-410x.1999.00142.x

[3] Lisco G Triggiani V Bartolomeo N. The role of male hypogonadism, aging, and chronic diseases in characterizing adult and elderly men with erectile dysfunction: a cross-sectional study. Basic Clin Androl. 2023;33:5. 37020191 10.1186/s12610-022-00182-8 PMC10077617

[4] Kostov K Halacheva L. Role of magnesium deficiency in promoting atherosclerosis, endothelial dysfunction, and arterial stiffening as risk factors for hypertension. Int J Mol Sci . 2018;19:1724. 29891771 10.3390/ijms19061724 PMC6032400

[5] Ashique S Kumar S Hussain A. A narrative review on the role of magnesium in immune regulation, inflammation, infectious diseases, and cancer. J Health Popul Nutr. 2023;42:74. 37501216 10.1186/s41043-023-00423-0 PMC10375690

[6] Li D Cai Z Pan Z Yang Y Zhang J. The effects of vitamin and mineral supplementation on women with gestational diabetes mellitus. BMC Endocr Disord. 2021;21:106. 34030674 10.1186/s12902-021-00712-x PMC8145819

[7] Benli E Çirakoğlu A Ayyildiz SN. Comparison of serum magnesium levels in patients with erectile dysfunction and healthy peers. J Acad Res Med. 2018;8:25–9.

[8] Workinger JL Doyle Robert P Bortz J. Challenges in the diagnosis of magnesium status. Nutrients. 2018;10:1202. 30200431 10.3390/nu10091202 PMC6163803

[9] Liu T Wang J Ren C Yu R Fu C. Association between magnesium depletion score and atherosclerotic cardiovascular disease: findings from NHANES 2005 to 2018. Medicine (Baltimore). 2025;104:e43914. 40826784 10.1097/MD.0000000000043914 PMC12367052

[10] Wang X Zeng Z Wang X. Magnesium depletion score and metabolic syndrome in US adults: analysis of NHANES 2003 to 2018. J Clin Endocrinol Metab. 2024;109:e2324–33. 38366015 10.1210/clinem/dgae075 PMC11570370

[11] Liu C Feng Y Ping F. Association of magnesium depletion score with all-cause and cardiovascular mortality in hyperlipidemia adults: a large nationwide population-based study. J Health Popul Nutr. 2025;44:275. 40754582 10.1186/s41043-025-01032-9 PMC12318386

[12] Lovegrove CE Howles SA Furniss D Holmes MV. Causal inference in health and disease: a review of the principles and applications of Mendelian randomization. J Bone Miner Res. 2024;39:1539–52. 39167758 10.1093/jbmr/zjae136 PMC11523132

[13] Lawlor DA. Commentary: two-sample mendelian randomization: opportunities and challenges. Int J Epidemiol. 2016;45:908–15. 27427429 10.1093/ije/dyw127 PMC5005949

[14] Belan M Charmet T Schaeffer L. SARS-CoV-2 exposures of healthcare workers from primary care, long-term care facilities and hospitals: a nationwide matched case-control study. Clin Microbiol Infect. 2022;28:1471–6. 35777605 10.1016/j.cmi.2022.05.038 PMC9239704

[15] Skrivankova VW Richmond RC Woolf BAR. Strengthening the reporting of observational studies in epidemiology using mendelian randomization: the STROBE-MR statement. JAMA. 2021;326:1614–21. 34698778 10.1001/jama.2021.18236

[16] Wu X Zhang Y Jiang H Zhang X. Monocyte-to-high-density lipoprotein cholesterol ratio and the risk of erectile dysfunction: a study from NHANES 2001-2004. Sex Med. 2024;12:qfae025. 38715577 10.1093/sexmed/qfae025 PMC11074004

[17] Ruan Z Xie X Yu H Liu R Jing W Lu T. Association between dietary inflammation and erectile dysfunction among US adults: a cross-sectional analysis of the national health and nutrition examination survey 2001-2004. Front Nutr. 2022;9:930272. 36438746 10.3389/fnut.2022.930272 PMC9691656

[18] Ye L Zhang C Duan Q Shao Y Zhou J. Association of magnesium depletion score with cardiovascular disease and its association with longitudinal mortality in patients with cardiovascular disease. J Am Heart Assoc. 2023;12:e030077. 37681518 10.1161/JAHA.123.030077 PMC10547298

[19] Inker LA Eneanya ND Coresh J. New creatinine- and cystatin C-based equations to estimate GFR without Race. N Engl J Med. 2021;385:1737–49. 34554658 10.1056/NEJMoa2102953 PMC8822996

[20] Cunningham TJ Croft JB Liu Y Lu H Eke PI Giles WH. Vital signs: racial disparities in age-specific mortality among blacks or African Americans - United States, 1999-2015. MMWR Morb Mortal Wkly Rep. 2017;66:444–56. 28472021 10.15585/mmwr.mm6617e1 PMC5687082

[21] Akbar Z Shi Z. Dietary patterns and circadian syndrome among adults attending NHANES 2005-2016. Nutrients. 2023;15:3396. 37571333 10.3390/nu15153396 PMC10421411

[22] Grundy SM Stone NJ Bailey AL. 2018 AHA/ACC/AACVPR/AAPA/ABC/ACPM/ADA/AGS/APhA/ASPC/NLA/PCNA guideline on the management of blood cholesterol: a report of the American College of Cardiology/American heart association task force on clinical practice guidelines. Circulation. 2019;139:e1082–143. 30586774 10.1161/CIR.0000000000000625 PMC7403606

[23] Verma A Huffman JE Rodriguez A. Diversity and scale: genetic architecture of 2068 traits in the VA Million Veteran Program. Science. 2024;385:eadj1182. 39024449 10.1126/science.adj1182 PMC12857194

[24] Yengo L Sidorenko J Kemper KE. Meta-analysis of genome-wide association studies for height and body mass index in ∼700000 individuals of European ancestry. Hum Mol Genet. 2018;27:3641–9. 30124842 10.1093/hmg/ddy271 PMC6488973

[25] Loh PR Kichaev G Gazal S Schoech AP Price AL. Mixed-model association for biobank-scale datasets. Nat Genet. 2018;50:906–8. 29892013 10.1038/s41588-018-0144-6 PMC6309610

[26] Sakaue S Kanai M Tanigawa Y. A cross-population atlas of genetic associations for 220 human phenotypes. Nat Genet. 2021;53:1415–24. 34594039 10.1038/s41588-021-00931-x PMC12208603

[27] Didelez V Sheehan N. Mendelian randomization as an instrumental variable approach to causal inference. Stat Methods Med Res. 2007;16:309–30. 17715159 10.1177/0962280206077743

[28] Hemani G Zheng J Elsworth B. The MR-Base platform supports systematic causal inference across the human phenome. Elife. 2018;7:e34408. 29846171 10.7554/eLife.34408 PMC5976434

[29] Burgess S Thompson SG; CRP CHD Genetics Collaboration. Avoiding bias from weak instruments in Mendelian randomization studies. Int J Epidemiol. 2011;40:755–64. 21414999 10.1093/ije/dyr036

[30] Hemani G Tilling K Davey Smith G. Orienting the causal relationship between imprecisely measured traits using GWAS summary data. PLoS Genet. 2017;13:e1007081. 29149188 10.1371/journal.pgen.1007081 PMC5711033

[31] Lusa L Ahlin C. Restricted cubic splines for modelling periodic data. PLoS One. 2020;15:e0241364. 33112926 10.1371/journal.pone.0241364 PMC7592770

[32] Lin J Zhou J Xu Y. Potential drug targets for multiple sclerosis identified through Mendelian randomization analysis. Brain. 2023;146:3364–72. 36864689 10.1093/brain/awad070 PMC10393411

[33] Kim DJ Ha TW Jung HU. Characterisation of insomnia as an environmental risk factor for asthma via Mendelian randomization and gene environment interaction. Sci Rep. 2021;11:21813. 34750467 10.1038/s41598-021-01291-6 PMC8576024

[34] Crick DCP Sanderson E Jones H. Glycoprotein acetyls and depression: Testing for directionality and potential causality using longitudinal data and Mendelian randomization analyses. J Affect Disord. 2023;335:431–9. 37196932 10.1016/j.jad.2023.05.033 PMC7615476

[35] Ni Y Zhang Y Sun J Zhao L Wu B Ye J. The effect of antioxidant dietary supplements and diet-derived circulating antioxidants on vitiligo outcome: evidence from genetic association and comprehensive Mendelian randomization. Front Nutr. 2023;10:1280162. 38274214 10.3389/fnut.2023.1280162 PMC10808665

[36] Dominguez LJ Veronese N Barbagallo M. Magnesium and hypertension in old age. Nutrients. 2020;13:139. 33396570 10.3390/nu13010139 PMC7823889

[37] Li W Jiao Y Wang L. Association of serum magnesium with insulin resistance and type 2 diabetes among adults in China. Nutrients. 2022;14:1799. 35565766 10.3390/nu14091799 PMC9104014

[38] Drenthen LCA De Baaij JHF Rodwell L Van Herwaarden AE Tack CJ De Galan BE. Oral magnesium supplementation does not affect insulin sensitivity in people with insulin-treated type 2 diabetes and a low serum magnesium: a randomised controlled trial. Diabetologia. 2024;67:52–61. 37922013 10.1007/s00125-023-06029-9 PMC10709477

[39] Jaime Garcia D Chagnot A Wardlaw JM Montagne A. A scoping review on biomarkers of endothelial dysfunction in small vessel disease: molecular insights from human studies. Int J Mol Sci . 2023;24:13114. 37685924 10.3390/ijms241713114 PMC10488088

[40] Fan L Zhu X Rosanoff A. Magnesium Depletion Score (MDS) predicts risk of systemic inflammation and cardiovascular mortality among US Adults. J Nutr. 2021;151:2226–35. 34038556 10.1093/jn/nxab138 PMC8349125

[41] Gong H Lin X Huang S. Association between magnesium depletion score and prostate cancer. Sci Rep. 2025;15:4801. 39922926 10.1038/s41598-025-89506-y PMC11807168

[42] Tian Z Qu S Chen Y. Associations of the magnesium depletion score and magnesium intake with diabetes among US adults: an analysis of the National Health and Nutrition Examination Survey 2011-2018. Epidemiol Health. 2024;46:e2024020. 38271961 10.4178/epih.e2024020 PMC11099598

[43] Toprak O Sari Y Koç A Sari E Kirik A. The impact of hypomagnesemia on erectile dysfunction in elderly, non-diabetic, stage 3 and 4 chronic kidney disease patients: a prospective cross-sectional study. Clin Interv Aging. 2017;12:437–44. 28280316 10.2147/CIA.S129377 PMC5340248

[44] Yu Z Pan Z Cai R. Concentration of selected serum trace elements in male patients with diabetic erectile dysfunction: a case-control study. Am J Mens Health. 2025;19:15579883241307526. 39991892 10.1177/15579883241307526 PMC11863248

[45] Dewitte M Bettocchi C Carvalho J. A psychosocial approach to erectile dysfunction: position statements from the European society of sexual medicine (ESSM). Sex Med. 2021;9:100434–100434. 34626919 10.1016/j.esxm.2021.100434 PMC8766276

[46] Meng F Liao X Chen H. Bibliometric and visualization analysis of literature relating to diabetic erectile dysfunction. Front Endocrinol (Lausanne). 2022;13:1091999. 36568113 10.3389/fendo.2022.1091999 PMC9780376

[47] Cao S Hu X Shao Y. Relationship between weight-adjusted-waist index and erectile dysfunction in the United State: results from NHANES 2001-2004. Front Endocrinol (Lausanne). 2023;14:1128076. 37181040 10.3389/fendo.2023.1128076 PMC10167952

[48] Poznyak AV Sukhorukov VN Eremin II Nadelyaeva II Gutyrchik NA Orekhov AN. HDL-based therapy: vascular protection at all stages. Biomedicines. 2023;11:711. 36979690 10.3390/biomedicines11030711 PMC10045384

[49] Sanderson E. Multivariable mendelian randomization and mediation. Cold Spring Harb Perspect Med. 2021;11:a038984. 32341063 10.1101/cshperspect.a038984 PMC7849347

[50] Corona G Goulis DG Huhtaniemi I. European Academy of Andrology (EAA) guidelines on investigation, treatment and monitoring of functional hypogonadism in males: endorsing organization: European society of endocrinology. Andrology. 2020;8:970–87. 32026626 10.1111/andr.12770

