# Supplementary material for: Magnesium depletion score and erectile dysfunction: A cross-sectional and Mendelian randomization study
Source: Medicine (Baltimore). 2026 Jul 24;105(30):e49938. doi: 10.1097/MD.0000000000049938 (PMC13406066; doi:10.1097/MD.0000000000049938)
Supplement: Supplementary file 1 [file medi-105-e49938-s001.docx]

Table S1. Summary of genome-wide association studies included in this study.

| **Phenotype** | **GWAS accession or endpoint ID** | **GWAS data source** | **Cohort(s)** | **Total sample size** | **Sex** | **Ancestry** |
| --- | --- | --- | --- | --- | --- | --- |
| Erectile dysfunction | GCST90476161 | Verma A et al. 2024 | US, Million Veteran Program | 382,509 | Male | European |
| Disorders of magnesium metabolism | E4_IV Endocrine, nutritional and metabolic diseases | FinnGen-R12 | FinnGen | 420,066 | Both sexes | European |
| BMI | ukb-b-2303 | Elsworth B et al. 2018 | UK Biobank | 454,884 | Both sexes | European |
| Type 2 diabetes | ebi-a-GCST90029024 | Loh P-R et al. 2018 | UK Biobank | 468,298 | Both sexes | European |
| HDL-C | ebi-a-GCST90018956 | Sakaue S et al. 2021 | UK Biobank | 315,133 | Both sexes | European |
| LDL-C | ebi-a-GCST90018961 | Sakaue S et al. 2021 | UK Biobank | 343,621 | Both sexes | European |

Abbreviations: BMI, body mass index; ED, erectile dysfunction; HDL-C, high-density lipoprotein cholesterol; LDL-C, low-density lipoprotein cholesterol; GWAS, genome-wide association study.
