# Supplementary material for: Magnesium depletion score and erectile dysfunction: A cross-sectional and Mendelian randomization study
Source: Medicine (Baltimore). 2026 Jul 24;105(30):e49938. doi: 10.1097/MD.0000000000049938 (PMC13406066; doi:10.1097/MD.0000000000049938)
Supplement: Supplementary file 2 [file medi-105-e49938-s002.docx]

Table S2. Association between the magnesium depletion score and erectile dysfunction.

| Model | Variable | Category | OR (95% CI) | *P* value |
| --- | --- | --- | --- | --- |
| Model 1 | MDS |  | 2.96 (2.65, 3.30) | < 0.001 |
| Model 2 | MDS |  | 1.52 (1.32, 1.74) | < 0.001 |
|  | Age |  | 1.09 (1.08, 1.09) | < 0.001 |
|  | Race | Mexican American | Reference |  |
|  |  | Non-Hispanic Black | 0.66 (0.48, 0.92) | 0.023 |
|  |  | Non-Hispanic White | 0.67 (0.47, 0.96) | 0.043 |
|  |  | Other Hispanic | 1.18 (0.62, 2.26) | 0.622 |
|  |  | Other Race | 0.84 (0.37, 1.90) | 0.682 |
|  | Education | Less than 9th grade | Reference |  |
|  |  | 9-11th grade | 1.01 (0.59, 1.70) | 0.984 |
|  |  | High school graduate | 0.52 (0.32, 0.85) | 0.017 |
|  |  | Some college or AA degree | 0.54 (0.32, 0.91) | 0.031 |
|  |  | College graduate or above | 0.54 (0.35, 0.85) | 0.014 |
|  | PIR |  | 0.86 (0.81, 0.92) | < 0.001 |
| Model 3 | MDS |  | 1.44 (1.25, 1.66) | < 0.001 |
|  | Age |  | 1.08 (1.07, 1.09) | < 0.001 |
|  | Race | Mexican American | Reference |  |
|  |  | Non-Hispanic Black | 0.66 (0.48, 0.92) | 0.027 |
|  |  | Non-Hispanic White | 0.65 (0.46, 0.92) | 0.032 |
|  |  | Other Hispanic | 1.24 (0.65, 2.38) | 0.530 |
|  |  | Other Race | 0.86 (0.40, 1.83) | 0.703 |
|  | Education | Less than 9th grade | Reference |  |
|  |  | 9-11th grade | 1.04 (0.61, 1.78) | 0.880 |
|  |  | High school graduate | 0.54 (0.33, 0.88) | 0.029 |
|  |  | Some college or AA degree | 0.57 (0.34, 1.00) | 0.052 |
|  |  | College graduate or above | 0.66 (0.42, 1.03) | 0.087 |
|  | PIR |  | 0.87 (0.81, 0.93) | 0.002 |
|  | WC |  | 1.02 (1.01, 1.03) | 0.003 |
|  | Smoking | No | Reference |  |
|  |  | Yes | 1.31 (1.07, 1.60) | 0.019 |
|  | Moderate activity | No | Reference |  |
|  |  | Yes | 0.91 (0.73, 1.12) | 0.385 |
|  | Vigorous activity | No | Reference |  |
|  |  | Yes | 0.71 (0.53, 0.96) | 0.042 |
|  | Total energy intake |  | 1.00 (1.00, 1.00) | 0.059 |
|  | Dietary fiber intake |  | 1.01 (0.99, 1.02) | 0.344 |
| Model 4 | MDS |  | 1.40 (1.22, 1.61) | 0.002 |
|  | Age |  | 1.08 (1.07, 1.09) | < 0.001 |
|  | Race | Mexican American | Reference |  |
|  |  | Non-Hispanic Black | 0.61 (0.43, 0.88) | 0.032 |
|  |  | Non-Hispanic White | 0.65 (0.45, 0.94) | 0.056 |
|  |  | Other Hispanic | 1.11 (0.56, 2.20) | 0.777 |
|  |  | Other Race | 0.75 (0.34, 1.65) | 0.495 |
|  | Education | Less than 9th grade | Reference |  |
|  |  | 9-11th grade | 1.02 (0.58, 1.80) | 0.943 |
|  |  | High school graduate | 0.55 (0.33, 0.91) | 0.053 |
|  |  | Some college or AA degree | 0.55 (0.32, 0.93) | 0.061 |
|  |  | College graduate or above | 0.65 (0.41, 1.03) | 0.106 |
|  | PIR |  | 0.87 (0.81, 0.94) | 0.008 |
|  | WC |  | 1.01 (1.00, 1.02) | 0.037 |
|  | Smoking | No | Reference |  |
|  |  | Yes | 1.30 (1.06, 1.60) | 0.042 |
|  | Moderate activity | No | Reference |  |
|  |  | Yes | 0.92 (0.74, 1.15) | 0.488 |
|  | Vigorous activity | No | Reference |  |
|  |  | Yes | 0.74 (0.55, 0.99) | 0.084 |
|  | Total energy intake |  | 1.00 (1.00, 1.00) | 0.137 |
|  | Dietary fiber intake |  | 1.00 (0.99, 1.01) | 0.551 |
|  | HDL-C |  | 1.00 (0.99, 1.01) | 0.577 |
|  | TG |  | 1.00 (1.00, 1.00) | 0.825 |
|  | Hypertension | No | Reference |  |
|  |  | Yes | 1.02 (0.82, 1.28) | 0.838 |
|  | Diabetes | No | Reference |  |
|  |  | Yes | 2.19 (1.58, 3.03) | 0.002 |
|  | CRP |  | 1.03 (0.93, 1.13) | 0.603 |
|  | Mental health status | No | Reference |  |
|  |  | Yes | 1.88 (1.29, 2.75) | 0.014 |
| Model 5 | MDS |  | 1.37 (1.19, 1.57) | 0.007 |
|  | Age |  | 1.08 (1.07, 1.09) | < 0.001 |
|  | Race | Mexican American | Reference |  |
|  |  | Non-Hispanic Black | 0.61 (0.43, 0.88) | 0.044 |
|  |  | Non-Hispanic White | 0.64 (0.45, 0.92) | 0.061 |
|  |  | Other Hispanic | 1.11 (0.57, 2.17) | 0.778 |
|  |  | Other Race | 0.75 (0.34, 1.66) | 0.513 |
|  | Education | Less than 9th grade | Reference |  |
|  |  | 9-11th grade | 1.03 (0.58, 1.82) | 0.924 |
|  |  | High school graduate | 0.56 (0.35, 0.92) | 0.069 |
|  |  | Some college or AA degree | 0.56 (0.33, 0.95) | 0.085 |
|  |  | College graduate or above | 0.68 (0.43, 1.08) | 0.161 |
|  | PIR |  | 0.87 (0.81, 0.94) | 0.015 |
|  | WC |  | 1.01 (1.00, 1.02) | 0.048 |
|  | Smoking | No | Reference |  |
|  |  | Yes | 1.28 (1.03, 1.58) | 0.072 |
|  | Moderate activity | No | Reference |  |
|  |  | Yes | 0.91 (0.73, 1.13) | 0.418 |
|  | Vigorous activity | No | Reference |  |
|  |  | Yes | 0.74 (0.56, 0.99) | 0.095 |
|  | Total energy intake |  | 1.00 (1.00, 1.00) | 0.168 |
|  | Dietary fiber intake |  | 1.00 (0.99, 1.01) | 0.548 |
|  | HDL-C |  | 1.00 (1.00, 1.01) | 0.454 |
|  | TG |  | 1.00 (1.00, 1.00) | 0.815 |
|  | Hypertension | No | Reference |  |
|  |  | Yes | 1.01 (0.81, 1.27) | 0.916 |
|  | Diabetes | No | Reference |  |
|  |  | Yes | 2.17 (1.56, 3.01) | 0.006 |
|  | CRP |  | 1.03 (0.93, 1.14) | 0.603 |
|  | Mental health status | No | Reference |  |
|  |  | Yes | 1.82 (1.25, 2.67) | 0.027 |
|  | CHF | No | Reference |  |
|  |  | Yes | 1.33 (0.80, 2.20) | 0.317 |
|  | CHD | No | Reference |  |
|  |  | Yes | 1.54 (1.06, 2.24) | 0.074 |

Note: Values are ORs with 95% CIs from survey-weighted logistic regression models. Model 1 was unadjusted. Model 2 adjusted for age, race, education, and PIR. Model 3 further adjusted for WC, smoking, moderate activity, vigorous activity, total energy intake, and dietary fiber intake. Model 4 further adjusted for HDL-C, TG, hypertension, diabetes, CRP, and mental health status. Model 5 additionally adjusted for CHF and CHD. Reference indicates the reference category. Abbreviations: MDS, magnesium depletion score; ED, erectile dysfunction; OR, odds ratio; CI, confidence interval; PIR, poverty-to-income ratio; WC, waist circumference; HDL-C, high-density lipoprotein cholesterol; TG, triglycerides; CRP, C-reactive protein; CHF, congestive heart failure; CHD, coronary heart disease.
