# Supplementary material for: Magnesium depletion score and erectile dysfunction: A cross-sectional and Mendelian randomization study
Source: Medicine (Baltimore). 2026 Jul 24;105(30):e49938. doi: 10.1097/MD.0000000000049938 (PMC13406066; doi:10.1097/MD.0000000000049938)
Supplement: Supplementary file 3 [file medi-105-e49938-s003.docx]

Table S4. Subgroup analysis of the association between magnesium depletion score and erectile dysfunction.

| Variable | Group | *P* value | OR (95% CI) | *P* for interaction |
| --- | --- | --- | --- | --- |
| Age, years | < 40 | 0.005 | 2.09 (1.30, 3.38) | 0.247 |
|  | 40–59 | < 0.001 | 1.92 (1.49, 2.46) |  |
|  | ≥ 60 | < 0.001 | 1.53 (1.33, 1.77) |  |
| Race | Mexican American | < 0.001 | 2.19 (1.60, 2.99) | 0.109 |
|  | Non-Hispanic Black | < 0.001 | 2.54 (1.93, 3.33) |  |
|  | Non-Hispanic White | < 0.001 | 3.12 (2.76, 3.53) |  |
|  | Other Hispanic | 0.003 | 4.84 (2.13, 10.98) |  |
|  | Other Race | 0.351 | 1.56 (0.63, 3.90) |  |
| Education | Less than 9th grade | < 0.001 | 2.06 (1.61, 2.64) | 0.127 |
|  | 9–11th grade | < 0.001 | 2.54 (1.83, 3.52) |  |
|  | High school graduate | < 0.001 | 2.77 (2.14, 3.58) |  |
|  | Some college or AA degree | < 0.001 | 3.59 (2.83, 4.56) |  |
|  | College graduate or above | < 0.001 | 3.13 (2.41, 4.08) |  |
| WC, cm | < 102 | < 0.001 | 2.96 (2.47, 3.55) | 0.535 |
|  | ≥ 102 | < 0.001 | 2.70 (2.25, 3.25) |  |
| Fiber density, g/1000 kcal | < 14 | 0.003 | 2.28 (1.39, 3.75) | 0.290 |
|  | ≥ 14 | < 0.001 | 3.02 (2.71, 3.37) |  |
| Mental health status | No | < 0.001 | 3.02 (2.70, 3.38) | 0.159 |
|  | Yes | < 0.001 | 2.25 (1.52, 3.33) |  |
| Moderate activity | No | < 0.001 | 2.77 (2.38, 3.23) | 0.207 |
|  | Yes | < 0.001 | 3.10 (2.73, 3.53) |  |
| Vigorous activity | No | < 0.001 | 2.64 (2.32, 3.00) | 0.246 |
|  | Yes | < 0.001 | 3.09 (2.46, 3.88) |  |
| Smoking | No | < 0.001 | 3.18 (2.57, 3.93) | 0.232 |
|  | Yes | < 0.001 | 2.72 (2.38, 3.11) |  |
| HDL-C, mg/dL | ≥ 60 | < 0.001 | 2.30 (1.63, 3.23) | 0.949 |
|  | 40–59.99 | < 0.001 | 3.39 (2.84, 4.06) |  |
|  | < 40 | < 0.001 | 2.66 (2.19, 3.23) |  |
| TG, mg/dL | ≥ 200 | < 0.001 | 3.07 (2.58, 3.65) | 0.379 |
|  | 150–199.99 | < 0.001 | 3.08 (2.19, 4.33) |  |
|  | < 150 | < 0.001 | 2.63 (2.08, 3.33) |  |
| Hypertension | No | < 0.001 | 2.88 (2.31, 3.59) | 0.228 |
|  | Yes | < 0.001 | 2.45 (2.17, 2.78) |  |
| Diabetes | No | < 0.001 | 2.92 (2.65, 3.23) | 0.170 |
|  | Yes | < 0.001 | 2.23 (1.53, 3.26) |  |
| PIR | < 2 | < 0.001 | 2.93 (2.45, 3.50) | 0.099 |
|  | 2–3.99 | < 0.001 | 3.68 (2.99, 4.52) |  |
|  | ≥ 4 | < 0.001 | 2.51 (1.91, 3.28) |  |
| CHF | No | < 0.001 | 2.86 (2.55, 3.21) | 0.308 |
|  | Yes | 0.007 | 2.18 (1.33, 3.59) |  |
| CHD | No | < 0.001 | 2.81 (2.48, 3.18) | 0.140 |
|  | Yes | < 0.001 | 2.17 (1.63, 2.90) |  |

Note: Values are ORs with 95% CIs from survey-weighted logistic regression models. P for interaction was calculated by including a multiplicative interaction term between MDS and each subgroup variable. ORs represent the association per 1-point increase in MDS. Abbreviations: MDS, magnesium depletion score; ED, erectile dysfunction; OR, odds ratio; CI, confidence interval; WC, waist circumference; HDL-C, high-density lipoprotein cholesterol; TG, triglycerides; PIR, poverty-to-income ratio; CHF, congestive heart failure; CHD, coronary heart disease.
