# Supplementary figures and images for: Magnesium depletion score and erectile dysfunction: A cross-sectional and Mendelian randomization study
Source: Medicine (Baltimore). 2026 Jul 24;105(30):e49938. doi: 10.1097/MD.0000000000049938 (PMC13406066; doi:10.1097/MD.0000000000049938)

RCS Analysis (3 knots)

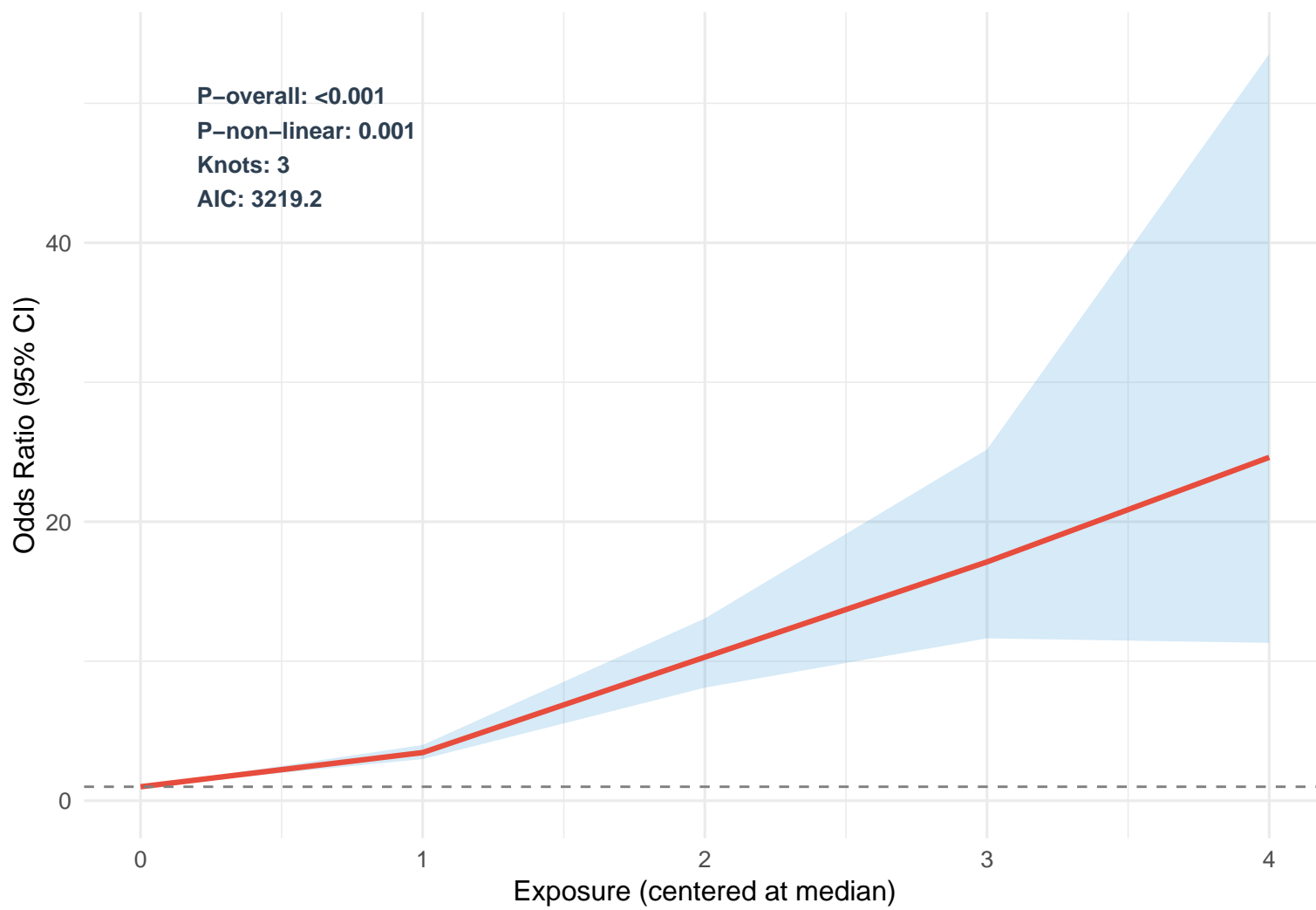

RCS Analysis (4 knots)

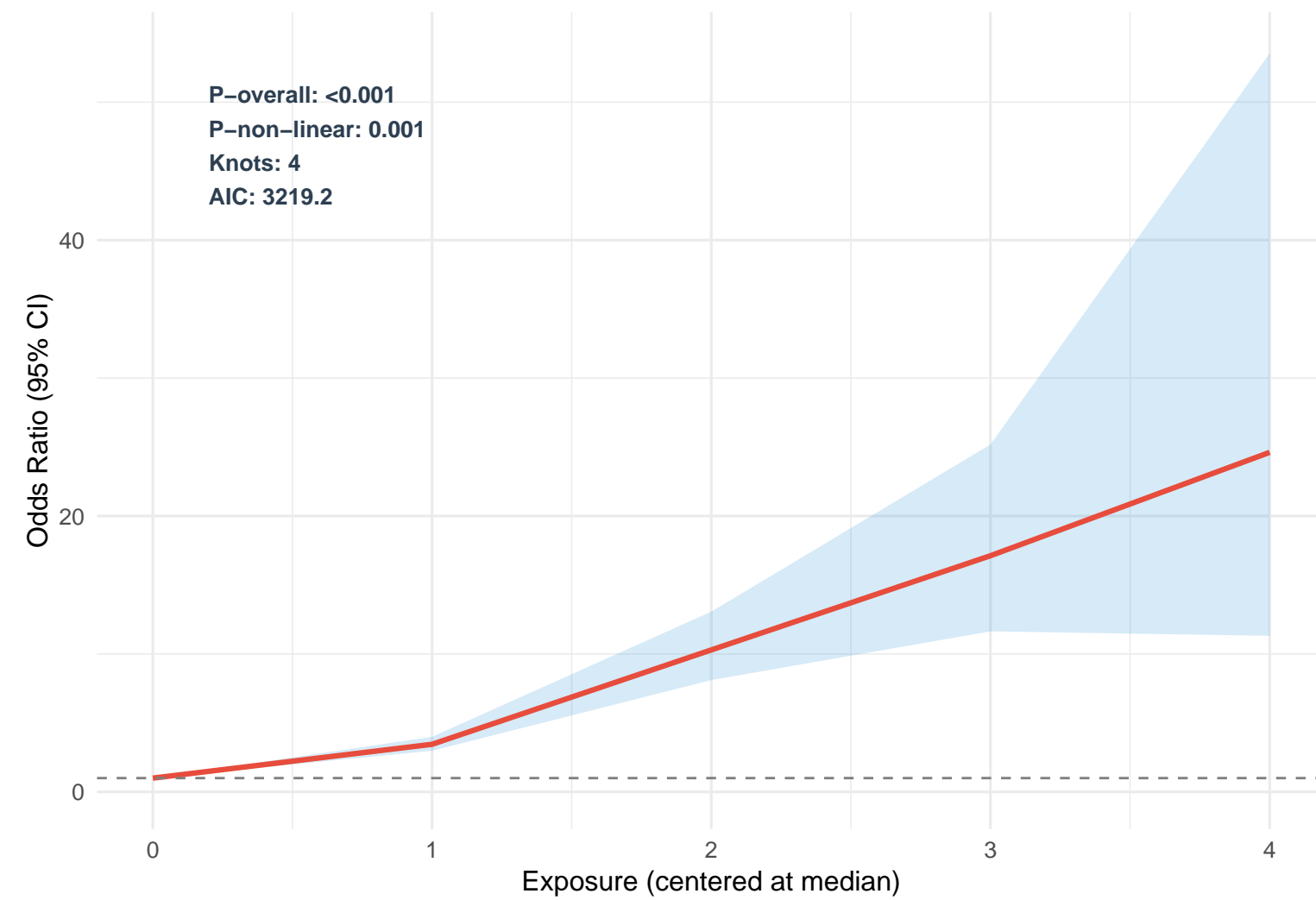

RCS Analysis (5 knots)

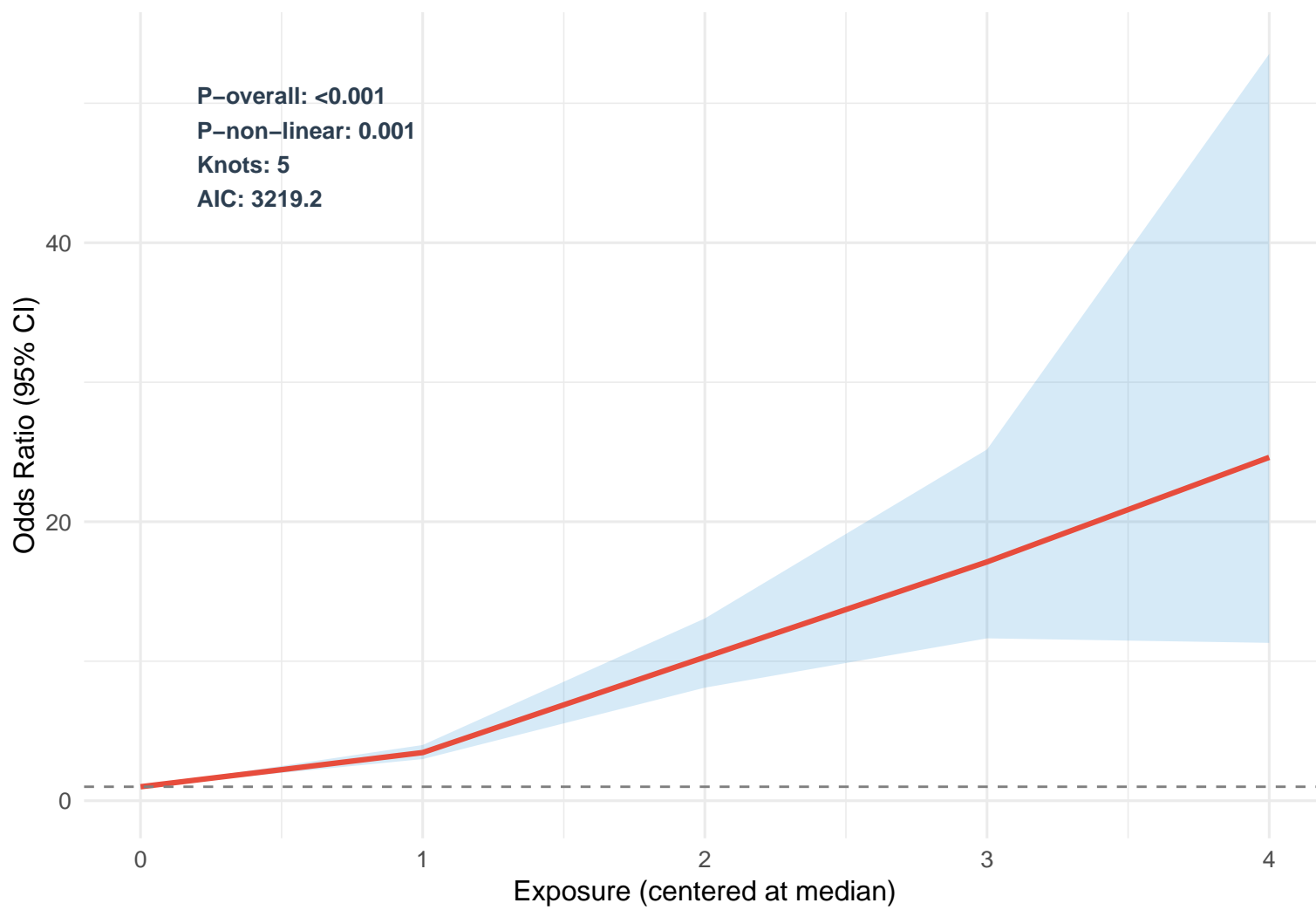

RCS Analysis (6 knots)

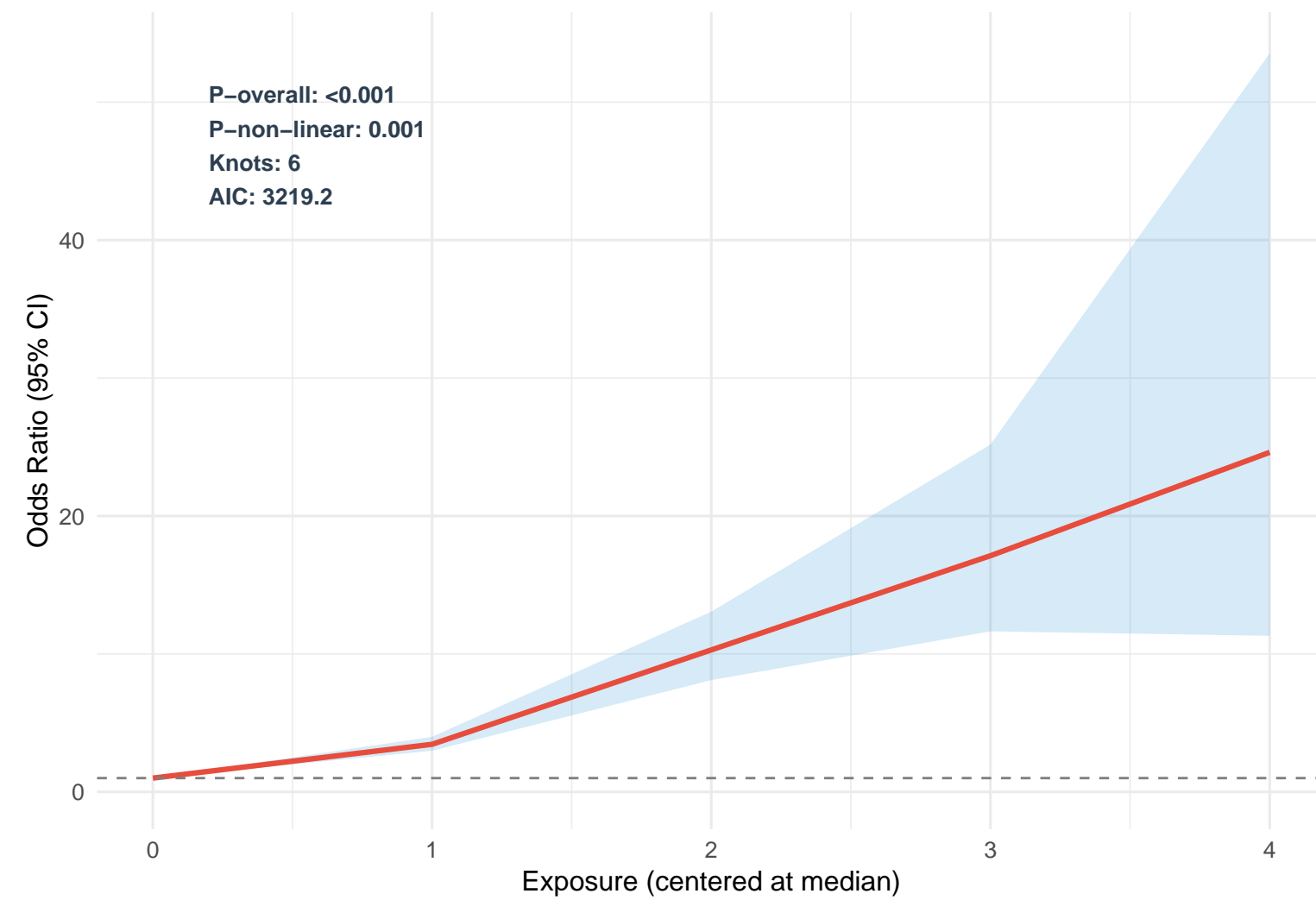

Supplement: Supplementary file 4 [file medi-105-e49938-s004.pdf]
