# Supplementary material for: Magnesium depletion score and erectile dysfunction: A cross-sectional and Mendelian randomization study
Source: Medicine (Baltimore). 2026 Jul 24;105(30):e49938. doi: 10.1097/MD.0000000000049938 (PMC13406066; doi:10.1097/MD.0000000000049938)
Supplement: Supplementary file 5 [file medi-105-e49938-s005.docx]

Table S5. The instrumental variables of exposures in relation to erectile dysfunction.

| Exposure | SNP | Effect allele | Other allele | EAF (exposure) | β (exposure) | SE (exposure) | *P* value (exposure) | EAF (outcome) | Beta (outcome) | SE (outcome) | *P* value (outcome) | Sample size | F statistic | Steiger direction | Steiger *P* value |
| --- | --- | --- | --- | --- | --- | --- | --- | --- | --- | --- | --- | --- | --- | --- | --- |
| Disorders of magnesium metabolism | rs12635649 | A | G | 0.324 | -0.4625 | 0.0994 | 3.28E-06 | 0.271 | 0.0030 | 0.0065 | 6.42E-01 | 420066 | 21.64 | TRUE | 1.22E-02 |
| Disorders of magnesium metabolism | rs146994130 | A | G | 0.072 | 0.6531 | 0.1398 | 3.01E-06 | 0.058 | 0.0059 | 0.0126 | 6.38E-01 | 420066 | 21.81 | TRUE | 4.12E-02 |
| Disorders of magnesium metabolism | rs4664586 | T | C | 0.567 | -0.3915 | 0.0873 | 7.23E-06 | 0.445 | -0.0090 | 0.0061 | 1.40E-01 | 420066 | 20.13 | TRUE | 2.57E-02 |
| Disorders of magnesium metabolism | rs59870127 | G | A | 0.352 | -0.4319 | 0.0961 | 7.02E-06 | 0.410 | 0.0121 | 0.0055 | 2.90E-02 | 420066 | 20.19 | TRUE | 1.94E-02 |
| Disorders of magnesium metabolism | rs72866768 | G | A | 0.035 | 0.8667 | 0.1903 | 5.24E-06 | 0.046 | -0.0070 | 0.0206 | 7.35E-01 | 420066 | 20.75 | TRUE | 5.01E-02 |
| BMI | rs10063055 | T | C | 0.253 | 0.0130 | 0.0023 | 1.40E-08 | 0.238 | 0.0035 | 0.0067 | 6.03E-01 | 454884 | 32.18 | TRUE | 9.46E-03 |
| BMI | rs1017529 | A | C | 0.175 | 0.0152 | 0.0027 | 1.20E-08 | 0.153 | -0.0010 | 0.0136 | 9.42E-01 | 454884 | 32.48 | TRUE | 4.77E-03 |
| BMI | rs10217047 | A | C | 0.096 | 0.0195 | 0.0034 | 1.50E-08 | 0.111 | 0.0187 | 0.0100 | 6.07E-02 | 454884 | 32.07 | TRUE | 7.75E-02 |
| BMI | rs10402950 | C | T | 0.289 | 0.0136 | 0.0022 | 6.40E-10 | 0.287 | -0.0050 | 0.0062 | 4.23E-01 | 454884 | 38.19 | TRUE | 6.77E-03 |
| BMI | rs1048932 | A | C | 0.413 | -0.0155 | 0.0020 | 2.10E-14 | 0.405 | -0.0080 | 0.0056 | 1.53E-01 | 454884 | 58.48 | TRUE | 1.69E-03 |
| BMI | rs10505836 | C | A | 0.860 | 0.0178 | 0.0029 | 7.60E-10 | 0.845 | -0.0090 | 0.0086 | 2.98E-01 | 454884 | 37.85 | TRUE | 1.22E-02 |
| BMI | rs10510025 | T | C | 0.247 | 0.0181 | 0.0023 | 5.00E-15 | 0.242 | 0.0059 | 0.0066 | 3.68E-01 | 454884 | 61.28 | TRUE | 4.81E-04 |
| BMI | rs1064213 | A | G | 0.478 | 0.0149 | 0.0020 | 6.60E-14 | 0.497 | 0.0045 | 0.0055 | 4.15E-01 | 454884 | 56.18 | TRUE | 7.37E-04 |
| BMI | rs10742752 | C | T | 0.612 | 0.0117 | 0.0020 | 1.00E-08 | 0.609 | -0.0030 | 0.0054 | 5.76E-01 | 454884 | 32.76 | TRUE | 8.63E-03 |
| BMI | rs10760277 | T | C | 0.385 | 0.0140 | 0.0021 | 7.90E-12 | 0.389 | 0.0098 | 0.0058 | 8.70E-02 | 454884 | 46.78 | TRUE | 1.04E-02 |
| BMI | rs10799778 | G | T | 0.834 | -0.0185 | 0.0027 | 3.60E-12 | 0.803 | 0.0022 | 0.0077 | 7.74E-01 | 454884 | 48.32 | TRUE | 7.81E-04 |
| BMI | rs10807744 | T | G | 0.182 | -0.0162 | 0.0026 | 3.70E-10 | 0.188 | -0.0050 | 0.0067 | 4.57E-01 | 454884 | 39.24 | TRUE | 4.98E-03 |
| BMI | rs10903791 | A | G | 0.604 | 0.0112 | 0.0020 | 4.50E-08 | 0.594 | 0.0073 | 0.0058 | 2.04E-01 | 454884 | 29.93 | TRUE | 3.61E-02 |
| BMI | rs10927006 | C | T | 0.144 | -0.0171 | 0.0028 | 1.60E-09 | 0.144 | -0.0109 | 0.0078 | 1.60E-01 | 454884 | 36.36 | TRUE | 1.97E-02 |
| BMI | rs10938398 | A | G | 0.433 | 0.0293 | 0.0020 | 4.00E-48 | 0.427 | 0.0123 | 0.0054 | 2.40E-02 | 454884 | 212.48 | TRUE | 4.21E-10 |
| BMI | rs10960294 | T | G | 0.334 | -0.0139 | 0.0021 | 4.80E-11 | 0.322 | 0.0055 | 0.0060 | 3.56E-01 | 454884 | 43.24 | TRUE | 4.39E-03 |
| BMI | rs11000993 | C | T | 0.124 | 0.0213 | 0.0030 | 1.80E-12 | 0.124 | 0.0024 | 0.0082 | 7.71E-01 | 454884 | 49.64 | TRUE | 6.19E-04 |
| BMI | rs11024271 | C | T | 0.623 | 0.0115 | 0.0021 | 2.00E-08 | 0.613 | -0.0100 | 0.0061 | 1.05E-01 | 454884 | 31.52 | TRUE | 5.33E-02 |
| BMI | rs11057072 | G | A | 0.234 | 0.0131 | 0.0024 | 2.50E-08 | 0.221 | 0.0057 | 0.0067 | 3.94E-01 | 454884 | 31.05 | TRUE | 1.74E-02 |
| BMI | rs11079849 | T | C | 0.329 | -0.0205 | 0.0021 | 6.30E-22 | 0.306 | 0.0049 | 0.0062 | 4.28E-01 | 454884 | 92.64 | TRUE | 8.45E-06 |
| BMI | rs11105846 | T | G | 0.368 | -0.0114 | 0.0021 | 4.90E-08 | 0.368 | 0.0023 | 0.0060 | 7.02E-01 | 454884 | 29.77 | TRUE | 1.05E-02 |
| BMI | rs11115160 | A | G | 0.238 | -0.0131 | 0.0024 | 2.40E-08 | 0.230 | -0.0129 | 0.0069 | 5.95E-02 | 454884 | 31.14 | TRUE | 7.12E-02 |
| BMI | rs11122450 | G | T | 0.612 | -0.0116 | 0.0020 | 1.40E-08 | 0.600 | 0.0008 | 0.0054 | 8.82E-01 | 454884 | 32.14 | TRUE | 5.10E-03 |
| BMI | rs111260184 | A | G | 0.503 | 0.0120 | 0.0020 | 2.20E-09 | 0.499 | -0.0030 | 0.0051 | 5.56E-01 | 454884 | 35.82 | TRUE | 5.92E-03 |
| BMI | rs11134679 | G | A | 0.685 | 0.0186 | 0.0021 | 5.50E-18 | 0.664 | -0.0010 | 0.0100 | 9.20E-01 | 454884 | 74.68 | TRUE | 1.77E-05 |
| BMI | rs1114166 | C | T | 0.150 | 0.0156 | 0.0028 | 2.50E-08 | 0.147 | 0.0081 | 0.0094 | 3.88E-01 | 454884 | 31.03 | TRUE | 2.25E-02 |
| BMI | rs11150745 | G | A | 0.318 | -0.0209 | 0.0021 | 2.30E-22 | 0.307 | -0.0060 | 0.0064 | 3.48E-01 | 454884 | 94.63 | TRUE | 1.02E-05 |
| BMI | rs11165643 | T | C | 0.590 | 0.0198 | 0.0020 | 7.90E-23 | 0.576 | 0.0113 | 0.0049 | 2.11E-02 | 454884 | 96.73 | TRUE | 8.24E-05 |
| BMI | rs113079574 | T | C | 0.193 | -0.0161 | 0.0025 | 2.20E-10 | 0.205 | 0.0068 | 0.0071 | 3.34E-01 | 454884 | 40.27 | TRUE | 6.96E-03 |
| BMI | rs113603865 | T | C | 0.212 | 0.0186 | 0.0024 | 2.50E-14 | 0.220 | 0.0084 | 0.0067 | 2.11E-01 | 454884 | 58.11 | TRUE | 1.34E-03 |
| BMI | rs113624107 | A | G | 0.226 | 0.0144 | 0.0024 | 1.50E-09 | 0.215 | 0.0039 | 0.0069 | 5.69E-01 | 454884 | 36.55 | TRUE | 5.73E-03 |
| BMI | rs11525873 | C | T | 0.098 | -0.0240 | 0.0034 | 8.50E-13 | 0.097 | 0.0036 | 0.0098 | 7.14E-01 | 454884 | 51.17 | TRUE | 6.12E-04 |
| BMI | rs116374395 | A | G | 0.035 | 0.0319 | 0.0054 | 3.60E-09 | 0.040 | -0.0020 | 0.0167 | 9.05E-01 | 454884 | 34.82 | TRUE | 3.52E-03 |
| BMI | rs116377258 | G | A | 0.026 | 0.0671 | 0.0063 | 1.60E-26 | 0.028 | 0.0043 | 0.0171 | 8.02E-01 | 454884 | 113.57 | TRUE | 1.37E-07 |
| BMI | rs11691869 | A | C | 0.362 | -0.0197 | 0.0021 | 1.90E-21 | 0.349 | -0.0090 | 0.0057 | 1.17E-01 | 454884 | 90.48 | TRUE | 5.87E-05 |
| BMI | rs11699828 | A | G | 0.036 | -0.0348 | 0.0059 | 2.90E-09 | 0.037 | -0.0070 | 0.0151 | 6.45E-01 | 454884 | 35.24 | TRUE | 5.74E-03 |
| BMI | rs11709402 | G | A | 0.279 | 0.0229 | 0.0022 | 9.50E-25 | 0.269 | 0.0002 | 0.0065 | 9.75E-01 | 454884 | 105.51 | TRUE | 2.09E-07 |
| BMI | rs11714189 | A | G | 0.204 | 0.0135 | 0.0025 | 5.00E-08 | 0.197 | 0.0058 | 0.0071 | 4.16E-01 | 454884 | 29.71 | TRUE | 1.99E-02 |
| BMI | rs117342986 | T | C | 0.026 | 0.0366 | 0.0065 | 1.80E-08 | 0.030 | 0.0020 | 0.0176 | 9.10E-01 | 454884 | 31.65 | TRUE | 5.31E-03 |
| BMI | rs11751591 | A | G | 0.151 | -0.0178 | 0.0028 | 1.50E-10 | 0.146 | -0.0030 | 0.0071 | 6.72E-01 | 454884 | 41.03 | TRUE | 2.30E-03 |
| BMI | rs11757278 | C | T | 0.304 | -0.0147 | 0.0022 | 1.00E-11 | 0.301 | 0.0072 | 0.0061 | 2.35E-01 | 454884 | 46.25 | TRUE | 4.75E-03 |
| BMI | rs118136827 | T | G | 0.281 | -0.0135 | 0.0022 | 1.10E-09 | 0.276 | 0.0033 | 0.0063 | 6.02E-01 | 454884 | 37.17 | TRUE | 4.91E-03 |
| BMI | rs12001437 | C | T | 0.368 | 0.0124 | 0.0021 | 2.20E-09 | 0.375 | 0.0023 | 0.0057 | 6.88E-01 | 454884 | 35.78 | TRUE | 4.76E-03 |
| BMI | rs12033257 | G | A | 0.382 | -0.0155 | 0.0021 | 5.10E-14 | 0.380 | -0.0070 | 0.0058 | 2.29E-01 | 454884 | 56.68 | TRUE | 1.48E-03 |
| BMI | rs1205593 | C | T | 0.759 | -0.0129 | 0.0023 | 3.00E-08 | 0.730 | -0.0040 | 0.0062 | 5.17E-01 | 454884 | 30.69 | TRUE | 1.33E-02 |
| BMI | rs12072739 | G | A | 0.225 | 0.0162 | 0.0024 | 1.20E-11 | 0.228 | -0.0010 | 0.0050 | 8.41E-01 | 454884 | 46.04 | TRUE | 7.75E-04 |
| BMI | rs12140153 | T | G | 0.094 | -0.0336 | 0.0035 | 5.70E-22 | 0.085 | 0.0098 | 0.0107 | 3.59E-01 | 454884 | 92.81 | TRUE | 1.26E-05 |
| BMI | rs12149660 | A | G | 0.115 | -0.0222 | 0.0031 | 1.60E-12 | 0.116 | 0.0065 | 0.0087 | 4.54E-01 | 454884 | 49.98 | TRUE | 1.42E-03 |
| BMI | rs12259464 | A | G | 0.485 | 0.0131 | 0.0020 | 5.70E-11 | 0.474 | -0.0060 | 0.0052 | 2.53E-01 | 454884 | 42.92 | TRUE | 5.81E-03 |
| BMI | rs1229984 | C | T | 0.973 | 0.0389 | 0.0060 | 1.10E-10 | 0.963 | 0.0186 | 0.0151 | 2.19E-01 | 454884 | 41.56 | TRUE | 9.08E-03 |
| BMI | rs12340969 | T | C | 0.441 | -0.0213 | 0.0020 | 3.80E-26 | 0.429 | -0.0060 | 0.0057 | 2.98E-01 | 454884 | 111.87 | TRUE | 1.55E-06 |
| BMI | rs12364470 | G | T | 0.165 | 0.0191 | 0.0027 | 1.10E-12 | 0.160 | 0.0006 | 0.0077 | 9.38E-01 | 454884 | 50.67 | TRUE | 3.60E-04 |
| BMI | rs12427047 | T | C | 0.243 | -0.0171 | 0.0023 | 1.90E-13 | 0.239 | 0.0010 | 0.0065 | 8.77E-01 | 454884 | 54.14 | TRUE | 2.62E-04 |
| BMI | rs12462975 | A | G | 0.330 | 0.0195 | 0.0021 | 8.50E-20 | 0.322 | -0.0070 | 0.0059 | 2.37E-01 | 454884 | 82.92 | TRUE | 6.26E-05 |
| BMI | rs1263629 | G | A | 0.144 | 0.0171 | 0.0028 | 1.90E-09 | 0.144 | 0.0107 | 0.0081 | 1.86E-01 | 454884 | 36.06 | TRUE | 1.93E-02 |
| BMI | rs1266876 | T | C | 0.350 | 0.0141 | 0.0021 | 1.20E-11 | 0.345 | 0.0083 | 0.0059 | 1.57E-01 | 454884 | 45.94 | TRUE | 7.15E-03 |
| BMI | rs12762034 | C | T | 0.077 | 0.0277 | 0.0037 | 1.30E-13 | 0.076 | 0.0117 | 0.0106 | 2.71E-01 | 454884 | 54.85 | TRUE | 1.51E-03 |
| BMI | rs12881629 | G | A | 0.083 | 0.0222 | 0.0036 | 8.30E-10 | 0.084 | 0.0064 | 0.0105 | 5.40E-01 | 454884 | 37.69 | TRUE | 5.47E-03 |
| BMI | rs1296328 | C | A | 0.559 | -0.0181 | 0.0020 | 2.10E-19 | 0.540 | -0.0060 | 0.0054 | 2.70E-01 | 454884 | 81.16 | TRUE | 6.18E-05 |
| BMI | rs13012070 | A | G | 0.228 | -0.0142 | 0.0024 | 1.80E-09 | 0.216 | 0.0008 | 0.0072 | 9.12E-01 | 454884 | 36.14 | TRUE | 2.83E-03 |
| BMI | rs13176429 | C | T | 0.688 | 0.0148 | 0.0021 | 6.10E-12 | 0.689 | 0.0091 | 0.0062 | 1.39E-01 | 454884 | 47.29 | TRUE | 7.17E-03 |
| BMI | rs1320251 | T | C | 0.455 | -0.0177 | 0.0020 | 1.50E-18 | 0.449 | 0.0010 | 0.0055 | 8.56E-01 | 454884 | 77.25 | TRUE | 1.29E-05 |
| BMI | rs1322842 | G | A | 0.609 | -0.0132 | 0.0021 | 1.10E-10 | 0.616 | 0.0016 | 0.0060 | 7.89E-01 | 454884 | 41.68 | TRUE | 1.77E-03 |
| BMI | rs13248187 | C | T | 0.268 | 0.0160 | 0.0023 | 1.50E-12 | 0.265 | -0.0070 | 0.0067 | 2.94E-01 | 454884 | 50.08 | TRUE | 2.63E-03 |
| BMI | rs1330199 | T | G | 0.483 | -0.0119 | 0.0020 | 2.80E-09 | 0.478 | 0.0006 | 0.0054 | 9.11E-01 | 454884 | 35.33 | TRUE | 3.10E-03 |
| BMI | rs13317303 | A | C | 0.150 | -0.0160 | 0.0028 | 1.00E-08 | 0.142 | 0.0130 | 0.0081 | 1.10E-01 | 454884 | 32.82 | TRUE | 4.01E-02 |
| BMI | rs1346841 | A | G | 0.405 | -0.0128 | 0.0020 | 2.90E-10 | 0.406 | -0.0010 | 0.0044 | 8.21E-01 | 454884 | 39.73 | TRUE | 1.91E-03 |
| BMI | rs1360201 | T | C | 0.482 | 0.0132 | 0.0020 | 4.00E-11 | 0.474 | 0.0073 | 0.0055 | 1.86E-01 | 454884 | 43.62 | TRUE | 7.83E-03 |
| BMI | rs140159717 | T | C | 0.082 | -0.0247 | 0.0037 | 3.90E-11 | 0.069 | 0.0046 | 0.0126 | 7.15E-01 | 454884 | 43.67 | TRUE | 1.74E-03 |
| BMI | rs1441264 | A | G | 0.594 | 0.0180 | 0.0021 | 3.70E-18 | 0.591 | 0.0064 | 0.0058 | 2.72E-01 | 454884 | 75.50 | TRUE | 1.39E-04 |
| BMI | rs1451963 | T | G | 0.082 | 0.0229 | 0.0036 | 3.10E-10 | 0.074 | 0.0033 | 0.0108 | 7.60E-01 | 454884 | 39.62 | TRUE | 2.46E-03 |
| BMI | rs1458156 | T | C | 0.488 | 0.0139 | 0.0020 | 3.10E-12 | 0.473 | 0.0073 | 0.0057 | 2.00E-01 | 454884 | 48.64 | TRUE | 4.42E-03 |
| BMI | rs1471740 | C | T | 0.740 | 0.0195 | 0.0023 | 9.90E-18 | 0.742 | 0.0083 | 0.0064 | 1.91E-01 | 454884 | 73.54 | TRUE | 2.52E-04 |
| BMI | rs147568678 | C | T | 0.238 | -0.0142 | 0.0023 | 1.40E-09 | 0.235 | 0.0035 | 0.0069 | 6.10E-01 | 454884 | 36.61 | TRUE | 5.31E-03 |
| BMI | rs1477290 | C | T | 0.137 | 0.0339 | 0.0029 | 4.20E-31 | 0.144 | -0.0020 | 0.0069 | 7.71E-01 | 454884 | 134.50 | TRUE | 9.03E-09 |
| BMI | rs147730268 | T | G | 0.087 | -0.0346 | 0.0036 | 9.80E-22 | 0.083 | -0.0178 | 0.0086 | 3.85E-02 | 454884 | 91.77 | TRUE | 8.64E-05 |
| BMI | rs1503526 | C | T | 0.480 | 0.0156 | 0.0020 | 5.30E-15 | 0.476 | 0.0090 | 0.0056 | 1.09E-01 | 454884 | 61.14 | TRUE | 1.85E-03 |
| BMI | rs1554654 | T | C | 0.471 | -0.0128 | 0.0020 | 1.60E-10 | 0.460 | -0.0060 | 0.0055 | 2.74E-01 | 454884 | 40.93 | TRUE | 7.32E-03 |
| BMI | rs1582931 | A | G | 0.473 | -0.0129 | 0.0020 | 1.60E-10 | 0.474 | 0.0018 | 0.0055 | 7.44E-01 | 454884 | 40.87 | TRUE | 2.13E-03 |
| BMI | rs16846140 | G | A | 0.338 | 0.0138 | 0.0021 | 6.10E-11 | 0.335 | 0.0102 | 0.0059 | 8.62E-02 | 454884 | 42.80 | TRUE | 1.58E-02 |
| BMI | rs16916303 | G | A | 0.120 | -0.0195 | 0.0031 | 3.10E-10 | 0.127 | -0.0080 | 0.0092 | 3.86E-01 | 454884 | 39.61 | TRUE | 7.12E-03 |
| BMI | rs16966801 | G | A | 0.197 | 0.0145 | 0.0025 | 6.70E-09 | 0.203 | -0.0020 | 0.0058 | 7.29E-01 | 454884 | 33.61 | TRUE | 5.30E-03 |
| BMI | rs17056301 | C | T | 0.256 | 0.0132 | 0.0023 | 8.00E-09 | 0.274 | -0.0060 | 0.0068 | 3.76E-01 | 454884 | 33.27 | TRUE | 1.55E-02 |
| BMI | rs17149254 | C | T | 0.805 | -0.0210 | 0.0026 | 3.90E-16 | 0.752 | -0.0080 | 0.0076 | 2.95E-01 | 454884 | 66.30 | TRUE | 5.01E-04 |
| BMI | rs17639546 | A | G | 0.148 | -0.0234 | 0.0028 | 6.70E-17 | 0.138 | 0.0076 | 0.0087 | 3.80E-01 | 454884 | 69.76 | TRUE | 1.85E-04 |
| BMI | rs1778830 | A | G | 0.362 | 0.0141 | 0.0021 | 8.80E-12 | 0.357 | -0.0040 | 0.0062 | 5.21E-01 | 454884 | 46.57 | TRUE | 1.94E-03 |
| BMI | rs1788808 | G | A | 0.495 | -0.0205 | 0.0020 | 8.80E-25 | 0.485 | -0.0040 | 0.0061 | 5.14E-01 | 454884 | 105.65 | TRUE | 1.33E-06 |
| BMI | rs1805123 | G | T | 0.245 | -0.0164 | 0.0023 | 1.30E-12 | 0.237 | -0.0020 | 0.0075 | 7.90E-01 | 454884 | 50.32 | TRUE | 5.89E-04 |
| BMI | rs1852370 | G | A | 0.566 | 0.0216 | 0.0020 | 7.10E-27 | 0.554 | -0.0030 | 0.0058 | 6.05E-01 | 454884 | 115.20 | TRUE | 2.45E-07 |
| BMI | rs1919243 | C | T | 0.488 | 0.0117 | 0.0020 | 7.10E-09 | 0.491 | 0.0045 | 0.0055 | 4.11E-01 | 454884 | 33.51 | TRUE | 1.21E-02 |
| BMI | rs1967772 | A | G | 0.285 | -0.0169 | 0.0022 | 3.10E-14 | 0.271 | -0.0040 | 0.0058 | 4.90E-01 | 454884 | 57.67 | TRUE | 4.39E-04 |
| BMI | rs1999433 | T | C | 0.447 | -0.0118 | 0.0020 | 3.70E-09 | 0.438 | 0.0052 | 0.0056 | 3.54E-01 | 454884 | 34.77 | TRUE | 1.23E-02 |
| BMI | rs2035936 | T | G | 0.056 | 0.0365 | 0.0044 | 9.40E-17 | 0.058 | 0.0128 | 0.0121 | 2.92E-01 | 454884 | 69.08 | TRUE | 2.61E-04 |
| BMI | rs2051559 | C | T | 0.133 | 0.0206 | 0.0029 | 2.70E-12 | 0.130 | 0.0155 | 0.0083 | 6.24E-02 | 454884 | 48.90 | TRUE | 1.04E-02 |
| BMI | rs2076603 | A | G | 0.644 | -0.0123 | 0.0021 | 2.80E-09 | 0.624 | 0.0027 | 0.0060 | 6.52E-01 | 454884 | 35.29 | TRUE | 5.69E-03 |
| BMI | rs2102278 | G | A | 0.323 | 0.0118 | 0.0021 | 2.70E-08 | 0.301 | 0.0007 | 0.0064 | 9.13E-01 | 454884 | 30.89 | TRUE | 5.83E-03 |
| BMI | rs2114210 | A | G | 0.336 | 0.0138 | 0.0021 | 7.10E-11 | 0.332 | -0.0080 | 0.0059 | 1.76E-01 | 454884 | 42.50 | TRUE | 9.38E-03 |
| BMI | rs213518 | C | T | 0.146 | 0.0163 | 0.0028 | 8.40E-09 | 0.137 | -0.0010 | 0.0066 | 8.79E-01 | 454884 | 33.17 | TRUE | 4.30E-03 |
| BMI | rs215634 | G | A | 0.612 | -0.0155 | 0.0021 | 4.10E-14 | 0.593 | 0.0002 | 0.0070 | 9.77E-01 | 454884 | 57.10 | TRUE | 1.37E-04 |
| BMI | rs2164300 | T | C | 0.519 | -0.0122 | 0.0020 | 8.60E-10 | 0.466 | -0.0119 | 0.0052 | 2.17E-02 | 454884 | 37.61 | TRUE | 4.69E-02 |
| BMI | rs2172131 | C | T | 0.579 | -0.0151 | 0.0020 | 6.70E-14 | 0.558 | -0.0090 | 0.0056 | 1.12E-01 | 454884 | 56.16 | TRUE | 3.07E-03 |
| BMI | rs217672 | C | A | 0.272 | 0.0171 | 0.0022 | 2.90E-14 | 0.256 | 0.0083 | 0.0064 | 1.93E-01 | 454884 | 57.81 | TRUE | 1.53E-03 |
| BMI | rs2190887 | T | C | 0.561 | -0.0110 | 0.0020 | 4.10E-08 | 0.543 | 0.0031 | 0.0056 | 5.82E-01 | 454884 | 30.12 | TRUE | 1.27E-02 |
| BMI | rs2192158 | G | A | 0.553 | -0.0154 | 0.0020 | 1.40E-14 | 0.521 | 0.0032 | 0.0057 | 5.73E-01 | 454884 | 59.18 | TRUE | 3.20E-04 |
| BMI | rs2216931 | A | C | 0.662 | 0.0166 | 0.0021 | 2.40E-15 | 0.655 | 0.0048 | 0.0060 | 4.20E-01 | 454884 | 62.73 | TRUE | 3.38E-04 |
| BMI | rs2234458 | T | C | 0.640 | -0.0208 | 0.0021 | 1.10E-23 | 0.645 | -0.0050 | 0.0061 | 4.16E-01 | 454884 | 100.74 | TRUE | 3.50E-06 |
| BMI | rs2271189 | A | G | 0.403 | -0.0161 | 0.0020 | 2.60E-15 | 0.393 | 0.0028 | 0.0056 | 6.18E-01 | 454884 | 62.58 | TRUE | 1.75E-04 |
| BMI | rs2275444 | A | G | 0.726 | -0.0122 | 0.0022 | 4.60E-08 | 0.713 | -0.0050 | 0.0061 | 4.12E-01 | 454884 | 29.87 | TRUE | 1.90E-02 |
| BMI | rs2289379 | T | C | 0.396 | -0.0150 | 0.0020 | 1.80E-13 | 0.390 | 0.0044 | 0.0059 | 4.57E-01 | 454884 | 54.17 | TRUE | 8.80E-04 |
| BMI | rs2307111 | C | T | 0.395 | -0.0278 | 0.0020 | 1.80E-42 | 0.397 | -0.0109 | 0.0056 | 5.21E-02 | 454884 | 186.54 | TRUE | 3.31E-09 |
| BMI | rs2342892 | G | T | 0.516 | -0.0127 | 0.0020 | 1.70E-10 | 0.511 | -0.0100 | 0.0058 | 8.55E-02 | 454884 | 40.82 | TRUE | 2.14E-02 |
| BMI | rs2381404 | C | T | 0.244 | 0.0135 | 0.0023 | 5.50E-09 | 0.238 | 0.0024 | 0.0066 | 7.17E-01 | 454884 | 34.01 | TRUE | 5.71E-03 |
| BMI | rs2398861 | G | A | 0.259 | 0.0182 | 0.0023 | 1.60E-15 | 0.260 | -0.0020 | 0.0057 | 7.24E-01 | 454884 | 63.49 | TRUE | 1.07E-04 |
| BMI | rs2425816 | A | G | 0.415 | 0.0125 | 0.0020 | 7.90E-10 | 0.400 | 0.0057 | 0.0057 | 3.18E-01 | 454884 | 37.80 | TRUE | 9.62E-03 |
| BMI | rs2433733 | A | G | 0.678 | -0.0176 | 0.0021 | 1.00E-16 | 0.650 | -0.0050 | 0.0055 | 3.64E-01 | 454884 | 68.91 | TRUE | 1.70E-04 |
| BMI | rs2482356 | C | T | 0.429 | -0.0112 | 0.0020 | 2.80E-08 | 0.420 | -0.0050 | 0.0059 | 4.01E-01 | 454884 | 30.82 | TRUE | 1.87E-02 |
| BMI | rs252761 | T | G | 0.588 | -0.0113 | 0.0020 | 2.70E-08 | 0.586 | -0.0030 | 0.0065 | 6.47E-01 | 454884 | 30.88 | TRUE | 1.11E-02 |
| BMI | rs2568958 | A | G | 0.604 | 0.0225 | 0.0020 | 1.50E-28 | 0.624 | 0.0041 | 0.0057 | 4.72E-01 | 454884 | 122.83 | TRUE | 1.57E-07 |
| BMI | rs2569993 | C | T | 0.320 | 0.0130 | 0.0021 | 1.20E-09 | 0.324 | 0.0014 | 0.0060 | 8.16E-01 | 454884 | 36.99 | TRUE | 3.08E-03 |
| BMI | rs2606228 | C | A | 0.646 | -0.0135 | 0.0021 | 1.50E-10 | 0.656 | -0.0020 | 0.0069 | 7.72E-01 | 454884 | 41.06 | TRUE | 2.14E-03 |
| BMI | rs2616143 | A | G | 0.320 | -0.0137 | 0.0021 | 1.60E-10 | 0.306 | -0.0100 | 0.0062 | 1.06E-01 | 454884 | 40.96 | TRUE | 1.73E-02 |
| BMI | rs2725371 | G | A | 0.696 | -0.0158 | 0.0022 | 3.50E-13 | 0.683 | -0.0010 | 0.0110 | 9.28E-01 | 454884 | 52.89 | TRUE | 3.18E-04 |
| BMI | rs2781668 | T | C | 0.166 | 0.0148 | 0.0027 | 3.20E-08 | 0.150 | 0.0102 | 0.0082 | 2.16E-01 | 454884 | 30.57 | TRUE | 3.39E-02 |
| BMI | rs2836961 | C | A | 0.376 | 0.0123 | 0.0021 | 2.40E-09 | 0.360 | 0.0102 | 0.0060 | 9.10E-02 | 454884 | 35.62 | TRUE | 3.51E-02 |
| BMI | rs28489620 | A | G | 0.290 | -0.0152 | 0.0022 | 7.70E-12 | 0.288 | 0.0051 | 0.0062 | 4.12E-01 | 454884 | 46.83 | TRUE | 2.42E-03 |
| BMI | rs2861685 | C | T | 0.412 | -0.0169 | 0.0020 | 4.10E-17 | 0.398 | -0.0080 | 0.0057 | 1.65E-01 | 454884 | 70.71 | TRUE | 4.18E-04 |
| BMI | rs28670671 | C | T | 0.286 | -0.0125 | 0.0023 | 4.00E-08 | 0.297 | -0.0050 | 0.0075 | 5.04E-01 | 454884 | 30.14 | TRUE | 1.86E-02 |
| BMI | rs28732167 | A | G | 0.048 | 0.0263 | 0.0046 | 1.30E-08 | 0.048 | -0.0070 | 0.0124 | 5.73E-01 | 454884 | 32.27 | TRUE | 9.37E-03 |
| BMI | rs2920503 | T | C | 0.285 | -0.0142 | 0.0022 | 1.40E-10 | 0.277 | -0.0129 | 0.0056 | 2.13E-02 | 454884 | 41.13 | TRUE | 3.07E-02 |
| BMI | rs2962334 | T | G | 0.020 | 0.0435 | 0.0071 | 8.40E-10 | 0.023 | -0.0010 | 0.0271 | 9.71E-01 | 454884 | 37.65 | TRUE | 2.05E-03 |
| BMI | rs329118 | T | C | 0.419 | -0.0168 | 0.0020 | 1.00E-16 | 0.418 | 0.0032 | 0.0057 | 5.71E-01 | 454884 | 68.91 | TRUE | 9.19E-05 |
| BMI | rs329651 | T | G | 0.804 | 0.0157 | 0.0025 | 4.80E-10 | 0.801 | 0.0138 | 0.0074 | 6.33E-02 | 454884 | 38.75 | TRUE | 3.43E-02 |
| BMI | rs34025316 | T | C | 0.338 | 0.0118 | 0.0021 | 2.60E-08 | 0.325 | 0.0019 | 0.0060 | 7.53E-01 | 454884 | 30.98 | TRUE | 7.94E-03 |
| BMI | rs34153025 | C | T | 0.022 | -0.0384 | 0.0068 | 1.80E-08 | 0.017 | 0.0099 | 0.0224 | 6.57E-01 | 454884 | 31.69 | TRUE | 9.04E-03 |
| BMI | rs34236292 | T | G | 0.327 | -0.0136 | 0.0021 | 1.70E-10 | 0.316 | -0.0010 | 0.0047 | 8.31E-01 | 454884 | 40.75 | TRUE | 1.64E-03 |
| BMI | rs34517439 | A | C | 0.122 | 0.0383 | 0.0031 | 1.30E-35 | 0.111 | -0.0080 | 0.0088 | 3.66E-01 | 454884 | 155.10 | TRUE | 5.15E-09 |
| BMI | rs34811474 | A | G | 0.231 | -0.0283 | 0.0024 | 3.30E-33 | 0.220 | -0.0080 | 0.0065 | 2.23E-01 | 454884 | 144.16 | TRUE | 4.61E-08 |
| BMI | rs349071 | A | G | 0.500 | -0.0129 | 0.0020 | 9.20E-11 | 0.480 | 0.0035 | 0.0057 | 5.40E-01 | 454884 | 41.98 | TRUE | 3.13E-03 |
| BMI | rs35154326 | G | A | 0.274 | -0.0134 | 0.0023 | 2.80E-09 | 0.275 | 0.0099 | 0.0067 | 1.40E-01 | 454884 | 35.30 | TRUE | 2.96E-02 |
| BMI | rs35364449 | T | C | 0.110 | 0.0220 | 0.0032 | 6.70E-12 | 0.112 | 0.0010 | 0.0091 | 9.12E-01 | 454884 | 47.12 | TRUE | 6.24E-04 |
| BMI | rs355393 | G | A | 0.470 | -0.0115 | 0.0020 | 8.50E-09 | 0.441 | -0.0060 | 0.0061 | 3.28E-01 | 454884 | 33.16 | TRUE | 1.83E-02 |
| BMI | rs35972789 | A | C | 0.037 | -0.0291 | 0.0052 | 2.90E-08 | 0.033 | -0.0139 | 0.0239 | 5.60E-01 | 454884 | 30.77 | TRUE | 1.90E-02 |
| BMI | rs36007635 | A | G | 0.138 | -0.0205 | 0.0029 | 1.20E-12 | 0.130 | -0.0030 | 0.0077 | 6.97E-01 | 454884 | 50.43 | TRUE | 6.48E-04 |
| BMI | rs36061954 | T | C | 0.399 | 0.0129 | 0.0020 | 2.50E-10 | 0.397 | -0.0010 | 0.0039 | 7.98E-01 | 454884 | 40.05 | TRUE | 1.83E-03 |
| BMI | rs3751837 | T | C | 0.219 | 0.0143 | 0.0024 | 3.50E-09 | 0.207 | -0.0050 | 0.0067 | 4.57E-01 | 454884 | 34.88 | TRUE | 8.96E-03 |
| BMI | rs3766823 | A | G | 0.172 | 0.0162 | 0.0026 | 7.60E-10 | 0.180 | 0.0125 | 0.0059 | 3.55E-02 | 454884 | 37.86 | TRUE | 2.67E-02 |
| BMI | rs3803286 | G | A | 0.667 | -0.0184 | 0.0021 | 3.10E-18 | 0.655 | -0.0060 | 0.0059 | 3.08E-01 | 454884 | 75.86 | TRUE | 1.04E-04 |
| BMI | rs3807566 | T | G | 0.438 | -0.0126 | 0.0020 | 4.30E-10 | 0.442 | -0.0119 | 0.0052 | 2.20E-02 | 454884 | 38.98 | TRUE | 4.05E-02 |
| BMI | rs3897102 | T | C | 0.411 | 0.0123 | 0.0020 | 1.90E-09 | 0.409 | -0.0050 | 0.0058 | 3.89E-01 | 454884 | 36.08 | TRUE | 9.79E-03 |
| BMI | rs3901286 | A | C | 0.152 | -0.0226 | 0.0028 | 3.70E-16 | 0.147 | -0.0139 | 0.0079 | 7.87E-02 | 454884 | 66.40 | TRUE | 1.36E-03 |
| BMI | rs3902951 | G | T | 0.237 | 0.0145 | 0.0024 | 8.80E-10 | 0.243 | -0.0010 | 0.0078 | 8.98E-01 | 454884 | 37.58 | TRUE | 2.46E-03 |
| BMI | rs3935190 | A | G | 0.537 | -0.0148 | 0.0020 | 2.00E-13 | 0.532 | -0.0050 | 0.0055 | 3.61E-01 | 454884 | 54.02 | TRUE | 1.12E-03 |
| BMI | rs394608 | C | T | 0.538 | 0.0186 | 0.0020 | 2.30E-20 | 0.523 | 0.0034 | 0.0057 | 5.54E-01 | 454884 | 85.48 | TRUE | 1.26E-05 |
| BMI | rs40071 | C | T | 0.179 | -0.0260 | 0.0026 | 1.30E-23 | 0.174 | 0.0014 | 0.0074 | 8.50E-01 | 454884 | 100.28 | TRUE | 6.45E-07 |
| BMI | rs4055791 | T | C | 0.417 | -0.0181 | 0.0020 | 4.40E-19 | 0.400 | 0.0078 | 0.0057 | 1.72E-01 | 454884 | 79.70 | TRUE | 1.43E-04 |
| BMI | rs4148155 | G | A | 0.113 | -0.0224 | 0.0031 | 7.80E-13 | 0.109 | 0.0087 | 0.0089 | 3.25E-01 | 454884 | 51.33 | TRUE | 1.82E-03 |
| BMI | rs4261944 | G | T | 0.365 | 0.0142 | 0.0021 | 7.90E-12 | 0.362 | 0.0014 | 0.0058 | 8.09E-01 | 454884 | 46.79 | TRUE | 8.33E-04 |
| BMI | rs429358 | C | T | 0.154 | -0.0266 | 0.0028 | 5.90E-22 | 0.139 | -0.0070 | 0.0075 | 3.55E-01 | 454884 | 92.75 | TRUE | 9.54E-06 |
| BMI | rs4307239 | G | A | 0.459 | 0.0125 | 0.0020 | 4.60E-10 | 0.453 | -0.0060 | 0.0056 | 2.84E-01 | 454884 | 38.82 | TRUE | 9.39E-03 |
| BMI | rs4456769 | T | C | 0.333 | 0.0147 | 0.0021 | 3.80E-12 | 0.321 | -0.0010 | 0.0041 | 8.06E-01 | 454884 | 48.22 | TRUE | 5.97E-04 |
| BMI | rs4477562 | T | C | 0.129 | 0.0298 | 0.0030 | 3.70E-23 | 0.125 | 0.0120 | 0.0083 | 1.52E-01 | 454884 | 98.25 | TRUE | 1.86E-05 |
| BMI | rs45486197 | A | G | 0.066 | 0.0258 | 0.0041 | 2.40E-10 | 0.066 | 0.0166 | 0.0118 | 1.57E-01 | 454884 | 40.07 | TRUE | 1.49E-02 |
| BMI | rs4613074 | C | T | 0.185 | -0.0236 | 0.0026 | 3.70E-20 | 0.188 | 0.0078 | 0.0071 | 2.69E-01 | 454884 | 84.56 | TRUE | 4.38E-05 |
| BMI | rs4625852 | A | G | 0.205 | -0.0138 | 0.0025 | 2.00E-08 | 0.191 | 0.0040 | 0.0073 | 5.85E-01 | 454884 | 31.48 | TRUE | 1.08E-02 |
| BMI | rs4648450 | A | C | 0.467 | -0.0148 | 0.0020 | 1.30E-13 | 0.466 | -0.0030 | 0.0065 | 6.46E-01 | 454884 | 54.84 | TRUE | 5.15E-04 |
| BMI | rs4658403 | T | C | 0.834 | -0.0187 | 0.0027 | 2.70E-12 | 0.811 | -0.0139 | 0.0076 | 6.89E-02 | 454884 | 48.88 | TRUE | 1.18E-02 |
| BMI | rs4672338 | T | C | 0.336 | 0.0133 | 0.0021 | 2.40E-10 | 0.340 | 0.0066 | 0.0058 | 2.55E-01 | 454884 | 40.10 | TRUE | 8.81E-03 |
| BMI | rs4722398 | T | C | 0.136 | 0.0186 | 0.0029 | 1.40E-10 | 0.133 | 0.0144 | 0.0069 | 3.82E-02 | 454884 | 41.20 | TRUE | 1.96E-02 |
| BMI | rs4764949 | G | A | 0.326 | -0.0180 | 0.0021 | 3.10E-17 | 0.335 | 0.0074 | 0.0060 | 2.14E-01 | 454884 | 71.29 | TRUE | 2.94E-04 |
| BMI | rs4779541 | C | T | 0.467 | 0.0122 | 0.0020 | 1.30E-09 | 0.467 | 0.0050 | 0.0056 | 3.70E-01 | 454884 | 36.83 | TRUE | 9.17E-03 |
| BMI | rs4790292 | A | C | 0.154 | -0.0260 | 0.0028 | 6.90E-21 | 0.148 | -0.0070 | 0.0085 | 4.15E-01 | 454884 | 87.89 | TRUE | 1.82E-05 |
| BMI | rs4832298 | T | C | 0.686 | -0.0160 | 0.0021 | 8.20E-14 | 0.684 | -0.0119 | 0.0054 | 2.65E-02 | 454884 | 55.75 | TRUE | 6.13E-03 |
| BMI | rs4900590 | T | C | 0.324 | 0.0147 | 0.0021 | 5.60E-12 | 0.327 | 0.0128 | 0.0054 | 1.70E-02 | 454884 | 47.48 | TRUE | 1.81E-02 |
| BMI | rs4919197 | T | C | 0.474 | 0.0110 | 0.0020 | 4.80E-08 | 0.459 | 0.0067 | 0.0059 | 2.51E-01 | 454884 | 29.79 | TRUE | 3.27E-02 |
| BMI | rs4929923 | C | T | 0.645 | 0.0191 | 0.0021 | 3.40E-20 | 0.637 | 0.0038 | 0.0057 | 5.04E-01 | 454884 | 84.73 | TRUE | 1.55E-05 |
| BMI | rs4958702 | C | T | 0.572 | -0.0156 | 0.0020 | 9.60E-15 | 0.568 | -0.0020 | 0.0066 | 7.61E-01 | 454884 | 59.98 | TRUE | 1.84E-04 |
| BMI | rs512121 | C | T | 0.192 | -0.0157 | 0.0025 | 6.70E-10 | 0.198 | -0.0060 | 0.0075 | 4.24E-01 | 454884 | 38.10 | TRUE | 7.35E-03 |
| BMI | rs539515 | C | A | 0.205 | 0.0497 | 0.0025 | 7.00E-91 | 0.194 | 0.0055 | 0.0069 | 4.27E-01 | 454884 | 408.70 | TRUE | 7.69E-23 |
| BMI | rs55658481 | A | G | 0.339 | 0.0134 | 0.0021 | 1.80E-10 | 0.334 | 0.0079 | 0.0059 | 1.82E-01 | 454884 | 40.63 | TRUE | 1.15E-02 |
| BMI | rs55707359 | G | T | 0.015 | 0.0528 | 0.0082 | 1.10E-10 | 0.013 | -0.0218 | 0.0246 | 3.76E-01 | 454884 | 41.72 | TRUE | 5.07E-03 |
| BMI | rs55714539 | C | A | 0.344 | 0.0178 | 0.0021 | 3.50E-17 | 0.337 | -0.0080 | 0.0058 | 1.66E-01 | 454884 | 71.05 | TRUE | 3.66E-04 |
| BMI | rs55726687 | A | G | 0.210 | 0.0249 | 0.0024 | 2.70E-24 | 0.197 | 0.0032 | 0.0072 | 6.55E-01 | 454884 | 103.40 | TRUE | 8.87E-07 |
| BMI | rs55769038 | A | G | 0.590 | 0.0162 | 0.0020 | 1.50E-15 | 0.578 | 0.0104 | 0.0048 | 2.97E-02 | 454884 | 63.65 | TRUE | 2.06E-03 |
| BMI | rs558887 | G | A | 0.308 | -0.0133 | 0.0022 | 8.00E-10 | 0.303 | -0.0090 | 0.0063 | 1.56E-01 | 454884 | 37.75 | TRUE | 1.93E-02 |
| BMI | rs559231 | T | G | 0.393 | 0.0129 | 0.0021 | 3.20E-10 | 0.397 | -0.0080 | 0.0057 | 1.62E-01 | 454884 | 39.53 | TRUE | 1.42E-02 |
| BMI | rs56038322 | A | G | 0.311 | 0.0141 | 0.0022 | 6.80E-11 | 0.307 | -0.0010 | 0.0048 | 8.37E-01 | 454884 | 42.59 | TRUE | 1.27E-03 |
| BMI | rs56133507 | G | T | 0.197 | 0.0142 | 0.0025 | 1.40E-08 | 0.189 | -0.0050 | 0.0070 | 4.74E-01 | 454884 | 32.25 | TRUE | 1.20E-02 |
| BMI | rs56203622 | C | T | 0.146 | 0.0180 | 0.0028 | 2.00E-10 | 0.145 | 0.0145 | 0.0082 | 7.76E-02 | 454884 | 40.46 | TRUE | 2.36E-02 |
| BMI | rs56356382 | C | T | 0.192 | -0.0226 | 0.0025 | 6.70E-19 | 0.194 | -0.0119 | 0.0057 | 3.79E-02 | 454884 | 78.86 | TRUE | 3.02E-04 |
| BMI | rs56399737 | T | C | 0.449 | -0.0158 | 0.0020 | 3.60E-15 | 0.434 | -0.0109 | 0.0049 | 2.43E-02 | 454884 | 61.92 | TRUE | 3.00E-03 |
| BMI | rs56803094 | G | A | 0.227 | -0.0149 | 0.0024 | 5.00E-10 | 0.220 | -0.0129 | 0.0068 | 5.85E-02 | 454884 | 38.67 | TRUE | 3.21E-02 |
| BMI | rs56858768 | A | G | 0.297 | 0.0158 | 0.0022 | 5.60E-13 | 0.284 | -0.0040 | 0.0065 | 5.38E-01 | 454884 | 52.00 | TRUE | 9.11E-04 |
| BMI | rs56893062 | G | T | 0.303 | 0.0129 | 0.0022 | 2.40E-09 | 0.293 | 0.0040 | 0.0061 | 5.10E-01 | 454884 | 35.58 | TRUE | 7.35E-03 |
| BMI | rs57488047 | C | T | 0.468 | -0.0152 | 0.0020 | 4.50E-14 | 0.448 | 0.0070 | 0.0058 | 2.24E-01 | 454884 | 56.95 | TRUE | 1.54E-03 |
| BMI | rs57636386 | C | T | 0.084 | -0.0409 | 0.0036 | 1.00E-29 | 0.085 | 0.0089 | 0.0100 | 3.71E-01 | 454884 | 128.19 | TRUE | 1.36E-07 |
| BMI | rs58862095 | T | C | 0.419 | -0.0226 | 0.0020 | 4.90E-29 | 0.394 | -0.0030 | 0.0059 | 6.12E-01 | 454884 | 125.07 | TRUE | 6.88E-08 |
| BMI | rs59227842 | G | A | 0.311 | 0.0231 | 0.0022 | 1.90E-26 | 0.308 | 0.0064 | 0.0062 | 2.99E-01 | 454884 | 113.21 | TRUE | 1.32E-06 |
| BMI | rs59237168 | C | T | 0.216 | -0.0158 | 0.0024 | 8.20E-11 | 0.218 | 0.0017 | 0.0068 | 8.04E-01 | 454884 | 42.21 | TRUE | 1.57E-03 |
| BMI | rs594024 | C | T | 0.554 | -0.0149 | 0.0020 | 1.00E-13 | 0.550 | 0.0074 | 0.0057 | 1.93E-01 | 454884 | 55.35 | TRUE | 2.10E-03 |
| BMI | rs5995843 | G | A | 0.346 | -0.0176 | 0.0021 | 5.00E-17 | 0.342 | 0.0061 | 0.0058 | 2.89E-01 | 454884 | 70.33 | TRUE | 2.11E-04 |
| BMI | rs6023655 | G | A | 0.766 | -0.0143 | 0.0024 | 1.70E-09 | 0.749 | -0.0090 | 0.0067 | 1.84E-01 | 454884 | 36.30 | TRUE | 2.02E-02 |
| BMI | rs60764613 | T | G | 0.145 | 0.0201 | 0.0028 | 1.60E-12 | 0.150 | -0.0020 | 0.0067 | 7.66E-01 | 454884 | 49.96 | TRUE | 5.62E-04 |
| BMI | rs6134916 | T | C | 0.493 | -0.0109 | 0.0020 | 4.60E-08 | 0.484 | 0.0048 | 0.0055 | 3.85E-01 | 454884 | 29.89 | TRUE | 2.04E-02 |
| BMI | rs61813324 | T | C | 0.136 | 0.0290 | 0.0029 | 6.20E-23 | 0.135 | 0.0112 | 0.0080 | 1.65E-01 | 454884 | 97.22 | TRUE | 1.86E-05 |
| BMI | rs61828088 | A | G | 0.110 | 0.0221 | 0.0032 | 2.90E-12 | 0.111 | 0.0086 | 0.0089 | 3.34E-01 | 454884 | 48.76 | TRUE | 2.46E-03 |
| BMI | rs61903695 | G | A | 0.255 | 0.0167 | 0.0023 | 2.70E-13 | 0.239 | 0.0040 | 0.0066 | 5.45E-01 | 454884 | 53.44 | TRUE | 7.13E-04 |
| BMI | rs61992671 | G | A | 0.492 | -0.0161 | 0.0021 | 1.20E-14 | 0.433 | 0.0071 | 0.0067 | 2.86E-01 | 454884 | 59.48 | TRUE | 1.18E-03 |
| BMI | rs62007782 | A | G | 0.265 | -0.0162 | 0.0023 | 6.80E-13 | 0.250 | 0.0008 | 0.0068 | 9.06E-01 | 454884 | 51.61 | TRUE | 3.47E-04 |
| BMI | rs62033408 | G | A | 0.393 | 0.0712 | 0.0020 | 1.00E-200 | 0.394 | 0.0110 | 0.0057 | 5.26E-02 | 454884 | 1218.27 | TRUE | 1.80E-62 |
| BMI | rs62072006 | C | A | 0.145 | 0.0159 | 0.0028 | 2.30E-08 | 0.140 | -0.0070 | 0.0081 | 3.86E-01 | 454884 | 31.23 | TRUE | 1.74E-02 |
| BMI | rs62107261 | C | T | 0.048 | -0.0915 | 0.0046 | 1.70E-86 | 0.047 | -0.0040 | 0.0123 | 7.45E-01 | 454884 | 388.51 | TRUE | 8.00E-23 |
| BMI | rs62241847 | G | A | 0.315 | -0.0125 | 0.0021 | 5.80E-09 | 0.284 | -0.0100 | 0.0063 | 1.17E-01 | 454884 | 33.89 | TRUE | 3.52E-02 |
| BMI | rs62246311 | A | G | 0.102 | 0.0207 | 0.0033 | 2.90E-10 | 0.110 | -0.0109 | 0.0087 | 2.07E-01 | 454884 | 39.73 | TRUE | 1.08E-02 |
| BMI | rs62379271 | G | T | 0.578 | 0.0117 | 0.0020 | 7.30E-09 | 0.556 | -0.0020 | 0.0050 | 6.88E-01 | 454884 | 33.46 | TRUE | 6.04E-03 |
| BMI | rs6265 | T | C | 0.188 | -0.0401 | 0.0025 | 5.90E-56 | 0.189 | -0.0090 | 0.0071 | 2.06E-01 | 454884 | 248.36 | TRUE | 2.33E-13 |
| BMI | rs6430068 | A | G | 0.109 | 0.0187 | 0.0032 | 5.60E-09 | 0.115 | -0.0109 | 0.0090 | 2.24E-01 | 454884 | 33.98 | TRUE | 2.16E-02 |
| BMI | rs6444950 | A | G | 0.238 | 0.0162 | 0.0023 | 3.90E-12 | 0.236 | -0.0050 | 0.0070 | 4.75E-01 | 454884 | 48.18 | TRUE | 1.81E-03 |
| BMI | rs6474856 | T | C | 0.640 | -0.0120 | 0.0021 | 7.80E-09 | 0.626 | -0.0060 | 0.0057 | 2.91E-01 | 454884 | 33.31 | TRUE | 1.73E-02 |
| BMI | rs6531639 | A | G | 0.248 | -0.0140 | 0.0024 | 3.00E-09 | 0.231 | 0.0092 | 0.0070 | 1.89E-01 | 454884 | 35.22 | TRUE | 2.29E-02 |
| BMI | rs6545714 | A | G | 0.601 | -0.0207 | 0.0020 | 2.50E-24 | 0.594 | -0.0070 | 0.0062 | 2.60E-01 | 454884 | 103.60 | TRUE | 6.33E-06 |
| BMI | rs6551304 | G | A | 0.832 | 0.0176 | 0.0027 | 4.40E-11 | 0.826 | 0.0092 | 0.0076 | 2.26E-01 | 454884 | 43.41 | TRUE | 7.37E-03 |
| BMI | rs6560906 | C | T | 0.692 | -0.0122 | 0.0022 | 1.70E-08 | 0.692 | -0.0030 | 0.0058 | 6.05E-01 | 454884 | 31.77 | TRUE | 9.40E-03 |
| BMI | rs6575340 | A | G | 0.636 | 0.0209 | 0.0021 | 9.10E-24 | 0.630 | -0.0070 | 0.0057 | 2.21E-01 | 454884 | 101.02 | TRUE | 8.15E-06 |
| BMI | rs66679256 | T | C | 0.446 | 0.0153 | 0.0020 | 1.90E-14 | 0.440 | 0.0016 | 0.0056 | 7.77E-01 | 454884 | 58.60 | TRUE | 1.90E-04 |
| BMI | rs6669341 | G | A | 0.583 | -0.0173 | 0.0020 | 9.20E-18 | 0.594 | -0.0020 | 0.0053 | 7.07E-01 | 454884 | 73.68 | TRUE | 3.08E-05 |
| BMI | rs6682438 | C | T | 0.673 | 0.0127 | 0.0021 | 1.80E-09 | 0.649 | 0.0024 | 0.0061 | 6.94E-01 | 454884 | 36.15 | TRUE | 4.62E-03 |
| BMI | rs66978877 | C | T | 0.268 | -0.0175 | 0.0023 | 7.90E-15 | 0.258 | -0.0030 | 0.0063 | 6.33E-01 | 454884 | 60.35 | TRUE | 2.22E-04 |
| BMI | rs6705567 | C | T | 0.376 | -0.0145 | 0.0021 | 2.40E-12 | 0.364 | 0.0026 | 0.0060 | 6.65E-01 | 454884 | 49.10 | TRUE | 9.11E-04 |
| BMI | rs6707827 | G | A | 0.704 | 0.0123 | 0.0022 | 1.70E-08 | 0.698 | -0.0070 | 0.0063 | 2.69E-01 | 454884 | 31.76 | TRUE | 2.42E-02 |
| BMI | rs6752979 | A | G | 0.317 | 0.0122 | 0.0021 | 1.10E-08 | 0.318 | -0.0060 | 0.0063 | 3.44E-01 | 454884 | 32.59 | TRUE | 1.78E-02 |
| BMI | rs67609008 | C | T | 0.284 | 0.0175 | 0.0022 | 2.80E-15 | 0.272 | 0.0056 | 0.0064 | 3.79E-01 | 454884 | 62.39 | TRUE | 4.09E-04 |
| BMI | rs6777784 | T | G | 0.617 | 0.0124 | 0.0020 | 1.30E-09 | 0.621 | 0.0064 | 0.0057 | 2.62E-01 | 454884 | 36.76 | TRUE | 1.29E-02 |
| BMI | rs6843852 | T | C | 0.508 | 0.0134 | 0.0020 | 1.70E-11 | 0.519 | 0.0049 | 0.0057 | 3.87E-01 | 454884 | 45.31 | TRUE | 3.17E-03 |
| BMI | rs6909685 | T | C | 0.327 | -0.0146 | 0.0021 | 6.20E-12 | 0.336 | -0.0020 | 0.0059 | 7.36E-01 | 454884 | 47.28 | TRUE | 9.38E-04 |
| BMI | rs6922607 | G | A | 0.190 | 0.0143 | 0.0025 | 1.50E-08 | 0.196 | -0.0040 | 0.0066 | 5.48E-01 | 454884 | 32.07 | TRUE | 1.01E-02 |
| BMI | rs6938973 | C | T | 0.602 | 0.0184 | 0.0020 | 1.90E-19 | 0.600 | 0.0043 | 0.0058 | 4.56E-01 | 454884 | 81.38 | TRUE | 2.97E-05 |
| BMI | rs6950388 | A | G | 0.795 | 0.0160 | 0.0025 | 9.20E-11 | 0.784 | -0.0030 | 0.0081 | 7.13E-01 | 454884 | 41.99 | TRUE | 2.26E-03 |
| BMI | rs6962980 | C | A | 0.556 | -0.0160 | 0.0020 | 1.60E-15 | 0.542 | 0.0018 | 0.0058 | 7.56E-01 | 454884 | 63.51 | TRUE | 1.08E-04 |
| BMI | rs698147 | G | A | 0.543 | -0.0133 | 0.0020 | 2.90E-11 | 0.520 | -0.0040 | 0.0052 | 4.39E-01 | 454884 | 44.23 | TRUE | 2.74E-03 |
| BMI | rs6998660 | G | A | 0.453 | 0.0119 | 0.0020 | 2.50E-09 | 0.436 | 0.0087 | 0.0056 | 1.21E-01 | 454884 | 35.51 | TRUE | 2.75E-02 |
| BMI | rs7024334 | G | T | 0.779 | -0.0135 | 0.0024 | 1.70E-08 | 0.766 | -0.0050 | 0.0074 | 5.02E-01 | 454884 | 31.79 | TRUE | 1.39E-02 |
| BMI | rs7027304 | T | C | 0.653 | 0.0148 | 0.0021 | 1.70E-12 | 0.656 | -0.0050 | 0.0061 | 4.17E-01 | 454884 | 49.75 | TRUE | 1.75E-03 |
| BMI | rs7034554 | G | A | 0.374 | -0.0135 | 0.0021 | 4.70E-11 | 0.361 | -0.0020 | 0.0049 | 6.86E-01 | 454884 | 43.29 | TRUE | 1.60E-03 |
| BMI | rs7038943 | C | T | 0.339 | -0.0142 | 0.0021 | 1.50E-11 | 0.358 | 0.0051 | 0.0058 | 3.76E-01 | 454884 | 45.59 | TRUE | 3.06E-03 |
| BMI | rs705145 | A | C | 0.345 | 0.0141 | 0.0021 | 1.60E-11 | 0.355 | -0.0060 | 0.0062 | 3.31E-01 | 454884 | 45.40 | TRUE | 4.02E-03 |
| BMI | rs7132908 | A | G | 0.384 | 0.0294 | 0.0020 | 1.10E-46 | 0.388 | 0.0028 | 0.0056 | 6.15E-01 | 454884 | 205.86 | TRUE | 2.27E-12 |
| BMI | rs7169847 | T | G | 0.636 | -0.0139 | 0.0021 | 2.00E-11 | 0.623 | 0.0014 | 0.0060 | 8.15E-01 | 454884 | 44.93 | TRUE | 1.07E-03 |
| BMI | rs7201895 | A | G | 0.354 | -0.0150 | 0.0021 | 8.00E-13 | 0.352 | 0.0054 | 0.0059 | 3.58E-01 | 454884 | 51.28 | TRUE | 1.67E-03 |
| BMI | rs7232171 | T | G | 0.583 | 0.0130 | 0.0020 | 1.20E-10 | 0.584 | 0.0039 | 0.0058 | 4.97E-01 | 454884 | 41.46 | TRUE | 3.71E-03 |
| BMI | rs723672 | T | C | 0.432 | 0.0111 | 0.0020 | 4.00E-08 | 0.429 | -0.0030 | 0.0054 | 5.76E-01 | 454884 | 30.17 | TRUE | 1.22E-02 |
| BMI | rs7244243 | A | C | 0.278 | 0.0137 | 0.0022 | 7.90E-10 | 0.293 | 0.0014 | 0.0062 | 8.21E-01 | 454884 | 37.79 | TRUE | 2.73E-03 |
| BMI | rs72617140 | C | A | 0.214 | 0.0187 | 0.0024 | 1.10E-14 | 0.220 | -0.0040 | 0.0073 | 5.86E-01 | 454884 | 59.63 | TRUE | 3.14E-04 |
| BMI | rs72634826 | A | G | 0.260 | -0.0207 | 0.0023 | 2.10E-19 | 0.247 | 0.0066 | 0.0066 | 3.18E-01 | 454884 | 81.15 | TRUE | 5.59E-05 |
| BMI | rs72673947 | G | A | 0.107 | 0.0222 | 0.0032 | 7.70E-12 | 0.115 | 0.0002 | 0.0111 | 9.86E-01 | 454884 | 46.83 | TRUE | 5.44E-04 |
| BMI | rs72739243 | C | T | 0.283 | -0.0136 | 0.0022 | 7.40E-10 | 0.278 | 0.0054 | 0.0062 | 3.81E-01 | 454884 | 37.92 | TRUE | 7.70E-03 |
| BMI | rs72910629 | G | A | 0.136 | 0.0163 | 0.0029 | 2.50E-08 | 0.128 | 0.0113 | 0.0086 | 1.91E-01 | 454884 | 31.03 | TRUE | 3.44E-02 |
| BMI | rs72948836 | G | A | 0.059 | -0.0245 | 0.0042 | 7.00E-09 | 0.061 | -0.0050 | 0.0124 | 6.87E-01 | 454884 | 33.55 | TRUE | 6.70E-03 |
| BMI | rs73026725 | A | C | 0.154 | -0.0223 | 0.0028 | 9.70E-16 | 0.151 | -0.0119 | 0.0079 | 1.33E-01 | 454884 | 64.50 | TRUE | 1.09E-03 |
| BMI | rs73052033 | C | T | 0.185 | -0.0300 | 0.0026 | 1.00E-31 | 0.174 | -0.0149 | 0.0076 | 5.15E-02 | 454884 | 137.28 | TRUE | 1.08E-06 |
| BMI | rs73142879 | T | C | 0.192 | -0.0272 | 0.0025 | 9.30E-27 | 0.183 | -0.0100 | 0.0071 | 1.62E-01 | 454884 | 114.67 | TRUE | 2.54E-06 |
| BMI | rs73193736 | G | A | 0.244 | -0.0184 | 0.0023 | 3.60E-15 | 0.223 | 0.0061 | 0.0068 | 3.69E-01 | 454884 | 61.92 | TRUE | 4.49E-04 |
| BMI | rs7331420 | A | G | 0.285 | -0.0142 | 0.0022 | 1.60E-10 | 0.290 | -0.0070 | 0.0067 | 2.95E-01 | 454884 | 40.85 | TRUE | 8.19E-03 |
| BMI | rs7357754 | G | A | 0.500 | 0.0144 | 0.0020 | 5.80E-13 | 0.495 | 0.0043 | 0.0056 | 4.42E-01 | 454884 | 51.91 | TRUE | 1.17E-03 |
| BMI | rs73601548 | T | C | 0.115 | 0.0171 | 0.0031 | 4.90E-08 | 0.119 | -0.0040 | 0.0092 | 6.63E-01 | 454884 | 29.76 | TRUE | 1.17E-02 |
| BMI | rs7442137 | T | C | 0.634 | -0.0125 | 0.0021 | 1.60E-09 | 0.620 | -0.0040 | 0.0057 | 4.80E-01 | 454884 | 36.44 | TRUE | 7.03E-03 |
| BMI | rs745249 | T | C | 0.282 | 0.0181 | 0.0022 | 3.30E-16 | 0.266 | 0.0054 | 0.0063 | 3.90E-01 | 454884 | 66.60 | TRUE | 2.27E-04 |
| BMI | rs7498044 | A | G | 0.217 | -0.0169 | 0.0024 | 4.70E-12 | 0.209 | -0.0020 | 0.0089 | 8.23E-01 | 454884 | 47.81 | TRUE | 8.00E-04 |
| BMI | rs7498665 | G | A | 0.400 | 0.0265 | 0.0020 | 9.30E-39 | 0.395 | 0.0080 | 0.0056 | 1.54E-01 | 454884 | 169.54 | TRUE | 4.58E-09 |
| BMI | rs75035127 | G | A | 0.030 | -0.0411 | 0.0058 | 1.20E-12 | 0.030 | 0.0119 | 0.0162 | 4.65E-01 | 454884 | 50.43 | TRUE | 1.29E-03 |
| BMI | rs7519259 | A | G | 0.528 | 0.0138 | 0.0020 | 5.80E-12 | 0.522 | 0.0072 | 0.0056 | 2.01E-01 | 454884 | 47.39 | TRUE | 4.95E-03 |
| BMI | rs75499503 | T | C | 0.220 | -0.0178 | 0.0024 | 3.10E-13 | 0.233 | 0.0016 | 0.0067 | 8.12E-01 | 454884 | 53.16 | TRUE | 3.58E-04 |
| BMI | rs75557510 | G | A | 0.061 | -0.0311 | 0.0042 | 1.60E-13 | 0.057 | -0.0030 | 0.0116 | 7.96E-01 | 454884 | 54.40 | TRUE | 3.08E-04 |
| BMI | rs76183894 | C | T | 0.081 | -0.0218 | 0.0037 | 2.70E-09 | 0.083 | 0.0108 | 0.0101 | 2.85E-01 | 454884 | 35.40 | TRUE | 1.40E-02 |
| BMI | rs7762794 | G | A | 0.286 | 0.0148 | 0.0022 | 2.00E-11 | 0.299 | -0.0060 | 0.0056 | 2.84E-01 | 454884 | 44.95 | TRUE | 3.94E-03 |
| BMI | rs7774 | A | C | 0.310 | 0.0153 | 0.0022 | 1.50E-12 | 0.324 | 0.0033 | 0.0062 | 5.92E-01 | 454884 | 50.07 | TRUE | 9.80E-04 |
| BMI | rs7805441 | T | C | 0.502 | 0.0134 | 0.0020 | 2.60E-11 | 0.515 | 0.0009 | 0.0058 | 8.76E-01 | 454884 | 44.49 | TRUE | 9.76E-04 |
| BMI | rs7828631 | T | C | 0.110 | 0.0182 | 0.0032 | 1.20E-08 | 0.109 | -0.0030 | 0.0102 | 7.69E-01 | 454884 | 32.47 | TRUE | 6.67E-03 |
| BMI | rs7852189 | G | A | 0.316 | 0.0172 | 0.0021 | 1.10E-15 | 0.323 | 0.0002 | 0.0048 | 9.67E-01 | 454884 | 64.25 | TRUE | 5.20E-05 |
| BMI | rs7893571 | T | G | 0.666 | 0.0144 | 0.0021 | 9.40E-12 | 0.664 | 0.0056 | 0.0060 | 3.52E-01 | 454884 | 46.46 | TRUE | 3.12E-03 |
| BMI | rs7916385 | T | C | 0.150 | -0.0241 | 0.0030 | 1.80E-15 | 0.149 | 0.0014 | 0.0091 | 8.78E-01 | 454884 | 63.25 | TRUE | 8.21E-05 |
| BMI | rs79212998 | G | T | 0.069 | -0.0234 | 0.0039 | 2.80E-09 | 0.065 | -0.0060 | 0.0121 | 6.22E-01 | 454884 | 35.29 | TRUE | 6.26E-03 |
| BMI | rs7924036 | T | G | 0.503 | -0.0145 | 0.0020 | 3.50E-13 | 0.498 | -0.0070 | 0.0055 | 2.07E-01 | 454884 | 52.89 | TRUE | 2.45E-03 |
| BMI | rs7947143 | A | G | 0.163 | -0.0178 | 0.0027 | 3.60E-11 | 0.157 | -0.0010 | 0.0060 | 8.67E-01 | 454884 | 43.79 | TRUE | 1.01E-03 |
| BMI | rs7952102 | C | T | 0.388 | -0.0143 | 0.0020 | 2.80E-12 | 0.384 | -0.0109 | 0.0059 | 6.53E-02 | 454884 | 48.81 | TRUE | 1.11E-02 |
| BMI | rs7975187 | G | A | 0.214 | 0.0155 | 0.0024 | 1.80E-10 | 0.206 | 0.0105 | 0.0068 | 1.26E-01 | 454884 | 40.64 | TRUE | 1.49E-02 |
| BMI | rs79780963 | T | C | 0.077 | 0.0236 | 0.0037 | 2.50E-10 | 0.817 | -0.0020 | 0.0115 | 8.62E-01 | 454884 | 40.02 | TRUE | 2.20E-03 |
| BMI | rs8015400 | A | C | 0.677 | 0.0214 | 0.0021 | 9.60E-24 | 0.675 | -0.0030 | 0.0058 | 6.08E-01 | 454884 | 100.91 | TRUE | 1.37E-06 |
| BMI | rs8076669 | C | T | 0.562 | 0.0138 | 0.0020 | 5.90E-12 | 0.546 | 0.0005 | 0.0062 | 9.35E-01 | 454884 | 47.37 | TRUE | 5.76E-04 |
| BMI | rs8112818 | G | A | 0.400 | -0.0203 | 0.0020 | 2.30E-23 | 0.394 | -0.0109 | 0.0060 | 6.64E-02 | 454884 | 99.15 | TRUE | 5.33E-05 |
| BMI | rs8132491 | A | G | 0.313 | -0.0155 | 0.0022 | 2.10E-12 | 0.310 | -0.0030 | 0.0071 | 6.73E-01 | 454884 | 49.40 | TRUE | 9.58E-04 |
| BMI | rs815163 | C | T | 0.563 | -0.0168 | 0.0020 | 4.60E-17 | 0.559 | 0.0044 | 0.0058 | 4.48E-01 | 454884 | 70.49 | TRUE | 1.21E-04 |
| BMI | rs862320 | T | C | 0.410 | -0.0232 | 0.0020 | 2.20E-30 | 0.406 | -0.0100 | 0.0057 | 7.98E-02 | 454884 | 131.23 | TRUE | 1.03E-06 |
| BMI | rs879620 | T | C | 0.613 | 0.0241 | 0.0021 | 5.70E-32 | 0.597 | 0.0037 | 0.0059 | 5.29E-01 | 454884 | 138.48 | TRUE | 1.87E-08 |
| BMI | rs909892 | A | G | 0.135 | -0.0180 | 0.0029 | 8.60E-10 | 0.127 | -0.0070 | 0.0089 | 4.32E-01 | 454884 | 37.61 | TRUE | 7.56E-03 |
| BMI | rs9291822 | T | C | 0.515 | -0.0145 | 0.0020 | 6.30E-13 | 0.502 | -0.0030 | 0.0056 | 5.93E-01 | 454884 | 51.76 | TRUE | 7.64E-04 |
| BMI | rs9294260 | A | G | 0.477 | 0.0147 | 0.0020 | 2.00E-13 | 0.468 | 0.0079 | 0.0057 | 1.63E-01 | 454884 | 53.96 | TRUE | 2.90E-03 |
| BMI | rs935166 | A | G | 0.507 | -0.0161 | 0.0020 | 5.90E-16 | 0.495 | -0.0109 | 0.0051 | 3.03E-02 | 454884 | 65.48 | TRUE | 2.13E-03 |
| BMI | rs947088 | T | G | 0.718 | 0.0128 | 0.0022 | 9.80E-09 | 0.712 | 0.0012 | 0.0063 | 8.50E-01 | 454884 | 32.88 | TRUE | 5.03E-03 |
| BMI | rs9478496 | C | T | 0.164 | 0.0176 | 0.0027 | 6.60E-11 | 0.168 | 0.0074 | 0.0076 | 3.29E-01 | 454884 | 42.63 | TRUE | 5.31E-03 |
| BMI | rs9571687 | A | C | 0.329 | -0.0132 | 0.0021 | 4.60E-10 | 0.332 | -0.0090 | 0.0061 | 1.40E-01 | 454884 | 38.86 | TRUE | 1.80E-02 |
| BMI | rs9638713 | G | A | 0.975 | -0.0357 | 0.0064 | 2.40E-08 | 0.957 | 0.0074 | 0.0182 | 6.83E-01 | 454884 | 31.15 | TRUE | 1.09E-02 |
| BMI | rs9673839 | G | A | 0.491 | 0.0130 | 0.0020 | 8.80E-11 | 0.484 | 0.0017 | 0.0058 | 7.71E-01 | 454884 | 42.07 | TRUE | 1.76E-03 |
| BMI | rs9843653 | C | T | 0.512 | 0.0292 | 0.0020 | 7.70E-49 | 0.506 | 0.0081 | 0.0056 | 1.46E-01 | 454884 | 215.73 | TRUE | 2.39E-11 |
| BMI | rs9888533 | T | C | 0.538 | 0.0122 | 0.0020 | 2.20E-09 | 0.506 | 0.0014 | 0.0059 | 8.12E-01 | 454884 | 35.77 | TRUE | 3.73E-03 |
| BMI | rs9944241 | C | T | 0.484 | -0.0146 | 0.0021 | 2.50E-12 | 0.495 | -0.0080 | 0.0062 | 1.97E-01 | 454884 | 49.05 | TRUE | 5.11E-03 |
| BMI | rs9951619 | G | T | 0.767 | 0.0150 | 0.0024 | 2.90E-10 | 0.752 | -0.0010 | 0.0103 | 9.23E-01 | 454884 | 39.75 | TRUE | 1.84E-03 |
| Diabetes | rs10184004 | T | C | 0.406 | -0.0033 | 0.0004 | 4.40E-15 | 0.394 | -0.0010 | 0.0092 | 9.13E-01 | 468298 | 62.31 | TRUE | 9.62E-01 |
| Diabetes | rs10228456 | T | C | 0.550 | 0.0032 | 0.0004 | 1.00E-14 | 0.535 | -0.0060 | 0.0059 | 3.14E-01 | 468298 | 59.35 | FALSE | 9.54E-01 |
| Diabetes | rs10420309 | G | A | 0.438 | -0.0031 | 0.0004 | 2.10E-13 | 0.427 | 0.0036 | 0.0056 | 5.21E-01 | 468298 | 54.12 | FALSE | 9.91E-01 |
| Diabetes | rs10811660 | A | G | 0.173 | -0.0063 | 0.0005 | 1.20E-31 | 0.171 | -0.0060 | 0.0079 | 4.48E-01 | 468298 | 135.51 | TRUE | 9.95E-01 |
| Diabetes | rs10974438 | C | A | 0.351 | 0.0025 | 0.0004 | 6.10E-09 | 0.347 | 0.0075 | 0.0059 | 2.06E-01 | 468298 | 34.10 | FALSE | 9.21E-01 |
| Diabetes | rs11257655 | T | C | 0.208 | 0.0038 | 0.0005 | 6.60E-14 | 0.211 | 0.0099 | 0.0078 | 2.03E-01 | 468298 | 57.13 | FALSE | 9.17E-01 |
| Diabetes | rs112674299 | T | C | 0.133 | -0.0036 | 0.0006 | 3.30E-09 | 0.134 | -0.0080 | 0.0080 | 3.21E-01 | 468298 | 34.36 | FALSE | 9.50E-01 |
| Diabetes | rs112845979 | T | C | 0.008 | -0.0129 | 0.0023 | 1.70E-08 | 0.011 | 0.0495 | 0.0394 | 2.09E-01 | 468298 | 31.29 | FALSE | 8.73E-01 |
| Diabetes | rs115505614 | T | C | 0.051 | 0.0077 | 0.0010 | 7.30E-16 | 0.044 | 0.0242 | 0.0122 | 4.70E-02 | 468298 | 66.01 | FALSE | 8.93E-01 |
| Diabetes | rs1169299 | C | T | 0.463 | 0.0023 | 0.0004 | 1.90E-08 | 0.459 | 0.0107 | 0.0050 | 3.24E-02 | 468298 | 31.67 | FALSE | 8.63E-01 |
| Diabetes | rs11720108 | T | C | 0.247 | -0.0038 | 0.0005 | 1.50E-15 | 0.229 | -0.0040 | 0.0072 | 5.81E-01 | 468298 | 62.73 | FALSE | 9.99E-01 |
| Diabetes | rs11759026 | G | A | 0.228 | 0.0034 | 0.0005 | 1.00E-11 | 0.229 | 0.0054 | 0.0069 | 4.35E-01 | 468298 | 47.06 | FALSE | 9.72E-01 |
| Diabetes | rs1260326 | C | T | 0.605 | 0.0034 | 0.0004 | 3.40E-15 | 0.587 | -0.0040 | 0.0057 | 4.80E-01 | 468298 | 63.82 | FALSE | 9.89E-01 |
| Diabetes | rs12910361 | G | A | 0.713 | 0.0031 | 0.0005 | 1.20E-11 | 0.698 | 0.0057 | 0.0062 | 3.55E-01 | 468298 | 45.86 | FALSE | 9.59E-01 |
| Diabetes | rs13262861 | A | C | 0.175 | -0.0044 | 0.0005 | 1.40E-15 | 0.183 | -0.0169 | 0.0066 | 1.02E-02 | 468298 | 62.90 | FALSE | 8.40E-01 |
| Diabetes | rs13414381 | C | T | 0.128 | -0.0051 | 0.0006 | 1.00E-16 | 0.123 | -0.0050 | 0.0079 | 5.26E-01 | 468298 | 69.11 | TRUE | 9.97E-01 |
| Diabetes | rs1421085 | C | T | 0.403 | 0.0047 | 0.0004 | 3.10E-29 | 0.408 | 0.0106 | 0.0049 | 3.22E-02 | 468298 | 125.71 | FALSE | 9.05E-01 |
| Diabetes | rs1496653 | G | A | 0.204 | -0.0034 | 0.0005 | 1.10E-11 | 0.199 | -0.0030 | 0.0078 | 7.01E-01 | 468298 | 45.31 | TRUE | 9.94E-01 |
| Diabetes | rs17036160 | T | C | 0.118 | -0.0044 | 0.0006 | 4.70E-12 | 0.119 | 0.0031 | 0.0085 | 7.13E-01 | 468298 | 46.13 | TRUE | 9.87E-01 |
| Diabetes | rs17513135 | T | C | 0.228 | 0.0032 | 0.0005 | 7.30E-11 | 0.213 | 0.0081 | 0.0070 | 2.47E-01 | 468298 | 43.42 | FALSE | 9.35E-01 |
| Diabetes | rs1800961 | T | C | 0.031 | 0.0080 | 0.0012 | 1.60E-11 | 0.031 | -0.0040 | 0.0182 | 8.27E-01 | 468298 | 45.47 | TRUE | 9.77E-01 |
| Diabetes | rs1801212 | A | G | 0.721 | 0.0040 | 0.0005 | 9.60E-19 | 0.724 | -0.0030 | 0.0066 | 6.50E-01 | 468298 | 75.78 | TRUE | 9.85E-01 |
| Diabetes | rs2074314 | T | C | 0.646 | -0.0030 | 0.0004 | 1.30E-12 | 0.629 | -0.0100 | 0.0060 | 9.63E-02 | 468298 | 49.52 | FALSE | 8.89E-01 |
| Diabetes | rs2237895 | C | A | 0.416 | 0.0041 | 0.0004 | 1.80E-23 | 0.418 | 0.0056 | 0.0056 | 3.15E-01 | 468298 | 98.64 | FALSE | 9.76E-01 |
| Diabetes | rs2278524 | A | G | 0.308 | 0.0025 | 0.0004 | 1.40E-08 | 0.289 | 0.0118 | 0.0063 | 6.08E-02 | 468298 | 31.41 | FALSE | 8.62E-01 |
| Diabetes | rs2303700 | C | T | 0.680 | -0.0027 | 0.0004 | 2.40E-09 | 0.632 | 0.0031 | 0.0063 | 6.24E-01 | 468298 | 36.46 | FALSE | 9.92E-01 |
| Diabetes | rs2793829 | T | C | 0.107 | 0.0037 | 0.0007 | 2.80E-08 | 0.103 | 0.0121 | 0.0091 | 1.85E-01 | 468298 | 31.32 | FALSE | 9.17E-01 |
| Diabetes | rs2796441 | A | G | 0.419 | -0.0034 | 0.0004 | 7.20E-16 | 0.408 | 0.0002 | 0.0062 | 9.74E-01 | 468298 | 64.86 | TRUE | 9.48E-01 |
| Diabetes | rs28746853 | C | T | 0.176 | 0.0042 | 0.0007 | 7.60E-10 | 0.171 | 0.0073 | 0.0083 | 3.79E-01 | 468298 | 38.16 | FALSE | 9.62E-01 |
| Diabetes | rs34715063 | C | T | 0.128 | 0.0045 | 0.0006 | 3.70E-13 | 0.128 | -0.0060 | 0.0086 | 4.88E-01 | 468298 | 53.81 | FALSE | 9.84E-01 |
| Diabetes | rs34744311 | T | C | 0.377 | -0.0046 | 0.0004 | 3.30E-27 | 0.371 | 0.0018 | 0.0058 | 7.55E-01 | 468298 | 115.86 | TRUE | 9.55E-01 |
| Diabetes | rs348330 | A | G | 0.633 | -0.0025 | 0.0004 | 7.00E-09 | 0.602 | -0.0040 | 0.0059 | 4.99E-01 | 468298 | 33.04 | FALSE | 9.75E-01 |
| Diabetes | rs35198068 | C | T | 0.291 | 0.0138 | 0.0005 | 1.00E-200 | 0.294 | 0.0179 | 0.0054 | 9.79E-04 | 468298 | 923.26 | FALSE | 9.38E-01 |
| Diabetes | rs3798526 | G | A | 0.180 | 0.0037 | 0.0005 | 1.80E-11 | 0.179 | 0.0172 | 0.0062 | 5.32E-03 | 468298 | 46.15 | FALSE | 8.29E-01 |
| Diabetes | rs3802177 | A | G | 0.310 | -0.0045 | 0.0004 | 3.40E-24 | 0.306 | -0.0109 | 0.0060 | 6.65E-02 | 468298 | 103.63 | FALSE | 9.03E-01 |
| Diabetes | rs4287436 | C | T | 0.619 | 0.0024 | 0.0004 | 5.60E-09 | 0.607 | 0.0043 | 0.0059 | 4.64E-01 | 468298 | 33.14 | FALSE | 9.70E-01 |
| Diabetes | rs4686471 | C | T | 0.610 | 0.0029 | 0.0004 | 6.20E-12 | 0.604 | 0.0079 | 0.0058 | 1.72E-01 | 468298 | 45.95 | FALSE | 9.18E-01 |
| Diabetes | rs6446298 | C | T | 0.666 | 0.0026 | 0.0004 | 2.40E-09 | 0.662 | 0.0141 | 0.0053 | 7.94E-03 | 468298 | 36.12 | FALSE | 8.21E-01 |
| Diabetes | rs7274168 | T | C | 0.479 | 0.0024 | 0.0004 | 2.10E-09 | 0.509 | -0.0010 | 0.0096 | 9.17E-01 | 468298 | 33.87 | TRUE | 9.77E-01 |
| Diabetes | rs7482891 | G | A | 0.622 | -0.0028 | 0.0004 | 5.90E-11 | 0.619 | -0.0139 | 0.0050 | 5.24E-03 | 468298 | 42.50 | FALSE | 8.22E-01 |
| Diabetes | rs76895963 | G | T | 0.021 | -0.0186 | 0.0016 | 1.90E-32 | 0.018 | -0.0383 | 0.0256 | 1.36E-01 | 468298 | 135.63 | FALSE | 9.21E-01 |
| Diabetes | rs7756992 | G | A | 0.266 | 0.0054 | 0.0005 | 1.00E-31 | 0.272 | 0.0136 | 0.0053 | 1.02E-02 | 468298 | 134.31 | FALSE | 8.79E-01 |
| Diabetes | rs7807512 | T | C | 0.297 | 0.0027 | 0.0005 | 5.40E-09 | 0.304 | -0.0070 | 0.0067 | 2.99E-01 | 468298 | 34.34 | FALSE | 9.34E-01 |
| Diabetes | rs7988007 | C | A | 0.175 | -0.0030 | 0.0005 | 3.10E-08 | 0.169 | 0.0004 | 0.0080 | 9.60E-01 | 468298 | 29.65 | TRUE | 9.68E-01 |
| Diabetes | rs8031576 | A | C | 0.719 | -0.0028 | 0.0005 | 3.30E-09 | 0.712 | -0.0109 | 0.0052 | 3.44E-02 | 468298 | 36.27 | FALSE | 8.77E-01 |
| Diabetes | rs860262 | A | C | 0.502 | -0.0036 | 0.0004 | 2.30E-18 | 0.495 | -0.0109 | 0.0045 | 1.61E-02 | 468298 | 77.00 | FALSE | 8.79E-01 |
| Diabetes | rs9379084 | A | G | 0.116 | -0.0053 | 0.0007 | 1.20E-15 | 0.133 | -0.0119 | 0.0085 | 1.58E-01 | 468298 | 62.69 | FALSE | 9.22E-01 |
| Diabetes | rs9686661 | T | C | 0.201 | 0.0034 | 0.0005 | 1.50E-11 | 0.196 | 0.0082 | 0.0071 | 2.44E-01 | 468298 | 44.35 | FALSE | 9.37E-01 |
| Diabetes | rs9808924 | A | G | 0.315 | 0.0048 | 0.0004 | 5.90E-28 | 0.313 | 0.0128 | 0.0052 | 1.44E-02 | 468298 | 117.03 | FALSE | 8.78E-01 |
| HDL | rs10138360 | A | G | 0.528 | -0.0158 | 0.0021 | 1.18E-14 | 0.539 | 0.0038 | 0.0057 | 5.01E-01 | 315133 | 56.61 | TRUE | 6.24E-05 |
| HDL | rs10149681 | A | G | 0.631 | -0.0139 | 0.0021 | 8.82E-11 | 0.657 | 0.0023 | 0.0059 | 6.97E-01 | 315133 | 43.81 | TRUE | 2.89E-04 |
| HDL | rs10233430 | C | T | 0.445 | -0.0204 | 0.0021 | 6.42E-23 | 0.420 | 0.0007 | 0.0054 | 8.98E-01 | 315133 | 94.37 | TRUE | 2.94E-08 |
| HDL | rs1045242 | G | A | 0.243 | 0.0161 | 0.0024 | 2.63E-11 | 0.273 | -0.0030 | 0.0072 | 6.78E-01 | 315133 | 45.00 | TRUE | 2.80E-04 |
| HDL | rs1047891 | A | C | 0.284 | -0.0182 | 0.0023 | 3.26E-15 | 0.308 | 0.0090 | 0.0070 | 1.98E-01 | 315133 | 62.62 | TRUE | 1.32E-04 |
| HDL | rs1054516 | C | T | 0.537 | -0.0141 | 0.0021 | 9.19E-12 | 0.525 | -0.0080 | 0.0059 | 1.75E-01 | 315133 | 45.08 | TRUE | 1.55E-03 |
| HDL | rs1054852 | G | A | 0.332 | 0.0336 | 0.0023 | 1.97E-47 | 0.369 | 0.0096 | 0.0061 | 1.12E-01 | 315133 | 213.41 | TRUE | 2.97E-14 |
| HDL | rs10761737 | C | T | 0.426 | 0.0147 | 0.0021 | 1.27E-12 | 0.411 | -0.0090 | 0.0057 | 1.18E-01 | 315133 | 49.00 | TRUE | 1.14E-03 |
| HDL | rs10786114 | T | C | 0.870 | 0.0216 | 0.0031 | 1.52E-12 | 0.861 | 0.0041 | 0.0085 | 6.27E-01 | 315133 | 48.55 | TRUE | 1.65E-04 |
| HDL | rs10798615 | G | T | 0.522 | -0.0190 | 0.0021 | 2.44E-20 | 0.517 | 0.0094 | 0.0055 | 8.76E-02 | 315133 | 81.86 | TRUE | 1.19E-05 |
| HDL | rs10886863 | C | T | 0.925 | -0.0314 | 0.0043 | 3.01E-13 | 0.965 | -0.0139 | 0.0191 | 4.67E-01 | 315133 | 53.32 | TRUE | 1.69E-04 |
| HDL | rs11009262 | T | G | 0.069 | -0.0243 | 0.0041 | 2.14E-09 | 0.054 | 0.0080 | 0.0132 | 5.44E-01 | 315133 | 35.13 | TRUE | 1.96E-03 |
| HDL | rs11021232 | C | T | 0.160 | -0.0171 | 0.0028 | 1.46E-09 | 0.184 | 0.0067 | 0.0073 | 3.55E-01 | 315133 | 37.30 | TRUE | 2.21E-03 |
| HDL | rs11039167 | T | C | 0.324 | 0.0449 | 0.0024 | 1.75E-76 | 0.312 | -0.0050 | 0.0065 | 4.46E-01 | 315133 | 350.00 | TRUE | 2.96E-25 |
| HDL | rs11065774 | A | G | 0.209 | -0.0961 | 0.0059 | 2.32E-60 | 0.071 | 0.0091 | 0.0115 | 4.27E-01 | 315133 | 265.30 | TRUE | 2.66E-19 |
| HDL | rs11075253 | A | C | 0.297 | 0.0244 | 0.0025 | 1.04E-22 | 0.292 | -0.0030 | 0.0055 | 5.84E-01 | 315133 | 95.26 | TRUE | 7.06E-08 |
| HDL | rs11075747 | A | G | 0.424 | -0.0120 | 0.0021 | 1.12E-08 | 0.380 | 0.0012 | 0.0056 | 8.30E-01 | 315133 | 32.65 | TRUE | 1.40E-03 |
| HDL | rs1107850 | A | G | 0.437 | -0.0164 | 0.0021 | 3.04E-15 | 0.474 | -0.0030 | 0.0063 | 6.35E-01 | 315133 | 60.99 | TRUE | 2.23E-05 |
| HDL | rs11178376 | A | G | 0.368 | 0.0135 | 0.0022 | 4.80E-10 | 0.327 | -0.0020 | 0.0059 | 7.37E-01 | 315133 | 37.65 | TRUE | 7.40E-04 |
| HDL | rs11254464 | C | T | 0.475 | 0.0126 | 0.0021 | 2.29E-09 | 0.427 | 0.0041 | 0.0057 | 4.68E-01 | 315133 | 36.00 | TRUE | 1.93E-03 |
| HDL | rs1132274 | A | C | 0.229 | -0.0192 | 0.0026 | 1.94E-13 | 0.149 | 0.0011 | 0.0076 | 8.84E-01 | 315133 | 54.53 | TRUE | 2.76E-05 |
| HDL | rs113234850 | G | T | 0.120 | -0.0225 | 0.0032 | 1.70E-12 | 0.097 | 0.0111 | 0.0094 | 2.40E-01 | 315133 | 49.44 | TRUE | 5.13E-04 |
| HDL | rs114443260 | T | C | 0.285 | -0.0246 | 0.0023 | 4.62E-27 | 0.251 | 0.0037 | 0.0067 | 5.82E-01 | 315133 | 114.40 | TRUE | 3.96E-09 |
| HDL | rs11635491 | A | G | 0.309 | 0.0819 | 0.0023 | 1.00E-200 | 0.250 | 0.0032 | 0.0069 | 6.41E-01 | 315133 | 1267.98 | TRUE | 1.54E-91 |
| HDL | rs11660745 | A | G | 0.146 | 0.0294 | 0.0029 | 6.10E-24 | 0.131 | 0.0028 | 0.0085 | 7.43E-01 | 315133 | 102.78 | TRUE | 1.33E-08 |
| HDL | rs116843064 | A | G | 0.020 | 0.2107 | 0.0082 | 1.94E-145 | 0.022 | -0.0411 | 0.0207 | 4.70E-02 | 315133 | 660.24 | TRUE | 5.21E-43 |
| HDL | rs11705639 | A | G | 0.167 | 0.0194 | 0.0031 | 1.98E-10 | 0.169 | 0.0033 | 0.0076 | 6.64E-01 | 315133 | 39.16 | TRUE | 6.97E-04 |
| HDL | rs117230571 | G | A | 0.077 | -0.0252 | 0.0043 | 6.09E-09 | 0.075 | 0.0037 | 0.0109 | 7.35E-01 | 315133 | 34.35 | TRUE | 1.34E-03 |
| HDL | rs11773763 | T | C | 0.347 | 0.0179 | 0.0022 | 1.24E-16 | 0.314 | -0.0060 | 0.0067 | 3.74E-01 | 315133 | 66.20 | TRUE | 2.67E-05 |
| HDL | rs11789603 | T | C | 0.098 | 0.0688 | 0.0035 | 2.10E-88 | 0.099 | -0.0090 | 0.0101 | 3.73E-01 | 315133 | 386.40 | TRUE | 1.71E-27 |
| HDL | rs12146566 | C | A | 0.206 | -0.0143 | 0.0025 | 1.88E-08 | 0.201 | -0.0010 | 0.0096 | 9.17E-01 | 315133 | 32.72 | TRUE | 1.24E-03 |
| HDL | rs1219550 | C | T | 0.195 | -0.0336 | 0.0026 | 4.59E-37 | 0.169 | -0.0109 | 0.0072 | 1.27E-01 | 315133 | 167.01 | TRUE | 1.76E-11 |
| HDL | rs12321904 | T | G | 0.457 | 0.0253 | 0.0021 | 2.87E-33 | 0.519 | 0.0076 | 0.0056 | 1.72E-01 | 315133 | 145.15 | TRUE | 3.56E-10 |
| HDL | rs12462109 | T | C | 0.313 | -0.0190 | 0.0022 | 1.78E-17 | 0.284 | 0.0028 | 0.0061 | 6.44E-01 | 315133 | 74.59 | TRUE | 1.95E-06 |
| HDL | rs12485478 | G | A | 0.065 | -0.0410 | 0.0044 | 1.53E-20 | 0.029 | 0.0288 | 0.0145 | 4.68E-02 | 315133 | 86.83 | TRUE | 6.41E-06 |
| HDL | rs1250604 | C | T | 0.722 | -0.0149 | 0.0024 | 3.96E-10 | 0.648 | 0.0098 | 0.0050 | 4.71E-02 | 315133 | 38.54 | TRUE | 6.09E-03 |
| HDL | rs12510382 | G | A | 0.462 | 0.0176 | 0.0023 | 1.91E-14 | 0.455 | 0.0034 | 0.0055 | 5.37E-01 | 315133 | 58.56 | TRUE | 3.90E-05 |
| HDL | rs12686780 | T | C | 0.232 | -0.0140 | 0.0025 | 2.96E-08 | 0.176 | 0.0056 | 0.0073 | 4.41E-01 | 315133 | 31.36 | TRUE | 4.36E-03 |
| HDL | rs12887521 | A | C | 0.635 | 0.0131 | 0.0021 | 9.00E-10 | 0.654 | -0.0040 | 0.0053 | 4.54E-01 | 315133 | 38.91 | TRUE | 1.11E-03 |
| HDL | rs12926107 | G | A | 0.385 | -0.0159 | 0.0022 | 7.58E-13 | 0.410 | 0.0007 | 0.0066 | 9.15E-01 | 315133 | 52.23 | TRUE | 3.96E-05 |
| HDL | rs13118477 | A | G | 0.439 | -0.0193 | 0.0021 | 9.51E-20 | 0.407 | 0.0004 | 0.0057 | 9.44E-01 | 315133 | 84.46 | TRUE | 1.37E-07 |
| HDL | rs1324472 | T | C | 0.651 | -0.0139 | 0.0022 | 2.12E-10 | 0.678 | 0.0067 | 0.0064 | 2.90E-01 | 315133 | 39.92 | TRUE | 2.03E-03 |
| HDL | rs13379043 | C | T | 0.282 | 0.0188 | 0.0023 | 3.78E-16 | 0.286 | 0.0022 | 0.0060 | 7.15E-01 | 315133 | 66.81 | TRUE | 5.63E-06 |
| HDL | rs138354839 | A | C | 0.015 | -0.0581 | 0.0099 | 4.72E-09 | 0.012 | -0.0040 | 0.0329 | 9.03E-01 | 315133 | 34.44 | TRUE | 9.29E-04 |
| HDL | rs138696161 | A | G | 0.016 | -0.1049 | 0.0093 | 1.12E-29 | 0.010 | -0.0383 | 0.0294 | 1.92E-01 | 315133 | 127.23 | TRUE | 5.98E-09 |
| HDL | rs139915535 | G | A | 0.018 | -0.1887 | 0.0086 | 6.90E-106 | 0.018 | 0.0218 | 0.0211 | 3.01E-01 | 315133 | 481.45 | TRUE | 5.99E-34 |
| HDL | rs140584594 | G | A | 0.731 | 0.0322 | 0.0025 | 1.02E-36 | 0.040 | -0.0257 | 0.0206 | 2.13E-01 | 315133 | 165.89 | TRUE | 6.45E-11 |
| HDL | rs141469619 | G | A | 0.010 | -0.2028 | 0.0120 | 1.05E-63 | 0.009 | -0.0050 | 0.0348 | 8.86E-01 | 315133 | 285.61 | TRUE | 4.13E-22 |
| HDL | rs1425604 | A | C | 0.545 | 0.0146 | 0.0022 | 1.27E-11 | 0.467 | -0.0100 | 0.0048 | 3.74E-02 | 315133 | 44.04 | TRUE | 3.25E-03 |
| HDL | rs145726796 | T | C | 0.022 | 0.0468 | 0.0080 | 4.53E-09 | 0.019 | 0.0010 | 0.0215 | 9.63E-01 | 315133 | 34.22 | TRUE | 8.01E-04 |
| HDL | rs1464454 | A | G | 0.386 | 0.0117 | 0.0021 | 3.48E-08 | 0.366 | -0.0020 | 0.0071 | 7.79E-01 | 315133 | 31.04 | TRUE | 2.34E-03 |
| HDL | rs147627829 | A | G | 0.044 | -0.0556 | 0.0056 | 1.72E-23 | 0.035 | 0.0187 | 0.0161 | 2.47E-01 | 315133 | 98.58 | TRUE | 3.03E-07 |
| HDL | rs147630132 | C | T | 0.024 | 0.0488 | 0.0077 | 2.09E-10 | 0.021 | 0.0138 | 0.0219 | 5.29E-01 | 315133 | 40.17 | TRUE | 9.61E-04 |
| HDL | rs150224153 | T | C | 0.030 | -0.0876 | 0.0068 | 8.50E-38 | 0.024 | 0.0001 | 0.0142 | 9.94E-01 | 315133 | 165.96 | TRUE | 1.07E-13 |
| HDL | rs150844304 | C | A | 0.025 | -0.0931 | 0.0073 | 7.98E-37 | 0.027 | 0.0265 | 0.0181 | 1.42E-01 | 315133 | 162.65 | TRUE | 4.46E-11 |
| HDL | rs151235402 | T | C | 0.016 | -0.0556 | 0.0093 | 1.83E-09 | 0.014 | 0.0286 | 0.0253 | 2.58E-01 | 315133 | 35.74 | TRUE | 4.23E-03 |
| HDL | rs1532625 | T | C | 0.417 | 0.1934 | 0.0021 | 1.00E-200 | 0.431 | -0.0070 | 0.0060 | 2.46E-01 | 315133 | 8481.53 | TRUE | 0.00E+00 |
| HDL | rs1534696 | A | C | 0.585 | 0.0193 | 0.0021 | 1.76E-19 | 0.547 | -0.0040 | 0.0053 | 4.53E-01 | 315133 | 84.46 | TRUE | 7.44E-07 |
| HDL | rs16928809 | A | G | 0.088 | -0.0256 | 0.0036 | 2.20E-12 | 0.095 | -0.0050 | 0.0100 | 6.18E-01 | 315133 | 50.57 | TRUE | 1.22E-04 |
| HDL | rs16965150 | T | C | 0.028 | 0.0567 | 0.0069 | 2.06E-16 | 0.027 | -0.0030 | 0.0153 | 8.45E-01 | 315133 | 67.53 | TRUE | 3.23E-06 |
| HDL | rs17041868 | C | T | 0.086 | -0.0223 | 0.0037 | 1.91E-09 | 0.063 | 0.0077 | 0.0114 | 4.97E-01 | 315133 | 36.33 | TRUE | 1.66E-03 |
| HDL | rs17326656 | T | G | 0.194 | -0.0200 | 0.0027 | 6.97E-14 | 0.222 | 0.0038 | 0.0068 | 5.75E-01 | 315133 | 54.87 | TRUE | 6.54E-05 |
| HDL | rs1734116 | G | A | 0.208 | -0.0183 | 0.0026 | 1.30E-12 | 0.175 | 0.0033 | 0.0075 | 6.60E-01 | 315133 | 49.54 | TRUE | 1.24E-04 |
| HDL | rs174567 | G | A | 0.351 | -0.0574 | 0.0024 | 2.52E-128 | 0.337 | 0.0077 | 0.0058 | 1.83E-01 | 315133 | 572.01 | TRUE | 1.76E-39 |
| HDL | rs17513135 | T | C | 0.214 | -0.0422 | 0.0025 | 1.40E-63 | 0.213 | 0.0081 | 0.0070 | 2.47E-01 | 315133 | 284.93 | TRUE | 5.86E-20 |
| HDL | rs17713879 | A | G | 0.335 | 0.0144 | 0.0022 | 5.43E-11 | 0.346 | -0.0050 | 0.0060 | 4.06E-01 | 315133 | 42.84 | TRUE | 7.97E-04 |
| HDL | rs185065940 | G | T | 0.056 | 0.0514 | 0.0052 | 4.84E-23 | 0.063 | 0.0207 | 0.0126 | 1.01E-01 | 315133 | 97.71 | TRUE | 1.51E-06 |
| HDL | rs1854962 | A | G | 0.346 | 0.0156 | 0.0023 | 1.19E-11 | 0.257 | 0.0028 | 0.0064 | 6.61E-01 | 315133 | 46.00 | TRUE | 2.19E-04 |
| HDL | rs186586197 | C | A | 0.032 | 0.0454 | 0.0067 | 1.38E-11 | 0.022 | 0.0079 | 0.0220 | 7.19E-01 | 315133 | 45.92 | TRUE | 2.12E-04 |
| HDL | rs1884622 | T | C | 0.561 | 0.0122 | 0.0021 | 6.14E-09 | 0.505 | 0.0026 | 0.0055 | 6.37E-01 | 315133 | 33.75 | TRUE | 1.81E-03 |
| HDL | rs193084249 | G | A | 0.024 | -0.0856 | 0.0076 | 2.64E-29 | 0.022 | 0.0093 | 0.0206 | 6.50E-01 | 315133 | 126.86 | TRUE | 3.94E-10 |
| HDL | rs2011614 | A | G | 0.293 | -0.0143 | 0.0023 | 9.11E-10 | 0.338 | -0.0020 | 0.0069 | 7.71E-01 | 315133 | 38.66 | TRUE | 6.27E-04 |
| HDL | rs201603078 | C | T | 0.536 | -0.0129 | 0.0023 | 1.72E-08 | 0.361 | 0.0084 | 0.0071 | 2.34E-01 | 315133 | 31.46 | TRUE | 1.19E-02 |
| HDL | rs2071570 | A | C | 0.272 | -0.0139 | 0.0024 | 3.59E-09 | 0.220 | 0.0112 | 0.0055 | 4.22E-02 | 315133 | 33.54 | TRUE | 1.18E-02 |
| HDL | rs2159502 | C | T | 0.405 | 0.0133 | 0.0021 | 3.10E-10 | 0.352 | 0.0086 | 0.0062 | 1.65E-01 | 315133 | 40.11 | TRUE | 3.51E-03 |
| HDL | rs2175766 | C | A | 0.472 | 0.0119 | 0.0021 | 2.86E-08 | 0.530 | 0.0021 | 0.0056 | 7.10E-01 | 315133 | 32.11 | TRUE | 2.05E-03 |
| HDL | rs2236999 | C | T | 0.176 | 0.0163 | 0.0028 | 4.50E-09 | 0.150 | 0.0077 | 0.0082 | 3.48E-01 | 315133 | 33.89 | TRUE | 4.14E-03 |
| HDL | rs2237035 | T | G | 0.341 | 0.0155 | 0.0022 | 2.11E-12 | 0.357 | 0.0020 | 0.0060 | 7.40E-01 | 315133 | 49.64 | TRUE | 9.82E-05 |
| HDL | rs2292318 | T | C | 0.124 | 0.0603 | 0.0031 | 1.25E-83 | 0.129 | 0.0105 | 0.0082 | 2.02E-01 | 315133 | 378.37 | TRUE | 2.97E-26 |
| HDL | rs2297402 | T | C | 0.033 | -0.0633 | 0.0060 | 3.16E-26 | 0.025 | 0.0125 | 0.0177 | 4.82E-01 | 315133 | 111.30 | TRUE | 9.32E-09 |
| HDL | rs2298214 | A | C | 0.618 | -0.0145 | 0.0022 | 2.27E-11 | 0.564 | 0.0081 | 0.0058 | 1.58E-01 | 315133 | 43.44 | TRUE | 2.05E-03 |
| HDL | rs2298632 | T | C | 0.517 | 0.0145 | 0.0021 | 3.77E-12 | 0.494 | 0.0023 | 0.0054 | 6.71E-01 | 315133 | 47.68 | TRUE | 1.57E-04 |
| HDL | rs2302263 | T | C | 0.167 | -0.0220 | 0.0031 | 6.61E-13 | 0.100 | -0.0010 | 0.0146 | 9.46E-01 | 315133 | 50.36 | TRUE | 5.29E-05 |
| HDL | rs2306363 | T | G | 0.220 | 0.0187 | 0.0025 | 4.25E-14 | 0.199 | -0.0119 | 0.0071 | 9.39E-02 | 315133 | 55.95 | TRUE | 5.29E-04 |
| HDL | rs2307111 | C | T | 0.426 | 0.0175 | 0.0021 | 5.40E-17 | 0.397 | -0.0109 | 0.0056 | 5.21E-02 | 315133 | 69.44 | TRUE | 1.17E-04 |
| HDL | rs233724 | A | G | 0.460 | 0.0163 | 0.0022 | 3.55E-13 | 0.505 | -0.0050 | 0.0058 | 3.90E-01 | 315133 | 54.89 | TRUE | 1.28E-04 |
| HDL | rs235314 | T | C | 0.508 | -0.0211 | 0.0021 | 1.49E-24 | 0.526 | -0.0040 | 0.0057 | 4.87E-01 | 315133 | 100.95 | TRUE | 5.24E-08 |
| HDL | rs2356683 | A | G | 0.491 | 0.0174 | 0.0023 | 2.50E-14 | 0.472 | -0.0030 | 0.0050 | 5.47E-01 | 315133 | 57.23 | TRUE | 4.15E-05 |
| HDL | rs2419605 | G | A | 0.168 | -0.0321 | 0.0028 | 4.92E-31 | 0.144 | -0.0109 | 0.0083 | 1.85E-01 | 315133 | 131.43 | TRUE | 3.02E-09 |
| HDL | rs2452904 | A | C | 0.319 | -0.0249 | 0.0022 | 1.21E-29 | 0.323 | -0.0050 | 0.0064 | 4.33E-01 | 315133 | 128.10 | TRUE | 9.63E-10 |
| HDL | rs2494748 | T | C | 0.551 | -0.0254 | 0.0022 | 1.87E-31 | 0.604 | 0.0021 | 0.0058 | 7.17E-01 | 315133 | 133.30 | TRUE | 9.19E-11 |
| HDL | rs254560 | A | G | 0.382 | -0.0128 | 0.0021 | 1.60E-09 | 0.401 | -0.0070 | 0.0057 | 2.22E-01 | 315133 | 37.15 | TRUE | 3.65E-03 |
| HDL | rs2575876 | A | G | 0.256 | -0.0737 | 0.0023 | 1.00E-200 | 0.255 | 0.0093 | 0.0066 | 1.55E-01 | 315133 | 1026.78 | TRUE | 1.50E-70 |
| HDL | rs2625390 | T | C | 0.637 | 0.0119 | 0.0021 | 3.06E-08 | 0.600 | 0.0020 | 0.0059 | 7.33E-01 | 315133 | 32.11 | TRUE | 1.97E-03 |
| HDL | rs2642438 | G | A | 0.725 | 0.0281 | 0.0023 | 3.56E-34 | 0.704 | -0.0030 | 0.0065 | 6.46E-01 | 315133 | 149.26 | TRUE | 9.97E-12 |
| HDL | rs2643194 | T | C | 0.657 | 0.0238 | 0.0022 | 1.21E-27 | 0.683 | -0.0060 | 0.0061 | 3.25E-01 | 315133 | 117.03 | TRUE | 9.12E-09 |
| HDL | rs2678379 | G | A | 0.693 | -0.0537 | 0.0025 | 1.12E-102 | 0.785 | 0.0030 | 0.0067 | 6.56E-01 | 315133 | 461.39 | TRUE | 4.26E-34 |
| HDL | rs2700908 | C | T | 0.343 | -0.0136 | 0.0022 | 7.10E-10 | 0.393 | -0.0070 | 0.0057 | 2.24E-01 | 315133 | 38.21 | TRUE | 3.08E-03 |
| HDL | rs2792751 | C | T | 0.737 | -0.0359 | 0.0023 | 1.26E-53 | 0.709 | 0.0112 | 0.0061 | 6.52E-02 | 315133 | 243.63 | TRUE | 5.42E-16 |
| HDL | rs2804894 | A | G | 0.725 | 0.0178 | 0.0023 | 1.36E-14 | 0.715 | 0.0038 | 0.0068 | 5.74E-01 | 315133 | 59.89 | TRUE | 3.19E-05 |
| HDL | rs2823293 | G | A | 0.213 | -0.0153 | 0.0026 | 2.11E-09 | 0.212 | 0.0143 | 0.0081 | 7.83E-02 | 315133 | 34.63 | TRUE | 1.86E-02 |
| HDL | rs28362901 | A | C | 0.146 | -0.0193 | 0.0031 | 4.13E-10 | 0.079 | -0.0070 | 0.0104 | 5.04E-01 | 315133 | 38.76 | TRUE | 1.13E-03 |
| HDL | rs28932178 | C | T | 0.209 | 0.0170 | 0.0027 | 2.22E-10 | 0.140 | -0.0080 | 0.0082 | 3.30E-01 | 315133 | 39.64 | TRUE | 1.69E-03 |
| HDL | rs2925979 | C | T | 0.692 | 0.0362 | 0.0022 | 7.03E-60 | 0.702 | -0.0060 | 0.0060 | 3.21E-01 | 315133 | 270.75 | TRUE | 2.12E-19 |
| HDL | rs2963472 | A | G | 0.221 | -0.0182 | 0.0025 | 2.60E-13 | 0.211 | 0.0051 | 0.0068 | 4.53E-01 | 315133 | 53.00 | TRUE | 1.29E-04 |
| HDL | rs2980888 | C | T | 0.699 | 0.0373 | 0.0022 | 1.56E-62 | 0.682 | 0.0047 | 0.0060 | 4.34E-01 | 315133 | 287.46 | TRUE | 5.87E-21 |
| HDL | rs325481 | A | G | 0.600 | 0.0134 | 0.0021 | 1.69E-10 | 0.580 | -0.0060 | 0.0060 | 3.20E-01 | 315133 | 40.72 | TRUE | 1.60E-03 |
| HDL | rs34055103 | A | G | 0.139 | 0.0204 | 0.0030 | 8.22E-12 | 0.130 | 0.0081 | 0.0086 | 3.44E-01 | 315133 | 46.24 | TRUE | 5.88E-04 |
| HDL | rs34138141 | T | G | 0.293 | -0.0199 | 0.0023 | 1.27E-18 | 0.256 | 0.0116 | 0.0065 | 7.45E-02 | 315133 | 74.86 | TRUE | 4.26E-05 |
| HDL | rs34724848 | A | G | 0.050 | 0.0532 | 0.0048 | 5.71E-29 | 0.034 | 0.0022 | 0.0156 | 8.88E-01 | 315133 | 122.84 | TRUE | 2.52E-10 |
| HDL | rs34940374 | A | G | 0.184 | -0.0165 | 0.0027 | 4.76E-10 | 0.187 | -0.0060 | 0.0072 | 4.04E-01 | 315133 | 37.35 | TRUE | 1.87E-03 |
| HDL | rs35628716 | T | G | 0.213 | 0.0300 | 0.0025 | 2.38E-32 | 0.175 | -0.0070 | 0.0071 | 3.25E-01 | 315133 | 144.00 | TRUE | 1.10E-10 |
| HDL | rs35777071 | C | T | 0.550 | 0.0155 | 0.0023 | 2.24E-11 | 0.635 | -0.0020 | 0.0054 | 7.13E-01 | 315133 | 45.42 | TRUE | 2.01E-04 |
| HDL | rs3732356 | T | G | 0.937 | -0.0288 | 0.0043 | 1.52E-11 | 0.923 | 0.0114 | 0.0115 | 3.25E-01 | 315133 | 44.86 | TRUE | 8.89E-04 |
| HDL | rs3746915 | G | A | 0.594 | 0.0116 | 0.0021 | 3.03E-08 | 0.569 | 0.0006 | 0.0061 | 9.22E-01 | 315133 | 30.51 | TRUE | 1.72E-03 |
| HDL | rs3775228 | T | C | 0.394 | -0.0207 | 0.0021 | 7.34E-23 | 0.390 | -0.0040 | 0.0057 | 4.87E-01 | 315133 | 97.16 | TRUE | 9.23E-08 |
| HDL | rs3803800 | G | A | 0.768 | 0.0223 | 0.0024 | 7.33E-20 | 0.787 | -0.0129 | 0.0069 | 6.23E-02 | 315133 | 86.34 | TRUE | 9.94E-06 |
| HDL | rs392794 | T | C | 0.694 | -0.0263 | 0.0024 | 2.32E-28 | 0.734 | -0.0100 | 0.0064 | 1.20E-01 | 315133 | 120.09 | TRUE | 3.03E-08 |
| HDL | rs4126104 | T | G | 0.248 | 0.1185 | 0.0024 | 1.00E-200 | 0.262 | -0.0030 | 0.0066 | 6.51E-01 | 315133 | 2437.89 | TRUE | 2.78E-175 |
| HDL | rs4149307 | T | C | 0.257 | 0.0615 | 0.0027 | 4.30E-116 | 0.159 | 0.0036 | 0.0081 | 6.55E-01 | 315133 | 518.83 | TRUE | 4.08E-38 |
| HDL | rs4239651 | C | T | 0.774 | 0.0222 | 0.0025 | 1.84E-19 | 0.805 | -0.0080 | 0.0069 | 2.46E-01 | 315133 | 78.85 | TRUE | 5.09E-06 |
| HDL | rs429358 | C | T | 0.146 | -0.0773 | 0.0029 | 1.59E-155 | 0.139 | -0.0070 | 0.0075 | 3.55E-01 | 315133 | 710.50 | TRUE | 1.62E-50 |
| HDL | rs4332553 | C | T | 0.411 | -0.0150 | 0.0023 | 1.11E-10 | 0.422 | 0.0003 | 0.0051 | 9.53E-01 | 315133 | 42.53 | TRUE | 1.86E-04 |
| HDL | rs4398423 | C | T | 0.712 | 0.0167 | 0.0023 | 1.34E-12 | 0.635 | 0.0008 | 0.0060 | 8.95E-01 | 315133 | 52.72 | TRUE | 3.76E-05 |
| HDL | rs4599108 | T | C | 0.563 | 0.0145 | 0.0022 | 8.03E-11 | 0.491 | 0.0026 | 0.0056 | 6.44E-01 | 315133 | 43.44 | TRUE | 3.55E-04 |
| HDL | rs4685262 | A | C | 0.661 | 0.0178 | 0.0024 | 3.22E-13 | 0.676 | -0.0030 | 0.0063 | 6.33E-01 | 315133 | 55.01 | TRUE | 5.57E-05 |
| HDL | rs4790310 | T | C | 0.514 | -0.0123 | 0.0021 | 7.58E-09 | 0.571 | 0.0110 | 0.0058 | 5.67E-02 | 315133 | 34.31 | TRUE | 1.59E-02 |
| HDL | rs4809221 | A | G | 0.624 | -0.0137 | 0.0022 | 2.79E-10 | 0.685 | 0.0093 | 0.0061 | 1.25E-01 | 315133 | 38.78 | TRUE | 4.82E-03 |
| HDL | rs4846913 | A | C | 0.527 | 0.0566 | 0.0022 | 1.53E-149 | 0.593 | 0.0026 | 0.0056 | 6.41E-01 | 315133 | 661.89 | TRUE | 2.23E-48 |
| HDL | rs493258 | C | T | 0.464 | -0.0746 | 0.0021 | 1.00E-200 | 0.537 | 0.0072 | 0.0055 | 1.87E-01 | 315133 | 1261.94 | TRUE | 1.19E-87 |
| HDL | rs4935801 | T | C | 0.830 | -0.0153 | 0.0027 | 2.36E-08 | 0.799 | -0.0080 | 0.0069 | 2.47E-01 | 315133 | 32.11 | TRUE | 6.94E-03 |
| HDL | rs541548517 | T | C | 0.011 | 0.0627 | 0.0115 | 4.60E-08 | 0.012 | -0.0020 | 0.0233 | 9.32E-01 | 315133 | 29.73 | TRUE | 1.89E-03 |
| HDL | rs553779 | G | A | 0.425 | 0.0215 | 0.0021 | 1.14E-23 | 0.479 | -0.0020 | 0.0047 | 6.68E-01 | 315133 | 104.82 | TRUE | 1.00E-08 |
| HDL | rs556107 | T | C | 0.575 | 0.0158 | 0.0021 | 1.09E-13 | 0.518 | 0.0079 | 0.0060 | 1.84E-01 | 315133 | 56.61 | TRUE | 2.79E-04 |
| HDL | rs55665473 | A | G | 0.203 | 0.0268 | 0.0026 | 3.97E-25 | 0.240 | 0.0049 | 0.0065 | 4.47E-01 | 315133 | 106.25 | TRUE | 2.48E-08 |
| HDL | rs55966152 | G | A | 0.163 | -0.0541 | 0.0028 | 7.55E-84 | 0.171 | 0.0052 | 0.0077 | 4.98E-01 | 315133 | 373.32 | TRUE | 3.46E-27 |
| HDL | rs56090699 | C | T | 0.283 | 0.0175 | 0.0023 | 2.75E-14 | 0.274 | 0.0023 | 0.0073 | 7.51E-01 | 315133 | 57.89 | TRUE | 2.58E-05 |
| HDL | rs59781045 | T | C | 0.061 | 0.0777 | 0.0043 | 9.39E-72 | 0.071 | 0.0143 | 0.0110 | 1.95E-01 | 315133 | 326.52 | TRUE | 1.49E-22 |
| HDL | rs59916403 | T | G | 0.409 | 0.0159 | 0.0021 | 1.18E-13 | 0.341 | 0.0023 | 0.0058 | 6.92E-01 | 315133 | 57.33 | TRUE | 2.98E-05 |
| HDL | rs6073958 | C | T | 0.181 | -0.0616 | 0.0027 | 1.54E-117 | 0.202 | 0.0122 | 0.0069 | 7.78E-02 | 315133 | 520.52 | TRUE | 1.00E-34 |
| HDL | rs61733623 | G | A | 0.018 | -0.0486 | 0.0086 | 1.29E-08 | 0.015 | 0.0398 | 0.0247 | 1.07E-01 | 315133 | 31.94 | TRUE | 1.65E-02 |
| HDL | rs61929761 | A | C | 0.510 | -0.0126 | 0.0022 | 4.55E-09 | 0.523 | -0.0050 | 0.0059 | 3.97E-01 | 315133 | 32.80 | TRUE | 4.23E-03 |
| HDL | rs62248901 | T | C | 0.184 | -0.0172 | 0.0027 | 1.15E-10 | 0.169 | 0.0054 | 0.0076 | 4.74E-01 | 315133 | 40.58 | TRUE | 9.19E-04 |
| HDL | rs62428831 | C | T | 0.141 | 0.0214 | 0.0033 | 9.71E-11 | 0.134 | 0.0020 | 0.0084 | 8.12E-01 | 315133 | 42.05 | TRUE | 2.93E-04 |
| HDL | rs62436601 | C | T | 0.373 | -0.0171 | 0.0021 | 1.07E-15 | 0.372 | -0.0040 | 0.0061 | 5.16E-01 | 315133 | 66.31 | TRUE | 1.33E-05 |
| HDL | rs62466318 | T | C | 0.186 | 0.0371 | 0.0027 | 2.84E-44 | 0.195 | -0.0129 | 0.0062 | 3.65E-02 | 315133 | 188.81 | TRUE | 2.30E-12 |
| HDL | rs62492368 | A | G | 0.365 | -0.0160 | 0.0022 | 5.86E-13 | 0.313 | -0.0010 | 0.0081 | 9.02E-01 | 315133 | 52.89 | TRUE | 3.88E-05 |
| HDL | rs62526883 | G | T | 0.702 | -0.0141 | 0.0023 | 2.03E-09 | 0.656 | -0.0030 | 0.0080 | 7.10E-01 | 315133 | 37.58 | TRUE | 1.02E-03 |
| HDL | rs632057 | G | T | 0.555 | 0.0192 | 0.0022 | 6.85E-19 | 0.628 | 0.0092 | 0.0057 | 1.06E-01 | 315133 | 76.17 | TRUE | 2.25E-05 |
| HDL | rs643186 | C | T | 0.789 | -0.0165 | 0.0026 | 1.05E-10 | 0.726 | 0.0019 | 0.0065 | 7.70E-01 | 315133 | 40.27 | TRUE | 4.46E-04 |
| HDL | rs6503091 | A | G | 0.529 | -0.0134 | 0.0022 | 8.02E-10 | 0.599 | 0.0054 | 0.0057 | 3.45E-01 | 315133 | 37.10 | TRUE | 2.36E-03 |
| HDL | rs6664374 | T | C | 0.437 | 0.0160 | 0.0022 | 8.63E-13 | 0.354 | -0.0080 | 0.0057 | 1.59E-01 | 315133 | 52.89 | TRUE | 4.37E-04 |
| HDL | rs6730325 | A | G | 0.602 | 0.0128 | 0.0023 | 4.37E-08 | 0.596 | -0.0060 | 0.0059 | 3.11E-01 | 315133 | 30.97 | TRUE | 7.19E-03 |
| HDL | rs6762415 | G | T | 0.566 | -0.0146 | 0.0021 | 3.22E-12 | 0.527 | -0.0030 | 0.0066 | 6.50E-01 | 315133 | 48.34 | TRUE | 1.80E-04 |
| HDL | rs6842517 | A | C | 0.443 | 0.0132 | 0.0023 | 1.96E-08 | 0.440 | -0.0010 | 0.0052 | 8.47E-01 | 315133 | 32.94 | TRUE | 1.26E-03 |
| HDL | rs6902116 | G | A | 0.304 | -0.0286 | 0.0023 | 9.75E-37 | 0.312 | -0.0010 | 0.0052 | 8.46E-01 | 315133 | 154.62 | TRUE | 1.30E-12 |
| HDL | rs690239 | T | C | 0.393 | 0.0124 | 0.0022 | 1.22E-08 | 0.366 | -0.0090 | 0.0065 | 1.71E-01 | 315133 | 31.77 | TRUE | 1.31E-02 |
| HDL | rs6932852 | T | C | 0.457 | 0.0171 | 0.0021 | 5.19E-16 | 0.410 | -0.0070 | 0.0057 | 2.19E-01 | 315133 | 66.31 | TRUE | 4.38E-05 |
| HDL | rs6974400 | A | G | 0.459 | 0.0290 | 0.0021 | 5.66E-43 | 0.477 | -0.0060 | 0.0063 | 3.42E-01 | 315133 | 190.70 | TRUE | 1.04E-13 |
| HDL | rs7036107 | G | A | 0.510 | -0.0130 | 0.0023 | 2.27E-08 | 0.500 | 0.0012 | 0.0056 | 8.31E-01 | 315133 | 31.95 | TRUE | 1.60E-03 |
| HDL | rs7070369 | A | G | 0.393 | -0.0125 | 0.0022 | 1.73E-08 | 0.323 | -0.0030 | 0.0054 | 5.82E-01 | 315133 | 32.28 | TRUE | 2.45E-03 |
| HDL | rs7111419 | G | A | 0.046 | 0.0351 | 0.0049 | 8.18E-13 | 0.050 | 0.0012 | 0.0128 | 9.25E-01 | 315133 | 51.31 | TRUE | 4.36E-05 |
| HDL | rs7136506 | C | T | 0.265 | -0.0352 | 0.0024 | 3.10E-48 | 0.219 | 0.0032 | 0.0071 | 6.54E-01 | 315133 | 215.11 | TRUE | 1.91E-16 |
| HDL | rs7170463 | G | A | 0.257 | 0.0205 | 0.0024 | 2.85E-17 | 0.303 | -0.0030 | 0.0075 | 6.89E-01 | 315133 | 72.96 | TRUE | 2.81E-06 |
| HDL | rs72647336 | A | G | 0.058 | -0.0408 | 0.0053 | 9.40E-15 | 0.053 | -0.0080 | 0.0141 | 5.72E-01 | 315133 | 59.26 | TRUE | 3.72E-05 |
| HDL | rs72801474 | A | G | 0.093 | 0.0247 | 0.0039 | 3.18E-10 | 0.084 | 0.0119 | 0.0099 | 2.30E-01 | 315133 | 40.11 | TRUE | 2.15E-03 |
| HDL | rs72836561 | T | C | 0.032 | -0.1703 | 0.0065 | 1.51E-152 | 0.031 | 0.0071 | 0.0157 | 6.50E-01 | 315133 | 686.44 | TRUE | 3.53E-50 |
| HDL | rs72959041 | A | G | 0.050 | -0.0462 | 0.0053 | 2.48E-18 | 0.048 | 0.0133 | 0.0132 | 3.14E-01 | 315133 | 75.99 | TRUE | 6.05E-06 |
| HDL | rs73243877 | G | A | 0.168 | -0.0250 | 0.0030 | 2.23E-16 | 0.172 | -0.0040 | 0.0077 | 6.05E-01 | 315133 | 69.44 | TRUE | 5.61E-06 |
| HDL | rs74025321 | G | A | 0.075 | -0.0277 | 0.0039 | 1.08E-12 | 0.069 | 0.0035 | 0.0109 | 7.48E-01 | 315133 | 50.45 | TRUE | 8.12E-05 |
| HDL | rs74328314 | G | A | 0.075 | 0.0259 | 0.0044 | 4.79E-09 | 0.064 | -0.0129 | 0.0116 | 2.67E-01 | 315133 | 34.65 | TRUE | 4.64E-03 |
| HDL | rs74444445 | C | T | 0.024 | -0.0772 | 0.0077 | 8.53E-24 | 0.022 | 0.0265 | 0.0207 | 1.99E-01 | 315133 | 100.52 | TRUE | 3.48E-07 |
| HDL | rs74543561 | G | A | 0.051 | 0.0321 | 0.0052 | 4.64E-10 | 0.050 | -0.0070 | 0.0141 | 6.20E-01 | 315133 | 38.11 | TRUE | 9.95E-04 |
| HDL | rs7516590 | G | A | 0.237 | -0.0163 | 0.0027 | 1.32E-09 | 0.218 | 0.0082 | 0.0067 | 2.21E-01 | 315133 | 36.45 | TRUE | 4.01E-03 |
| HDL | rs7522056 | A | G | 0.341 | -0.0130 | 0.0022 | 1.58E-09 | 0.315 | 0.0015 | 0.0060 | 8.02E-01 | 315133 | 34.92 | TRUE | 1.02E-03 |
| HDL | rs7622114 | A | C | 0.599 | 0.0133 | 0.0021 | 2.65E-10 | 0.575 | 0.0024 | 0.0057 | 6.71E-01 | 315133 | 40.11 | TRUE | 5.75E-04 |
| HDL | rs76428106 | C | T | 0.013 | -0.0598 | 0.0104 | 8.84E-09 | 0.015 | -0.0149 | 0.0277 | 5.90E-01 | 315133 | 33.06 | TRUE | 2.73E-03 |
| HDL | rs7730268 | G | T | 0.076 | -0.0266 | 0.0043 | 6.56E-10 | 0.074 | 0.0027 | 0.0110 | 8.07E-01 | 315133 | 38.27 | TRUE | 5.72E-04 |
| HDL | rs77609158 | T | C | 0.233 | -0.0156 | 0.0027 | 1.11E-08 | 0.227 | -0.0070 | 0.0074 | 3.48E-01 | 315133 | 33.38 | TRUE | 4.91E-03 |
| HDL | rs77960347 | G | A | 0.013 | 0.2777 | 0.0098 | 3.26E-175 | 0.013 | -0.0227 | 0.0242 | 3.48E-01 | 315133 | 802.97 | TRUE | 7.54E-57 |
| HDL | rs78132593 | A | C | 0.185 | 0.0219 | 0.0027 | 2.94E-16 | 0.205 | -0.0070 | 0.0073 | 3.37E-01 | 315133 | 65.79 | TRUE | 2.94E-05 |
| HDL | rs7817574 | C | T | 0.192 | 0.0329 | 0.0026 | 1.68E-36 | 0.185 | -0.0139 | 0.0064 | 2.99E-02 | 315133 | 160.12 | TRUE | 2.33E-10 |
| HDL | rs7824461 | G | A | 0.794 | -0.0169 | 0.0028 | 1.98E-09 | 0.781 | 0.0105 | 0.0074 | 1.58E-01 | 315133 | 36.43 | TRUE | 6.71E-03 |
| HDL | rs7844647 | C | T | 0.312 | 0.0147 | 0.0023 | 8.97E-11 | 0.259 | -0.0070 | 0.0068 | 3.06E-01 | 315133 | 40.85 | TRUE | 1.67E-03 |
| HDL | rs7964492 | C | A | 0.209 | 0.0311 | 0.0026 | 3.23E-34 | 0.231 | 0.0039 | 0.0065 | 5.48E-01 | 315133 | 143.08 | TRUE | 3.86E-11 |
| HDL | rs79719909 | G | A | 0.190 | 0.0299 | 0.0027 | 2.06E-29 | 0.200 | -0.0139 | 0.0074 | 5.92E-02 | 315133 | 122.64 | TRUE | 6.79E-08 |
| HDL | rs79949326 | T | C | 0.250 | 0.0253 | 0.0024 | 1.67E-26 | 0.246 | 0.0034 | 0.0066 | 6.08E-01 | 315133 | 111.13 | TRUE | 5.81E-09 |
| HDL | rs79953491 | G | A | 0.120 | 0.0402 | 0.0035 | 2.22E-30 | 0.122 | 0.0068 | 0.0088 | 4.39E-01 | 315133 | 131.92 | TRUE | 4.67E-10 |
| HDL | rs8037865 | C | T | 0.280 | -0.0185 | 0.0023 | 1.21E-15 | 0.262 | 0.0027 | 0.0066 | 6.81E-01 | 315133 | 64.70 | TRUE | 9.42E-06 |
| HDL | rs816020 | G | A | 0.622 | -0.0135 | 0.0023 | 1.02E-08 | 0.613 | -0.0010 | 0.0062 | 8.73E-01 | 315133 | 34.45 | TRUE | 9.64E-04 |
| HDL | rs830620 | T | C | 0.416 | 0.0135 | 0.0023 | 4.57E-09 | 0.409 | -0.0109 | 0.0058 | 5.93E-02 | 315133 | 34.45 | TRUE | 1.52E-02 |
| HDL | rs856534 | A | G | 0.439 | 0.0210 | 0.0021 | 3.10E-23 | 0.388 | 0.0039 | 0.0057 | 4.94E-01 | 315133 | 100.00 | TRUE | 5.59E-08 |
| HDL | rs900399 | G | A | 0.414 | 0.0171 | 0.0021 | 2.80E-16 | 0.384 | 0.0041 | 0.0066 | 5.36E-01 | 315133 | 66.31 | TRUE | 1.41E-05 |
| HDL | rs921919 | A | G | 0.612 | -0.0437 | 0.0022 | 1.95E-86 | 0.627 | 0.0027 | 0.0064 | 6.74E-01 | 315133 | 394.56 | TRUE | 2.94E-29 |
| HDL | rs9306 | C | T | 0.487 | -0.0133 | 0.0022 | 7.54E-10 | 0.396 | 0.0047 | 0.0064 | 4.59E-01 | 315133 | 36.55 | TRUE | 2.09E-03 |
| HDL | rs9604045 | T | G | 0.232 | 0.0163 | 0.0026 | 2.39E-10 | 0.275 | -0.0010 | 0.0057 | 8.60E-01 | 315133 | 39.30 | TRUE | 4.02E-04 |
| HDL | rs968050 | T | C | 0.460 | 0.0124 | 0.0021 | 1.87E-09 | 0.476 | 0.0091 | 0.0056 | 1.01E-01 | 315133 | 34.87 | TRUE | 9.53E-03 |
| HDL | rs9820930 | T | C | 0.780 | -0.0164 | 0.0026 | 1.73E-10 | 0.727 | 0.0134 | 0.0073 | 6.53E-02 | 315133 | 39.79 | TRUE | 1.00E-02 |
| HDL | rs9924686 | A | G | 0.457 | -0.0162 | 0.0023 | 1.49E-12 | 0.448 | -0.0080 | 0.0057 | 1.65E-01 | 315133 | 49.61 | TRUE | 7.87E-04 |
| HDL | rs9976784 | A | G | 0.182 | -0.0179 | 0.0027 | 1.81E-11 | 0.202 | 0.0016 | 0.0068 | 8.15E-01 | 315133 | 43.95 | TRUE | 2.06E-04 |
| HDL | rs998584 | A | C | 0.494 | -0.0324 | 0.0021 | 6.70E-56 | 0.477 | 0.0102 | 0.0056 | 7.17E-02 | 315133 | 238.04 | TRUE | 1.26E-15 |
| LDL | rs10080815 | G | T | 0.030 | 0.0343 | 0.0063 | 4.87E-08 | 0.020 | -0.0060 | 0.0226 | 7.91E-01 | 343621 | 29.64 | TRUE | 3.71E-03 |
| LDL | rs1010759 | A | G | 0.133 | -0.0250 | 0.0031 | 1.17E-15 | 0.148 | 0.0133 | 0.0078 | 8.90E-02 | 343621 | 65.04 | TRUE | 2.47E-04 |
| LDL | rs10201242 | A | G | 0.100 | -0.0226 | 0.0036 | 2.42E-10 | 0.072 | -0.0109 | 0.0112 | 3.30E-01 | 343621 | 39.41 | TRUE | 2.65E-03 |
| LDL | rs10423733 | C | T | 0.160 | -0.1038 | 0.0030 | 1.00E-200 | 0.180 | 0.0089 | 0.0073 | 2.21E-01 | 343621 | 1197.16 | TRUE | 1.56E-78 |
| LDL | rs10448340 | G | T | 0.303 | -0.0145 | 0.0023 | 3.92E-10 | 0.318 | -0.0030 | 0.0056 | 5.93E-01 | 343621 | 39.74 | TRUE | 1.03E-03 |
| LDL | rs10794579 | C | T | 0.573 | 0.0162 | 0.0021 | 3.65E-14 | 0.574 | 0.0059 | 0.0056 | 2.91E-01 | 343621 | 59.51 | TRUE | 1.48E-04 |
| LDL | rs1081105 | C | A | 0.028 | 0.1736 | 0.0072 | 3.79E-130 | 0.024 | -0.0315 | 0.0194 | 1.04E-01 | 343621 | 581.35 | TRUE | 1.26E-36 |
| LDL | rs10874711 | T | C | 0.688 | 0.0161 | 0.0024 | 1.26E-11 | 0.617 | -0.0109 | 0.0052 | 3.47E-02 | 343621 | 45.00 | TRUE | 5.16E-03 |
| LDL | rs10910476 | T | C | 0.505 | 0.0136 | 0.0022 | 6.04E-10 | 0.538 | -0.0020 | 0.0060 | 7.38E-01 | 343621 | 38.21 | TRUE | 1.02E-03 |
| LDL | rs10953298 | T | C | 0.212 | -0.0188 | 0.0026 | 9.57E-13 | 0.242 | 0.0115 | 0.0066 | 8.06E-02 | 343621 | 52.28 | TRUE | 1.56E-03 |
| LDL | rs11149612 | T | C | 0.512 | -0.0166 | 0.0022 | 4.33E-14 | 0.444 | 0.0028 | 0.0056 | 6.16E-01 | 343621 | 56.93 | TRUE | 6.98E-05 |
| LDL | rs112987086 | T | G | 0.723 | 0.0188 | 0.0027 | 1.67E-12 | 0.702 | -0.0100 | 0.0064 | 1.22E-01 | 343621 | 48.48 | TRUE | 2.08E-03 |
| LDL | rs113177823 | A | G | 0.054 | -0.0370 | 0.0053 | 1.98E-12 | 0.053 | 0.0189 | 0.0125 | 1.32E-01 | 343621 | 48.74 | TRUE | 1.65E-03 |
| LDL | rs113911544 | G | A | 0.040 | 0.0445 | 0.0054 | 1.52E-16 | 0.044 | 0.0155 | 0.0137 | 2.59E-01 | 343621 | 67.91 | TRUE | 5.29E-05 |
| LDL | rs115113608 | G | A | 0.017 | -0.0546 | 0.0094 | 6.76E-09 | 0.016 | 0.0059 | 0.0239 | 8.04E-01 | 343621 | 33.74 | TRUE | 1.81E-03 |
| LDL | rs11591147 | T | G | 0.018 | -0.3485 | 0.0089 | 1.00E-200 | 0.015 | -0.0030 | 0.0245 | 9.03E-01 | 343621 | 1533.29 | TRUE | 5.43E-106 |
| LDL | rs11601507 | A | C | 0.066 | 0.0335 | 0.0042 | 1.91E-15 | 0.071 | -0.0050 | 0.0111 | 6.55E-01 | 343621 | 63.62 | TRUE | 2.24E-05 |
| LDL | rs11621792 | T | C | 0.384 | 0.0185 | 0.0023 | 1.67E-15 | 0.449 | -0.0100 | 0.0057 | 7.98E-02 | 343621 | 64.70 | TRUE | 3.04E-04 |
| LDL | rs116734477 | T | C | 0.034 | -0.0514 | 0.0059 | 3.79E-18 | 0.036 | 0.0087 | 0.0153 | 5.69E-01 | 343621 | 75.90 | TRUE | 4.42E-06 |
| LDL | rs11709868 | T | G | 0.254 | -0.0159 | 0.0025 | 3.19E-10 | 0.294 | -0.0030 | 0.0070 | 6.68E-01 | 343621 | 40.45 | TRUE | 9.08E-04 |
| LDL | rs117139027 | A | G | 0.017 | -0.0598 | 0.0090 | 3.65E-11 | 0.013 | 0.0140 | 0.0325 | 6.67E-01 | 343621 | 44.15 | TRUE | 5.85E-04 |
| LDL | rs11789603 | T | C | 0.099 | 0.0210 | 0.0036 | 3.68E-09 | 0.099 | -0.0090 | 0.0101 | 3.73E-01 | 343621 | 34.03 | TRUE | 5.37E-03 |
| LDL | rs12208357 | T | C | 0.070 | 0.0569 | 0.0046 | 1.13E-34 | 0.075 | -0.0020 | 0.0091 | 8.26E-01 | 343621 | 153.01 | TRUE | 8.05E-12 |
| LDL | rs12445804 | A | G | 0.134 | 0.0249 | 0.0033 | 9.27E-14 | 0.064 | 0.0094 | 0.0126 | 4.55E-01 | 343621 | 56.93 | TRUE | 1.38E-04 |
| LDL | rs12539997 | C | T | 0.196 | -0.0152 | 0.0027 | 1.24E-08 | 0.182 | -0.0020 | 0.0062 | 7.48E-01 | 343621 | 31.69 | TRUE | 2.56E-03 |
| LDL | rs12588332 | C | T | 0.862 | 0.0189 | 0.0031 | 9.76E-10 | 0.847 | -0.0070 | 0.0074 | 3.47E-01 | 343621 | 37.17 | TRUE | 3.05E-03 |
| LDL | rs12905203 | G | A | 0.370 | -0.0123 | 0.0023 | 4.80E-08 | 0.393 | 0.0059 | 0.0059 | 3.12E-01 | 343621 | 28.60 | TRUE | 1.34E-02 |
| LDL | rs12916 | C | T | 0.422 | 0.0602 | 0.0021 | 5.46E-173 | 0.403 | -0.0060 | 0.0052 | 2.51E-01 | 343621 | 821.78 | TRUE | 2.16E-54 |
| LDL | rs13010586 | A | G | 0.311 | 0.0234 | 0.0024 | 4.76E-23 | 0.269 | 0.0059 | 0.0062 | 3.40E-01 | 343621 | 95.06 | TRUE | 6.04E-07 |
| LDL | rs13066351 | T | C | 0.082 | -0.0313 | 0.0043 | 2.82E-13 | 0.083 | -0.0090 | 0.0096 | 3.53E-01 | 343621 | 52.98 | TRUE | 2.82E-04 |
| LDL | rs13098031 | T | G | 0.223 | -0.0204 | 0.0026 | 1.36E-14 | 0.265 | 0.0079 | 0.0064 | 2.18E-01 | 343621 | 61.56 | TRUE | 1.67E-04 |
| LDL | rs13195522 | A | C | 0.120 | 0.0458 | 0.0036 | 1.04E-36 | 0.137 | -0.0030 | 0.0079 | 7.03E-01 | 343621 | 161.85 | TRUE | 3.81E-12 |
| LDL | rs140584594 | G | A | 0.731 | -0.0158 | 0.0026 | 2.31E-09 | 0.040 | -0.0257 | 0.0206 | 2.13E-01 | 343621 | 36.93 | TRUE | 1.27E-02 |
| LDL | rs141973904 | T | C | 0.010 | -0.0758 | 0.0119 | 1.97E-10 | 0.017 | -0.0198 | 0.0217 | 3.61E-01 | 343621 | 40.57 | TRUE | 1.88E-03 |
| LDL | rs144439590 | T | C | 0.030 | -0.1448 | 0.0070 | 1.11E-94 | 0.026 | 0.0166 | 0.0184 | 3.66E-01 | 343621 | 427.90 | TRUE | 1.12E-28 |
| LDL | rs145730801 | C | T | 0.045 | 0.0344 | 0.0058 | 2.37E-09 | 0.044 | -0.0149 | 0.0150 | 3.20E-01 | 343621 | 35.18 | TRUE | 5.59E-03 |
| LDL | rs146534110 | T | G | 0.013 | 0.0627 | 0.0103 | 9.93E-10 | 0.014 | -0.0305 | 0.0260 | 2.40E-01 | 343621 | 37.06 | TRUE | 5.61E-03 |
| LDL | rs147711004 | A | G | 0.037 | 0.1486 | 0.0064 | 3.62E-120 | 0.036 | -0.0129 | 0.0179 | 4.71E-01 | 343621 | 539.11 | TRUE | 2.79E-36 |
| LDL | rs17050272 | A | G | 0.422 | -0.0207 | 0.0021 | 3.90E-22 | 0.418 | 0.0010 | 0.0058 | 8.62E-01 | 343621 | 97.16 | TRUE | 5.46E-08 |
| LDL | rs174564 | G | A | 0.357 | -0.0305 | 0.0022 | 1.71E-43 | 0.336 | 0.0072 | 0.0058 | 2.15E-01 | 343621 | 192.20 | TRUE | 8.56E-13 |
| LDL | rs1800961 | T | C | 0.028 | -0.0634 | 0.0065 | 1.57E-22 | 0.031 | -0.0040 | 0.0182 | 8.27E-01 | 343621 | 95.14 | TRUE | 9.16E-08 |
| LDL | rs2066714 | C | T | 0.216 | 0.0197 | 0.0029 | 7.96E-12 | 0.129 | 0.0012 | 0.0081 | 8.82E-01 | 343621 | 46.15 | TRUE | 1.88E-04 |
| LDL | rs2068888 | A | G | 0.479 | -0.0185 | 0.0021 | 3.95E-18 | 0.458 | 0.0046 | 0.0055 | 4.00E-01 | 343621 | 77.61 | TRUE | 6.13E-06 |
| LDL | rs2160994 | C | T | 0.704 | 0.0180 | 0.0024 | 1.36E-13 | 0.645 | 0.0066 | 0.0060 | 2.69E-01 | 343621 | 56.25 | TRUE | 2.84E-04 |
| LDL | rs2253736 | A | C | 0.877 | -0.0197 | 0.0033 | 2.65E-09 | 0.902 | -0.0030 | 0.0093 | 7.46E-01 | 343621 | 35.64 | TRUE | 1.45E-03 |
| LDL | rs2282889 | G | A | 0.553 | 0.0141 | 0.0022 | 7.33E-11 | 0.576 | -0.0070 | 0.0057 | 2.25E-01 | 343621 | 41.08 | TRUE | 3.00E-03 |
| LDL | rs2297367 | T | C | 0.040 | -0.0306 | 0.0054 | 1.55E-08 | 0.042 | -0.0109 | 0.0147 | 4.57E-01 | 343621 | 32.11 | TRUE | 5.46E-03 |
| LDL | rs2302429 | A | G | 0.207 | 0.0149 | 0.0026 | 1.15E-08 | 0.180 | -0.0070 | 0.0073 | 3.38E-01 | 343621 | 32.84 | TRUE | 6.40E-03 |
| LDL | rs2306363 | T | G | 0.219 | 0.0146 | 0.0026 | 1.09E-08 | 0.199 | -0.0119 | 0.0071 | 9.39E-02 | 343621 | 31.53 | TRUE | 2.24E-02 |
| LDL | rs2414578 | C | T | 0.472 | 0.0158 | 0.0023 | 5.61E-12 | 0.376 | -0.0090 | 0.0056 | 1.07E-01 | 343621 | 47.19 | TRUE | 2.18E-03 |
| LDL | rs2618566 | T | G | 0.665 | -0.0219 | 0.0023 | 2.48E-22 | 0.640 | 0.0104 | 0.0052 | 4.48E-02 | 343621 | 90.66 | TRUE | 9.14E-06 |
| LDL | rs2642438 | G | A | 0.723 | 0.0248 | 0.0024 | 1.73E-25 | 0.704 | -0.0030 | 0.0065 | 6.46E-01 | 343621 | 106.78 | TRUE | 2.95E-08 |
| LDL | rs2706383 | A | G | 0.217 | -0.0162 | 0.0026 | 3.54E-10 | 0.201 | -0.0060 | 0.0067 | 3.72E-01 | 343621 | 38.82 | TRUE | 2.23E-03 |
| LDL | rs2710644 | C | A | 0.712 | 0.0212 | 0.0023 | 1.45E-19 | 0.672 | -0.0060 | 0.0063 | 3.46E-01 | 343621 | 84.96 | TRUE | 3.20E-06 |
| LDL | rs2737245 | T | G | 0.279 | -0.0229 | 0.0024 | 3.36E-22 | 0.263 | 0.0125 | 0.0057 | 2.85E-02 | 343621 | 91.04 | TRUE | 1.34E-05 |
| LDL | rs2740488 | C | A | 0.267 | -0.0213 | 0.0024 | 4.44E-19 | 0.265 | 0.0057 | 0.0064 | 3.71E-01 | 343621 | 78.77 | TRUE | 6.26E-06 |
| LDL | rs28615248 | C | T | 0.199 | 0.0230 | 0.0027 | 5.36E-18 | 0.182 | 0.0074 | 0.0072 | 3.05E-01 | 343621 | 72.57 | TRUE | 2.08E-05 |
| LDL | rs29267 | A | G | 0.136 | 0.0204 | 0.0031 | 3.39E-11 | 0.126 | -0.0100 | 0.0091 | 2.74E-01 | 343621 | 43.30 | TRUE | 2.00E-03 |
| LDL | rs2928629 | C | T | 0.671 | 0.0125 | 0.0023 | 3.45E-08 | 0.658 | 0.0052 | 0.0060 | 3.83E-01 | 343621 | 29.54 | TRUE | 9.37E-03 |
| LDL | rs334558 | G | A | 0.391 | 0.0155 | 0.0022 | 4.62E-12 | 0.354 | -0.0090 | 0.0063 | 1.55E-01 | 343621 | 49.64 | TRUE | 1.51E-03 |
| LDL | rs34751842 | C | A | 0.059 | -0.0247 | 0.0045 | 4.56E-08 | 0.040 | -0.0050 | 0.0152 | 7.43E-01 | 343621 | 30.13 | TRUE | 3.72E-03 |
| LDL | rs35081008 | T | C | 0.147 | -0.0349 | 0.0033 | 1.70E-25 | 0.150 | 0.0117 | 0.0080 | 1.47E-01 | 343621 | 111.85 | TRUE | 2.31E-07 |
| LDL | rs35203651 | C | T | 0.114 | 0.0191 | 0.0033 | 1.11E-08 | 0.103 | 0.0026 | 0.0092 | 7.77E-01 | 343621 | 33.50 | TRUE | 1.93E-03 |
| LDL | rs35206901 | G | A | 0.182 | -0.0384 | 0.0030 | 2.55E-36 | 0.184 | -0.0070 | 0.0075 | 3.52E-01 | 343621 | 163.84 | TRUE | 2.32E-11 |
| LDL | rs36043200 | A | G | 0.539 | -0.0227 | 0.0021 | 4.22E-26 | 0.499 | 0.0051 | 0.0059 | 3.83E-01 | 343621 | 116.85 | TRUE | 2.20E-08 |
| LDL | rs3738621 | G | A | 0.219 | -0.0162 | 0.0026 | 2.29E-10 | 0.211 | 0.0030 | 0.0067 | 6.55E-01 | 343621 | 38.82 | TRUE | 1.07E-03 |
| LDL | rs3741298 | T | C | 0.746 | -0.0336 | 0.0026 | 2.12E-39 | 0.792 | -0.0100 | 0.0069 | 1.48E-01 | 343621 | 167.01 | TRUE | 7.39E-11 |
| LDL | rs3756772 | T | C | 0.453 | 0.0170 | 0.0022 | 7.18E-15 | 0.418 | -0.0060 | 0.0056 | 2.88E-01 | 343621 | 59.71 | TRUE | 1.47E-04 |
| LDL | rs3780181 | G | A | 0.071 | -0.0298 | 0.0041 | 4.67E-13 | 0.067 | 0.0211 | 0.0114 | 6.29E-02 | 343621 | 52.83 | TRUE | 1.83E-03 |
| LDL | rs3794695 | T | C | 0.224 | 0.0464 | 0.0026 | 7.53E-73 | 0.200 | 0.0006 | 0.0072 | 9.33E-01 | 343621 | 318.49 | TRUE | 2.32E-23 |
| LDL | rs4044515 | A | G | 0.053 | -0.0282 | 0.0050 | 1.61E-08 | 0.018 | 0.0215 | 0.0254 | 3.96E-01 | 343621 | 31.81 | TRUE | 8.20E-03 |
| LDL | rs41280378 | G | T | 0.079 | -0.0315 | 0.0041 | 1.61E-14 | 0.044 | 0.0218 | 0.0137 | 1.11E-01 | 343621 | 59.03 | TRUE | 4.67E-04 |
| LDL | rs4302748 | A | G | 0.176 | 0.0165 | 0.0028 | 3.10E-09 | 0.196 | 0.0094 | 0.0070 | 1.76E-01 | 343621 | 34.73 | TRUE | 8.67E-03 |
| LDL | rs440677 | A | G | 0.575 | -0.0171 | 0.0022 | 7.86E-15 | 0.611 | -0.0060 | 0.0059 | 3.13E-01 | 343621 | 60.42 | TRUE | 1.29E-04 |
| LDL | rs4678192 | G | A | 0.409 | 0.0137 | 0.0022 | 2.29E-10 | 0.388 | 0.0028 | 0.0058 | 6.30E-01 | 343621 | 38.78 | TRUE | 1.18E-03 |
| LDL | rs472495 | T | G | 0.672 | 0.0438 | 0.0023 | 2.56E-83 | 0.639 | -0.0050 | 0.0054 | 3.57E-01 | 343621 | 362.65 | TRUE | 1.33E-24 |
| LDL | rs4738684 | G | A | 0.695 | -0.0294 | 0.0023 | 1.48E-36 | 0.645 | -0.0020 | 0.0078 | 7.98E-01 | 343621 | 163.40 | TRUE | 2.72E-12 |
| LDL | rs4804147 | G | A | 0.413 | 0.0273 | 0.0022 | 2.94E-34 | 0.458 | -0.0020 | 0.0052 | 6.99E-01 | 343621 | 153.99 | TRUE | 1.25E-11 |
| LDL | rs4939883 | C | T | 0.814 | 0.0157 | 0.0027 | 6.90E-09 | 0.830 | 0.0061 | 0.0073 | 4.01E-01 | 343621 | 33.81 | TRUE | 4.47E-03 |
| LDL | rs4970834 | T | C | 0.165 | -0.1054 | 0.0029 | 1.00E-200 | 0.180 | 0.0043 | 0.0073 | 5.55E-01 | 343621 | 1320.95 | TRUE | 1.66E-89 |
| LDL | rs553427 | T | C | 0.551 | 0.0353 | 0.0022 | 1.70E-60 | 0.515 | 0.0079 | 0.0059 | 1.82E-01 | 343621 | 257.46 | TRUE | 1.29E-16 |
| LDL | rs55714927 | T | C | 0.209 | -0.0258 | 0.0027 | 4.96E-22 | 0.180 | -0.0060 | 0.0076 | 4.34E-01 | 343621 | 91.31 | TRUE | 7.83E-07 |
| LDL | rs55921103 | T | G | 0.632 | 0.0129 | 0.0023 | 1.02E-08 | 0.629 | 0.0022 | 0.0062 | 7.23E-01 | 343621 | 31.46 | TRUE | 3.17E-03 |
| LDL | rs56236159 | G | T | 0.233 | 0.0183 | 0.0029 | 3.36E-10 | 0.138 | -0.0030 | 0.0091 | 7.43E-01 | 343621 | 39.82 | TRUE | 8.08E-04 |
| LDL | rs5758128 | T | C | 0.474 | 0.0136 | 0.0024 | 8.84E-09 | 0.469 | -0.0030 | 0.0059 | 6.14E-01 | 343621 | 32.11 | TRUE | 3.67E-03 |
| LDL | rs58198139 | T | C | 0.670 | 0.0345 | 0.0023 | 1.41E-51 | 0.624 | -0.0070 | 0.0053 | 1.92E-01 | 343621 | 225.00 | TRUE | 6.44E-15 |
| LDL | rs59784135 | G | A | 0.640 | -0.0268 | 0.0024 | 4.30E-30 | 0.680 | 0.0056 | 0.0065 | 3.88E-01 | 343621 | 124.69 | TRUE | 7.29E-09 |
| LDL | rs60309576 | A | G | 0.222 | 0.0152 | 0.0026 | 8.42E-09 | 0.254 | 0.0074 | 0.0065 | 2.57E-01 | 343621 | 34.18 | TRUE | 7.12E-03 |
| LDL | rs6129717 | G | A | 0.292 | 0.0132 | 0.0023 | 1.44E-08 | 0.291 | -0.0020 | 0.0074 | 7.86E-01 | 343621 | 32.94 | TRUE | 2.27E-03 |
| LDL | rs61775192 | G | A | 0.482 | -0.0241 | 0.0022 | 1.90E-28 | 0.449 | 0.0107 | 0.0049 | 3.01E-02 | 343621 | 120.00 | TRUE | 2.22E-07 |
| LDL | rs62118464 | A | G | 0.118 | 0.0316 | 0.0037 | 2.78E-17 | 0.085 | -0.0109 | 0.0119 | 3.59E-01 | 343621 | 72.94 | TRUE | 2.29E-05 |
| LDL | rs62131701 | G | A | 0.074 | 0.0343 | 0.0045 | 2.70E-14 | 0.052 | -0.0080 | 0.0151 | 5.97E-01 | 343621 | 58.10 | TRUE | 7.74E-05 |
| LDL | rs62275881 | C | A | 0.260 | 0.0200 | 0.0027 | 1.04E-13 | 0.272 | -0.0010 | 0.0081 | 9.02E-01 | 343621 | 54.87 | TRUE | 4.67E-05 |
| LDL | rs6475606 | T | C | 0.516 | -0.0195 | 0.0021 | 1.03E-19 | 0.487 | 0.0096 | 0.0048 | 4.41E-02 | 343621 | 86.22 | TRUE | 1.48E-05 |
| LDL | rs6602911 | T | C | 0.372 | 0.0203 | 0.0022 | 2.53E-20 | 0.351 | -0.0020 | 0.0064 | 7.53E-01 | 343621 | 85.14 | TRUE | 5.76E-07 |
| LDL | rs6680227 | A | G | 0.035 | -0.0774 | 0.0064 | 1.63E-33 | 0.035 | -0.0178 | 0.0155 | 2.50E-01 | 343621 | 146.26 | TRUE | 6.20E-10 |
| LDL | rs6920309 | T | C | 0.545 | -0.0215 | 0.0023 | 4.28E-20 | 0.461 | 0.0084 | 0.0056 | 1.35E-01 | 343621 | 87.38 | TRUE | 7.54E-06 |
| LDL | rs704 | A | G | 0.526 | 0.0182 | 0.0022 | 5.29E-17 | 0.478 | 0.0019 | 0.0056 | 7.34E-01 | 343621 | 68.44 | TRUE | 8.01E-06 |
| LDL | rs7096937 | C | T | 0.739 | -0.0210 | 0.0024 | 5.87E-18 | 0.702 | 0.0093 | 0.0066 | 1.58E-01 | 343621 | 76.56 | TRUE | 3.63E-05 |
| LDL | rs7275804 | C | T | 0.323 | -0.0125 | 0.0023 | 3.83E-08 | 0.324 | -0.0010 | 0.0041 | 8.06E-01 | 343621 | 29.54 | TRUE | 3.06E-03 |
| LDL | rs73034893 | T | C | 0.069 | 0.0273 | 0.0047 | 5.46E-09 | 0.066 | -0.0010 | 0.0136 | 9.41E-01 | 343621 | 33.74 | TRUE | 1.32E-03 |
| LDL | rs73066466 | C | A | 0.251 | 0.0280 | 0.0024 | 2.09E-30 | 0.213 | -0.0030 | 0.0066 | 6.52E-01 | 343621 | 136.11 | TRUE | 2.67E-10 |
| LDL | rs7412 | T | C | 0.073 | -0.4770 | 0.0040 | 1.00E-200 | 0.081 | 0.0296 | 0.0090 | 9.46E-04 | 343621 | 14220.56 | TRUE | 0.00E+00 |
| LDL | rs75331444 | A | G | 0.055 | -0.0978 | 0.0047 | 8.96E-96 | 0.067 | 0.0015 | 0.0108 | 8.89E-01 | 343621 | 432.99 | TRUE | 4.44E-31 |
| LDL | rs75460349 | C | A | 0.024 | 0.0580 | 0.0079 | 1.58E-13 | 0.023 | 0.0056 | 0.0185 | 7.62E-01 | 343621 | 53.90 | TRUE | 7.42E-05 |
| LDL | rs7569317 | C | T | 0.571 | 0.0178 | 0.0022 | 1.87E-16 | 0.513 | -0.0020 | 0.0066 | 7.61E-01 | 343621 | 65.46 | TRUE | 1.34E-05 |
| LDL | rs7601939 | T | G | 0.177 | 0.0188 | 0.0028 | 1.77E-11 | 0.183 | -0.0050 | 0.0079 | 5.26E-01 | 343621 | 45.08 | TRUE | 6.39E-04 |
| LDL | rs76127343 | T | C | 0.042 | 0.0330 | 0.0059 | 1.88E-08 | 0.038 | -0.0109 | 0.0161 | 4.96E-01 | 343621 | 31.28 | TRUE | 5.85E-03 |
| LDL | rs7730594 | G | A | 0.549 | 0.0212 | 0.0024 | 2.61E-19 | 0.547 | -0.0020 | 0.0066 | 7.62E-01 | 343621 | 78.03 | TRUE | 1.83E-06 |
| LDL | rs77542162 | G | A | 0.023 | 0.1306 | 0.0079 | 4.54E-62 | 0.022 | -0.0178 | 0.0207 | 3.90E-01 | 343621 | 273.30 | TRUE | 1.46E-18 |
| LDL | rs7780562 | C | A | 0.773 | -0.0192 | 0.0026 | 2.98E-13 | 0.790 | -0.0030 | 0.0066 | 6.51E-01 | 343621 | 54.53 | TRUE | 8.92E-05 |
| LDL | rs77960347 | G | A | 0.013 | 0.0711 | 0.0102 | 2.99E-12 | 0.013 | -0.0227 | 0.0242 | 3.48E-01 | 343621 | 48.59 | TRUE | 5.79E-04 |
| LDL | rs7910135 | A | C | 0.479 | 0.0122 | 0.0022 | 1.50E-08 | 0.516 | 0.0030 | 0.0056 | 5.89E-01 | 343621 | 30.75 | TRUE | 4.56E-03 |
| LDL | rs79120103 | G | A | 0.031 | -0.0379 | 0.0068 | 2.86E-08 | 0.028 | -0.0020 | 0.0188 | 9.15E-01 | 343621 | 31.06 | TRUE | 2.18E-03 |
| LDL | rs79428416 | T | C | 0.046 | -0.0788 | 0.0056 | 2.22E-44 | 0.038 | 0.0194 | 0.0148 | 1.90E-01 | 343621 | 198.01 | TRUE | 5.27E-13 |
| LDL | rs80215559 | C | T | 0.078 | -0.0546 | 0.0044 | 2.96E-35 | 0.064 | 0.0213 | 0.0102 | 3.67E-02 | 343621 | 153.99 | TRUE | 1.86E-09 |
| LDL | rs80276949 | A | G | 0.023 | 0.0456 | 0.0079 | 6.18E-09 | 0.021 | -0.0030 | 0.0218 | 8.91E-01 | 343621 | 33.32 | TRUE | 1.59E-03 |
| LDL | rs9376090 | C | T | 0.274 | -0.0144 | 0.0024 | 1.22E-09 | 0.254 | -0.0070 | 0.0060 | 2.42E-01 | 343621 | 36.00 | TRUE | 4.91E-03 |
| LDL | rs9411465 | A | G | 0.057 | 0.0346 | 0.0049 | 2.65E-12 | 0.014 | 0.0345 | 0.0261 | 1.87E-01 | 343621 | 49.86 | TRUE | 1.24E-03 |
| LDL | rs952275 | G | T | 0.441 | 0.0754 | 0.0023 | 1.00E-200 | 0.494 | -0.0010 | 0.0049 | 8.39E-01 | 343621 | 1074.70 | TRUE | 1.29E-74 |
| LDL | rs9854574 | A | G | 0.079 | -0.0340 | 0.0040 | 8.02E-18 | 0.085 | -0.0020 | 0.0099 | 8.40E-01 | 343621 | 72.25 | TRUE | 3.11E-06 |
| LDL | rs9884390 | C | T | 0.237 | 0.0252 | 0.0028 | 2.80E-19 | 0.224 | -0.0010 | 0.0054 | 8.53E-01 | 343621 | 81.00 | TRUE | 6.73E-07 |
| LDL | rs9982111 | A | G | 0.554 | 0.0118 | 0.0021 | 2.94E-08 | 0.557 | -0.0020 | 0.0055 | 7.17E-01 | 343621 | 31.57 | TRUE | 2.98E-03 |
| LDL | rs998974 | C | T | 0.370 | 0.0127 | 0.0022 | 1.29E-08 | 0.408 | 0.0018 | 0.0056 | 7.46E-01 | 343621 | 33.32 | TRUE | 2.10E-03 |

Note: EAF values were rounded to three decimal places; β coefficients and standard errors were rounded to four decimal places; F statistics were rounded to two decimal places. P values were presented in scientific notation where appropriate. TRUE in the Steiger direction column indicates that the Steiger directionality test supported the exposure-to-outcome direction. All included SNPs had F statistics greater than 10, suggesting adequate instrument strength. Abbreviations: ED, erectile dysfunction; SNP, single nucleotide polymorphism; EAF, effect allele frequency; SE, standard error.
