# Supplementary material for: Magnesium depletion score and erectile dysfunction: A cross-sectional and Mendelian randomization study
Source: Medicine (Baltimore). 2026 Jul 24;105(30):e49938. doi: 10.1097/MD.0000000000049938 (PMC13406066; doi:10.1097/MD.0000000000049938)
Supplement: Supplementary file 6 [file medi-105-e49938-s006.docx]

Table S6. Instrumental variables for erectile dysfunction in reverse Mendelian randomization analyses.

| Outcome | SNP | Effect allele | Other allele | EAF (exposure) | β (exposure) | SE (exposure) | *P* value (exposure) | EAF (outcome) | Beta (outcome) | SE (outcome) | *P* value (outcome) | Sample size | F statistic | Steiger direction | Steiger *P* value |
| --- | --- | --- | --- | --- | --- | --- | --- | --- | --- | --- | --- | --- | --- | --- | --- |
| Disorders of magnesium metabolism | rs10822569 | G | A | 0.582 | -0.0297 | 0.0050 | 3.72E-09 | 0.658 | -0.0660 | 0.0987 | 5.04E-01 | 420066 | 34.76 | FALSE | 8.45E-01 |
| Disorders of magnesium metabolism | rs13135092 | A | G | 0.914 | -0.0628 | 0.0086 | 2.98E-13 | 0.982 | -0.2440 | 0.3569 | 4.94E-01 | 420066 | 53.22 | FALSE | 8.59E-01 |
| Disorders of magnesium metabolism | rs1427499 | A | G | 0.308 | 0.0354 | 0.0055 | 1.67E-10 | 0.306 | 0.0778 | 0.1010 | 4.41E-01 | 420066 | 40.82 | FALSE | 8.18E-01 |
| Disorders of magnesium metabolism | rs16912641 | G | A | 0.982 | -0.1113 | 0.0192 | 7.46E-09 | 0.959 | 0.0910 | 0.2371 | 7.01E-01 | 420066 | 33.41 | FALSE | 9.67E-01 |
| Disorders of magnesium metabolism | rs2249732 | G | A | 0.618 | -0.0342 | 0.0058 | 3.10E-09 | 0.529 | -0.0556 | 0.0950 | 5.58E-01 | 420066 | 35.12 | FALSE | 8.96E-01 |
| Disorders of magnesium metabolism | rs2347923 | C | A | 0.332 | 0.0315 | 0.0052 | 1.32E-09 | 0.326 | 0.1218 | 0.1002 | 2.24E-01 | 420066 | 36.79 | FALSE | 6.18E-01 |
| Disorders of magnesium metabolism | rs2555015 | T | C | 0.529 | 0.0286 | 0.0049 | 4.78E-09 | 0.469 | 0.0510 | 0.0938 | 5.86E-01 | 420066 | 34.28 | FALSE | 8.95E-01 |
| Disorders of magnesium metabolism | rs299572 | A | C | 0.621 | -0.0296 | 0.0051 | 8.62E-09 | 0.560 | 0.1087 | 0.0947 | 2.51E-01 | 420066 | 33.13 | FALSE | 6.42E-01 |
| Disorders of magnesium metabolism | rs3125575 | C | T | 0.615 | -0.0325 | 0.0054 | 1.57E-09 | 0.577 | 0.0286 | 0.0962 | 7.66E-01 | 420066 | 36.44 | TRUE | 9.84E-01 |
| Disorders of magnesium metabolism | rs3794995 | C | T | 0.675 | 0.0305 | 0.0053 | 7.50E-09 | 0.707 | -0.2579 | 0.0952 | 6.78E-03 | 420066 | 33.40 | FALSE | 2.23E-01 |
| Disorders of magnesium metabolism | rs3846410 | G | A | 0.650 | -0.0295 | 0.0052 | 1.43E-08 | 0.728 | 0.0669 | 0.1048 | 5.23E-01 | 420066 | 32.15 | FALSE | 8.54E-01 |
| Disorders of magnesium metabolism | rs4401545 | C | T | 0.740 | -0.0319 | 0.0056 | 1.55E-08 | 0.787 | -0.2084 | 0.1148 | 6.95E-02 | 420066 | 31.99 | FALSE | 3.98E-01 |
| Disorders of magnesium metabolism | rs56179563 | G | A | 0.617 | 0.0344 | 0.0052 | 3.18E-11 | 0.646 | -0.0707 | 0.0982 | 4.72E-01 | 420066 | 44.07 | FALSE | 8.40E-01 |
| Disorders of magnesium metabolism | rs60422795 | G | A | 0.559 | 0.0286 | 0.0050 | 9.46E-09 | 0.568 | -0.0131 | 0.0950 | 8.90E-01 | 420066 | 32.95 | TRUE | 9.28E-01 |
| Disorders of magnesium metabolism | rs7191353 | C | T | 0.728 | -0.0316 | 0.0055 | 7.52E-09 | 0.807 | -0.1020 | 0.1195 | 3.93E-01 | 420066 | 33.39 | FALSE | 7.57E-01 |
| Disorders of magnesium metabolism | rs78677597 | A | C | 0.753 | -0.1320 | 0.0057 | 1.12E-118 | 0.791 | -0.0840 | 0.1159 | 4.68E-01 | 420066 | 536.44 | TRUE | 7.93E-01 |
| Disorders of magnesium metabolism | rs8066520 | G | A | 0.848 | -0.0441 | 0.0069 | 2.03E-10 | 0.814 | -0.1939 | 0.1205 | 1.08E-01 | 420066 | 40.44 | FALSE | 4.79E-01 |
| Disorders of magnesium metabolism | rs8141413 | T | C | 0.354 | 0.0431 | 0.0052 | 2.31E-16 | 0.336 | 0.2656 | 0.0919 | 3.85E-03 | 420066 | 67.32 | FALSE | 2.15E-01 |
| BMI | rs10822569 | G | A | 0.582 | -0.0297 | 0.0050 | 3.72E-09 | 0.577 | -0.0008 | 0.0020 | 7.00E-01 | 454884 | 34.76 | TRUE | 1.02E-02 |
| BMI | rs12420076 | A | C | 0.993 | 0.2616 | 0.0433 | 1.55E-09 | 0.151 | -0.0023 | 0.0031 | 4.60E-01 | 454884 | 36.48 | TRUE | 1.26E-04 |
| BMI | rs1427499 | A | G | 0.308 | 0.0354 | 0.0055 | 1.67E-10 | 0.290 | -0.0012 | 0.0022 | 5.80E-01 | 454884 | 40.82 | TRUE | 5.22E-03 |
| BMI | rs16912641 | G | A | 0.982 | -0.1113 | 0.0192 | 7.46E-09 | 0.983 | 0.0050 | 0.0077 | 5.20E-01 | 454884 | 33.41 | TRUE | 1.60E-02 |
| BMI | rs2249732 | G | A | 0.618 | -0.0342 | 0.0058 | 3.10E-09 | 0.589 | 0.0001 | 0.0020 | 9.70E-01 | 454884 | 35.12 | TRUE | 1.88E-03 |
| BMI | rs2347923 | C | A | 0.332 | 0.0315 | 0.0052 | 1.32E-09 | 0.339 | 0.0025 | 0.0021 | 2.30E-01 | 454884 | 36.79 | TRUE | 2.86E-02 |
| BMI | rs2555015 | T | C | 0.529 | 0.0286 | 0.0049 | 4.78E-09 | 0.521 | -0.0005 | 0.0020 | 7.90E-01 | 454884 | 34.28 | TRUE | 1.06E-02 |
| BMI | rs299572 | A | C | 0.621 | -0.0296 | 0.0051 | 8.62E-09 | 0.599 | 0.0002 | 0.0020 | 9.30E-01 | 454884 | 33.13 | TRUE | 7.75E-03 |
| BMI | rs3125575 | C | T | 0.615 | -0.0325 | 0.0054 | 1.57E-09 | 0.614 | 0.0072 | 0.0020 | 4.60E-04 | 454884 | 36.44 | TRUE | 2.29E-01 |
| BMI | rs3794995 | C | T | 0.675 | 0.0305 | 0.0053 | 7.50E-09 | 0.670 | 0.0005 | 0.0021 | 8.30E-01 | 454884 | 33.40 | TRUE | 9.65E-03 |
| BMI | rs3846410 | G | A | 0.650 | -0.0295 | 0.0052 | 1.43E-08 | 0.646 | -0.0026 | 0.0021 | 2.20E-01 | 454884 | 32.15 | TRUE | 4.19E-02 |
| BMI | rs4401545 | C | T | 0.740 | -0.0319 | 0.0056 | 1.55E-08 | 0.734 | 0.0065 | 0.0023 | 4.20E-03 | 454884 | 31.99 | TRUE | 2.37E-01 |
| BMI | rs56179563 | G | A | 0.617 | 0.0344 | 0.0052 | 3.18E-11 | 0.612 | 0.0045 | 0.0021 | 3.00E-02 | 454884 | 44.07 | TRUE | 4.01E-02 |
| BMI | rs60422795 | G | A | 0.559 | 0.0286 | 0.0050 | 9.46E-09 | 0.543 | -0.0089 | 0.0020 | 7.60E-06 | 454884 | 32.95 | TRUE | 6.84E-01 |
| BMI | rs7191353 | C | T | 0.728 | -0.0316 | 0.0055 | 7.52E-09 | 0.716 | 0.0004 | 0.0022 | 8.50E-01 | 454884 | 33.39 | TRUE | 1.07E-02 |
| BMI | rs78677597 | A | C | 0.753 | -0.1320 | 0.0057 | 1.12E-118 | 0.756 | -0.0129 | 0.0023 | 2.80E-08 | 454884 | 536.44 | TRUE | 4.17E-15 |
| BMI | rs8066520 | G | A | 0.848 | -0.0441 | 0.0069 | 2.03E-10 | 0.846 | 0.0061 | 0.0028 | 2.90E-02 | 454884 | 40.44 | TRUE | 6.17E-02 |
| BMI | rs8141413 | T | C | 0.354 | 0.0431 | 0.0052 | 2.31E-16 | 0.336 | -0.0001 | 0.0021 | 9.70E-01 | 454884 | 67.32 | TRUE | 1.14E-04 |
| Diabetes | rs12420076 | A | C | 0.993 | 0.2616 | 0.0433 | 1.55E-09 | 0.151 | 0.0000 | 0.0006 | 9.30E-01 | 468298 | 36.48 | TRUE | 3.54E-01 |
| Diabetes | rs1427499 | A | G | 0.308 | 0.0354 | 0.0055 | 1.67E-10 | 0.289 | 0.0000 | 0.0005 | 9.50E-01 | 468298 | 40.82 | TRUE | 4.98E-01 |
| Diabetes | rs16912641 | G | A | 0.982 | -0.1113 | 0.0192 | 7.46E-09 | 0.983 | -0.0028 | 0.0016 | 8.30E-02 | 468298 | 33.41 | TRUE | 5.56E-01 |
| Diabetes | rs2249732 | G | A | 0.618 | -0.0342 | 0.0058 | 3.10E-09 | 0.589 | -0.0006 | 0.0004 | 1.10E-01 | 468298 | 35.12 | TRUE | 4.98E-01 |
| Diabetes | rs2347923 | C | A | 0.332 | 0.0315 | 0.0052 | 1.32E-09 | 0.339 | 0.0005 | 0.0004 | 2.80E-01 | 468298 | 36.79 | TRUE | 5.44E-01 |
| Diabetes | rs2555015 | T | C | 0.529 | 0.0286 | 0.0049 | 4.78E-09 | 0.521 | 0.0006 | 0.0004 | 1.80E-01 | 468298 | 34.28 | TRUE | 5.61E-01 |
| Diabetes | rs299572 | A | C | 0.621 | -0.0296 | 0.0051 | 8.62E-09 | 0.599 | -0.0001 | 0.0004 | 7.70E-01 | 468298 | 33.13 | TRUE | 5.52E-01 |
| Diabetes | rs3125575 | C | T | 0.615 | -0.0325 | 0.0054 | 1.57E-09 | 0.614 | 0.0000 | 0.0004 | 9.20E-01 | 468298 | 36.44 | TRUE | 5.11E-01 |
| Diabetes | rs3794995 | C | T | 0.675 | 0.0305 | 0.0053 | 7.50E-09 | 0.670 | 0.0004 | 0.0004 | 2.90E-01 | 468298 | 33.40 | TRUE | 5.58E-01 |
| Diabetes | rs3846410 | G | A | 0.650 | -0.0295 | 0.0052 | 1.43E-08 | 0.647 | 0.0003 | 0.0004 | 5.00E-01 | 468298 | 32.15 | TRUE | 5.62E-01 |
| Diabetes | rs56179563 | G | A | 0.617 | 0.0344 | 0.0052 | 3.18E-11 | 0.612 | 0.0006 | 0.0004 | 1.50E-01 | 468298 | 44.07 | TRUE | 4.94E-01 |
| Diabetes | rs60422795 | G | A | 0.559 | 0.0286 | 0.0050 | 9.46E-09 | 0.542 | -0.0001 | 0.0004 | 7.80E-01 | 468298 | 32.95 | TRUE | 5.57E-01 |
| Diabetes | rs7191353 | C | T | 0.728 | -0.0316 | 0.0055 | 7.52E-09 | 0.716 | 0.0001 | 0.0005 | 7.90E-01 | 468298 | 33.39 | TRUE | 5.61E-01 |
| Diabetes | rs78677597 | A | C | 0.753 | -0.1320 | 0.0057 | 1.12E-118 | 0.756 | -0.0001 | 0.0005 | 9.10E-01 | 468298 | 536.44 | TRUE | 1.87E-02 |
| Diabetes | rs8066520 | G | A | 0.848 | -0.0441 | 0.0069 | 2.03E-10 | 0.846 | -0.0003 | 0.0006 | 6.50E-01 | 468298 | 40.44 | TRUE | 5.14E-01 |
| Diabetes | rs8141413 | T | C | 0.354 | 0.0431 | 0.0052 | 2.31E-16 | 0.336 | 0.0004 | 0.0004 | 3.20E-01 | 468298 | 67.32 | TRUE | 3.97E-01 |
| HDL | rs10822569 | G | A | 0.582 | -0.0297 | 0.0050 | 3.72E-09 | 0.616 | 0.0024 | 0.0021 | 2.52E-01 | 315133 | 34.76 | TRUE | 5.01E-02 |
| HDL | rs12420076 | A | C | 0.993 | 0.2616 | 0.0433 | 1.55E-09 | 0.886 | -0.0052 | 0.0077 | 4.99E-01 | 315133 | 36.48 | TRUE | 3.24E-04 |
| HDL | rs1427499 | A | G | 0.308 | 0.0354 | 0.0055 | 1.67E-10 | 0.283 | 0.0018 | 0.0023 | 4.41E-01 | 315133 | 40.82 | TRUE | 1.39E-02 |
| HDL | rs16912641 | G | A | 0.982 | -0.1113 | 0.0192 | 7.46E-09 | 0.982 | -0.0040 | 0.0079 | 6.11E-01 | 315133 | 33.41 | TRUE | 2.14E-02 |
| HDL | rs2249732 | G | A | 0.618 | -0.0342 | 0.0058 | 3.10E-09 | 0.573 | 0.0018 | 0.0047 | 6.97E-01 | 315133 | 35.12 | TRUE | 6.07E-03 |
| HDL | rs2347923 | C | A | 0.332 | 0.0315 | 0.0052 | 1.32E-09 | 0.390 | 0.0001 | 0.0021 | 9.63E-01 | 315133 | 36.79 | TRUE | 8.81E-03 |
| HDL | rs2555015 | T | C | 0.529 | 0.0286 | 0.0049 | 4.78E-09 | 0.471 | -0.0007 | 0.0021 | 7.39E-01 | 315133 | 34.28 | TRUE | 1.84E-02 |
| HDL | rs299572 | A | C | 0.621 | -0.0296 | 0.0051 | 8.62E-09 | 0.624 | 0.0008 | 0.0021 | 7.24E-01 | 315133 | 33.13 | TRUE | 1.90E-02 |
| HDL | rs3125575 | C | T | 0.615 | -0.0325 | 0.0054 | 1.57E-09 | 0.654 | -0.0053 | 0.0022 | 1.59E-02 | 315133 | 36.44 | TRUE | 1.51E-01 |
| HDL | rs3794995 | C | T | 0.675 | 0.0305 | 0.0053 | 7.50E-09 | 0.677 | 0.0001 | 0.0023 | 9.51E-01 | 315133 | 33.40 | TRUE | 1.14E-02 |
| HDL | rs3846410 | G | A | 0.650 | -0.0295 | 0.0052 | 1.43E-08 | 0.687 | -0.0012 | 0.0022 | 5.86E-01 | 315133 | 32.15 | TRUE | 2.78E-02 |
| HDL | rs4401545 | C | T | 0.740 | -0.0319 | 0.0056 | 1.55E-08 | 0.707 | -0.0040 | 0.0023 | 8.14E-02 | 315133 | 31.99 | TRUE | 1.36E-01 |
| HDL | rs56179563 | G | A | 0.617 | 0.0344 | 0.0052 | 3.18E-11 | 0.601 | -0.0021 | 0.0022 | 3.19E-01 | 315133 | 44.07 | TRUE | 1.47E-02 |
| HDL | rs60422795 | G | A | 0.559 | 0.0286 | 0.0050 | 9.46E-09 | 0.593 | 0.0052 | 0.0021 | 1.49E-02 | 315133 | 32.95 | TRUE | 2.68E-01 |
| HDL | rs7191353 | C | T | 0.728 | -0.0316 | 0.0055 | 7.52E-09 | 0.767 | -0.0031 | 0.0025 | 2.18E-01 | 315133 | 33.39 | TRUE | 7.32E-02 |
| HDL | rs78677597 | A | C | 0.753 | -0.1320 | 0.0057 | 1.12E-118 | 0.757 | 0.0010 | 0.0027 | 7.09E-01 | 315133 | 536.44 | TRUE | 4.48E-23 |
| HDL | rs8066520 | G | A | 0.848 | -0.0441 | 0.0069 | 2.03E-10 | 0.821 | -0.0011 | 0.0027 | 6.93E-01 | 315133 | 40.44 | TRUE | 9.74E-03 |
| LDL | rs10822569 | G | A | 0.582 | -0.0297 | 0.0050 | 3.72E-09 | 0.612 | 0.0001 | 0.0022 | 9.72E-01 | 343621 | 34.76 | TRUE | 8.53E-03 |
| LDL | rs12420076 | A | C | 0.993 | 0.2616 | 0.0433 | 1.55E-09 | 0.887 | -0.0079 | 0.0079 | 3.17E-01 | 343621 | 36.48 | TRUE | 5.01E-04 |
| LDL | rs1427499 | A | G | 0.308 | 0.0354 | 0.0055 | 1.67E-10 | 0.283 | 0.0013 | 0.0023 | 5.87E-01 | 343621 | 40.82 | TRUE | 8.39E-03 |
| LDL | rs2249732 | G | A | 0.618 | -0.0342 | 0.0058 | 3.10E-09 | 0.571 | -0.0062 | 0.0048 | 1.96E-01 | 343621 | 35.12 | TRUE | 2.25E-02 |
| LDL | rs2347923 | C | A | 0.332 | 0.0315 | 0.0052 | 1.32E-09 | 0.385 | 0.0018 | 0.0022 | 4.08E-01 | 343621 | 36.79 | TRUE | 2.60E-02 |
| LDL | rs2555015 | T | C | 0.529 | 0.0286 | 0.0049 | 4.78E-09 | 0.476 | -0.0043 | 0.0022 | 4.43E-02 | 343621 | 34.28 | TRUE | 1.36E-01 |
| LDL | rs299572 | A | C | 0.621 | -0.0296 | 0.0051 | 8.62E-09 | 0.622 | 0.0023 | 0.0022 | 2.92E-01 | 343621 | 33.13 | TRUE | 4.38E-02 |
| LDL | rs3125575 | C | T | 0.615 | -0.0325 | 0.0054 | 1.57E-09 | 0.650 | -0.0015 | 0.0023 | 5.18E-01 | 343621 | 36.44 | TRUE | 1.25E-02 |
| LDL | rs3794995 | C | T | 0.675 | 0.0305 | 0.0053 | 7.50E-09 | 0.676 | -0.0027 | 0.0023 | 2.42E-01 | 343621 | 33.40 | TRUE | 5.31E-02 |
| LDL | rs3846410 | G | A | 0.650 | -0.0295 | 0.0052 | 1.43E-08 | 0.683 | 0.0014 | 0.0023 | 5.42E-01 | 343621 | 32.15 | TRUE | 2.72E-02 |
| LDL | rs4401545 | C | T | 0.740 | -0.0319 | 0.0056 | 1.55E-08 | 0.710 | 0.0002 | 0.0023 | 9.32E-01 | 343621 | 31.99 | TRUE | 1.31E-02 |
| LDL | rs56179563 | G | A | 0.617 | 0.0344 | 0.0052 | 3.18E-11 | 0.601 | -0.0038 | 0.0022 | 8.43E-02 | 343621 | 44.07 | TRUE | 3.90E-02 |
| LDL | rs60422795 | G | A | 0.559 | 0.0286 | 0.0050 | 9.46E-09 | 0.589 | -0.0001 | 0.0022 | 9.75E-01 | 343621 | 32.95 | TRUE | 1.09E-02 |
| LDL | rs7191353 | C | T | 0.728 | -0.0316 | 0.0055 | 7.52E-09 | 0.762 | 0.0023 | 0.0026 | 3.76E-01 | 343621 | 33.39 | TRUE | 4.07E-02 |
| LDL | rs8066520 | G | A | 0.848 | -0.0441 | 0.0069 | 2.03E-10 | 0.823 | 0.0039 | 0.0028 | 1.63E-01 | 343621 | 40.44 | TRUE | 3.76E-02 |
| LDL | rs8141413 | T | C | 0.354 | 0.0431 | 0.0052 | 2.31E-16 | 0.372 | 0.0017 | 0.0022 | 4.55E-01 | 343621 | 67.32 | TRUE | 9.94E-04 |

Note: EAF values were rounded to three decimal places; β coefficients and standard errors were rounded to four decimal places; F statistics were rounded to two decimal places. P values were presented in scientific notation where appropriate. TRUE in the Steiger direction column indicates that the Steiger directionality test supported the exposure-to-outcome direction. All included SNPs had F statistics greater than 10, suggesting adequate instrument strength. Abbreviations: ED, erectile dysfunction; SNP, single nucleotide polymorphism; EAF, effect allele frequency; SE, standard error.
