# Supplementary material for: Magnesium depletion score and erectile dysfunction: A cross-sectional and Mendelian randomization study
Source: Medicine (Baltimore). 2026 Jul 24;105(30):e49938. doi: 10.1097/MD.0000000000049938 (PMC13406066; doi:10.1097/MD.0000000000049938)
Supplement: Supplementary file 7 [file medi-105-e49938-s007.docx]

Table S7. Forward and reverse Mendelian randomization analyses of exposures and erectile dysfunction.

| Analysis | Exposure | Outcome | MR method | No. of SNPs | Effect estimate (95% CI) | *P* value | Heterogeneity *P* | MR-Egger intercept *P* | MR-PRESSO *P* |
| --- | --- | --- | --- | --- | --- | --- | --- | --- | --- |
| Forward MR | Disorders of magnesium metabolism | ED | MR-Egger | 5 | 0.99 (0.89, 1.10) | 0.891 | 0.072 |  |  |
|  |  |  | Weighted median |  | 0.99 (0.97, 1.01) | 0.541 |  |  |  |
|  |  |  | IVW |  | 1.00 (0.98, 1.01) | 0.597 | 0.136 | 0.956 | 0.191 (raw, 0 outliers) |
|  |  |  | Simple mode |  | 1.00 (0.96, 1.03) | 0.826 |  |  |  |
|  |  |  | Weighted mode |  | 0.99 (0.96, 1.02) | 0.681 |  |  |  |
|  | BMI |  | MR-Egger | 306 | 1.15 (1.04, 1.28) | 0.009 | 0.802 |  |  |
|  |  |  | Weighted median |  | 1.17 (1.08, 1.26) | < 0.001 |  |  |  |
|  |  |  | IVW |  | 1.16 (1.11, 1.20) | < 0.001 | 0.813 | 0.978 | 0.815 (outlier corrected, 62 outliers) |
|  |  |  | Simple mode |  | 1.31 (1.06, 1.62) | 0.014 |  |  |  |
|  |  |  | Weighted mode |  | 1.20 (1.07, 1.34) | 0.001 |  |  |  |
|  | Type 2 diabetes |  | MR-Egger | 49 | 3.00 (1.19, 7.58) | 0.024 | 0.294 |  |  |
|  |  |  | Weighted median |  | 3.81 (1.91, 7.59) | < 0.001 |  |  |  |
|  |  |  | IVW |  | 5.02 (3.24, 7.76) | < 0.001 | 0.274 | 0.224 | 0.323 (outlier corrected, 4 outliers) |
|  |  |  | Simple mode |  | 9.28 (2.36, 36.53) | 0.003 |  |  |  |
|  |  |  | Weighted mode |  | 5.03 (2.38, 10.66) | < 0.001 |  |  |  |
|  | HDL-C |  | MR-Egger | 201 | 0.96 (0.92, 1.00) | 0.066 | 0.788 |  |  |
|  |  |  | Weighted median |  | 0.96 (0.91, 1.01) | 0.130 |  |  |  |
|  |  |  | IVW |  | 0.94 (0.92, 0.97) | < 0.001 | 0.781 | 0.236 | 0.772 (outlier corrected, 35 outliers) |
|  |  |  | Simple mode |  | 0.91 (0.82, 1.01) | 0.074 |  |  |  |
|  |  |  | Weighted mode |  | 0.95 (0.91, 0.99) | 0.019 |  |  |  |
|  | LDL-C |  | MR-Egger | 124 | 0.94 (0.91, 0.98) | 0.001 | 0.831 |  |  |
|  |  |  | Weighted median |  | 0.94 (0.91, 0.97) | 0.001 |  |  |  |
|  |  |  | IVW |  | 0.93 (0.91, 0.96) | < 0.001 | 0.813 | 0.165 | 0.847 (outlier corrected, 17 outliers) |
|  |  |  | Simple mode |  | 0.88 (0.79, 0.99) | 0.043 |  |  |  |
|  |  |  | Weighted mode |  | 0.94 (0.91, 0.97) | < 0.001 |  |  |  |
| Reverse MR | ED | Disorders of magnesium metabolism | MR-Egger | 18 | 2.55 (0.16, 40.53) | 0.516 | 0.049 |  |  |
|  |  |  | Weighted median |  | 2.19 (0.47, 10.27) | 0.319 |  |  |  |
|  |  |  | IVW |  | 2.46 (0.63, 9.63) | 0.197 | 0.068 | 0.975 | 0.092 (raw, 0 outliers) |
|  |  |  | Simple mode |  | 8.72 (0.41, 187.44) | 0.184 |  |  |  |
|  |  |  | Weighted mode |  | 1.85 (0.36, 9.43) | 0.468 |  |  |  |
|  |  | BMI | MR-Egger | 12 | -0.013 (-0.040, 0.014) | 0.363 | 0.969 |  |  |
|  |  |  | Weighted median |  | -0.008 (-0.033, 0.016) | 0.503 |  |  |  |
|  |  |  | IVW |  | -0.005 (-0.025, 0.015) | 0.647 | 0.961 | 0.382 | 0.953 (outlier corrected, 6 outliers) |
|  |  |  | Simple mode |  | -0.008 (-0.060, 0.045) | 0.784 |  |  |  |
|  |  |  | Weighted mode |  | -0.008 (-0.031, 0.014) | 0.476 |  |  |  |
|  |  | Type 2 diabetes | MR-Egger | 16 | 1.00 (0.99, 1.00) | 0.790 | 0.800 |  |  |
|  |  |  | Weighted median |  | 1.00 (1.00, 1.00) | 0.955 |  |  |  |
|  |  |  | IVW |  | 1.00 (1.00, 1.01) | 0.214 | 0.674 | 0.130 | 0.625 (outlier corrected, 3 outliers) |
|  |  |  | Simple mode |  | 1.00 (0.99, 1.01) | 0.960 |  |  |  |
|  |  |  | Weighted mode |  | 1.00 (1.00, 1.00) | 0.939 |  |  |  |
|  |  | HDL-C | MR-Egger | 17 | -0.021 (-0.065, 0.024) | 0.376 | 0.294 |  |  |
|  |  |  | Weighted median |  | -0.008 (-0.042, 0.026) | 0.654 |  |  |  |
|  |  |  | IVW |  | 0.009 (-0.019, 0.037) | 0.519 | 0.198 | 0.123 | 0.212 (raw, 0 outliers) |
|  |  |  | Simple mode |  | -0.006 (-0.065, 0.054) | 0.854 |  |  |  |
|  |  |  | Weighted mode |  | -0.010 (-0.043, 0.024) | 0.571 |  |  |  |
|  |  | LDL-C | MR-Egger | 16 | -0.023 (-0.090, 0.043) | 0.500 | 0.422 |  |  |
|  |  |  | Weighted median |  | -0.024 (-0.069, 0.022) | 0.305 |  |  |  |
|  |  |  | IVW |  | -0.026 (-0.057, 0.005) | 0.102 | 0.497 | 0.497 | 0.518 (outlier corrected, 3 outliers) |
|  |  |  | Simple mode |  | -0.033 (-0.113, 0.047) | 0.429 |  |  |  |
|  |  |  | Weighted mode |  | -0.024 (-0.080, 0.032) | 0.411 |  |  |  |

Note: IVW was used as the primary MR method, and MR-Egger, weighted median, simple mode, and weighted mode were used as sensitivity analyses. Effect estimates are presented as ORs with 95% CIs for binary outcomes, including ED, disorders of magnesium metabolism, and type 2 diabetes, and as β coefficients with 95% CIs for continuous outcomes, including BMI, HDL-C, and LDL-C. Heterogeneity P values were obtained from Cochran’s Q test. MR-Egger intercept P values were used to assess directional pleiotropy. MR-PRESSO P values refer to the global test and are presented after outlier correction where applicable. Abbreviations: BMI, body mass index; CI, confidence interval; ED, erectile dysfunction; HDL-C, high-density lipoprotein cholesterol; IVW, inverse variance weighted; LDL-C, low-density lipoprotein cholesterol; MR, Mendelian randomization; MR-PRESSO, Mendelian Randomization Pleiotropy RESidual Sum and Outlier; OR, odds ratio; SNP, single nucleotide polymorphism.
