# Supplementary material for: Magnesium depletion score and erectile dysfunction: A cross-sectional and Mendelian randomization study
Source: Medicine (Baltimore). 2026 Jul 24;105(30):e49938. doi: 10.1097/MD.0000000000049938 (PMC13406066; doi:10.1097/MD.0000000000049938)
Supplement: Supplementary file 8 [file medi-105-e49938-s008.pdf]

S2 Fig. Sensitivity analyses of forward and reverse MR between magnesium metabolism, metabolic traits, and ED.

Forward MR

Disorders of magnesium metabolism

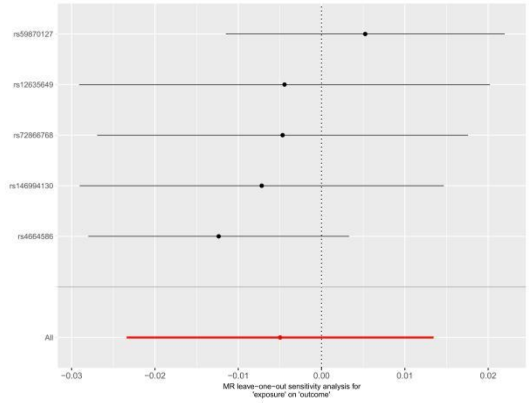

Leave-one-out analysis

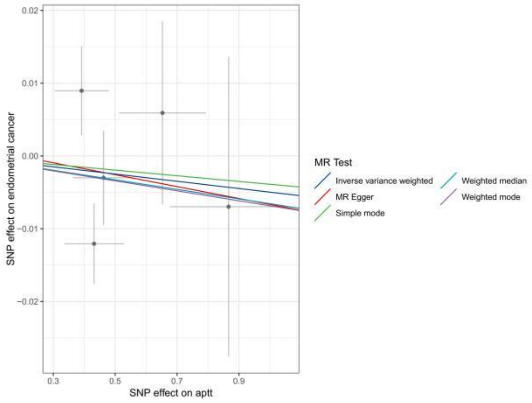

Scatter plot

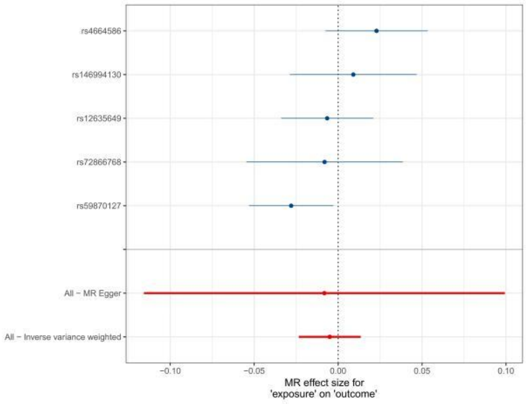

Forest plot

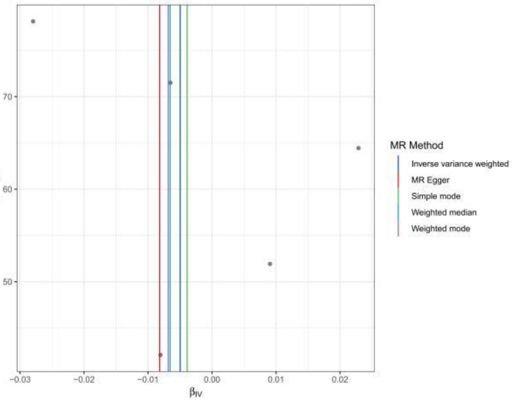

Funnel plot

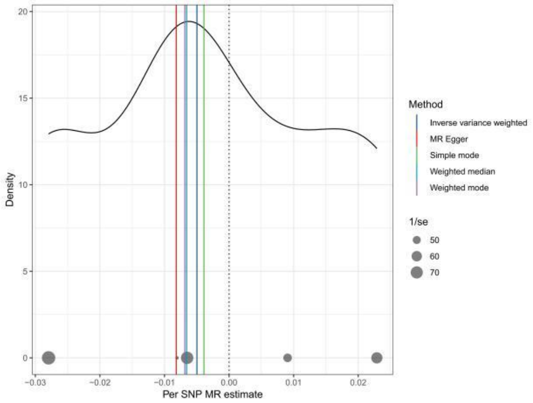

Density plot

BMI

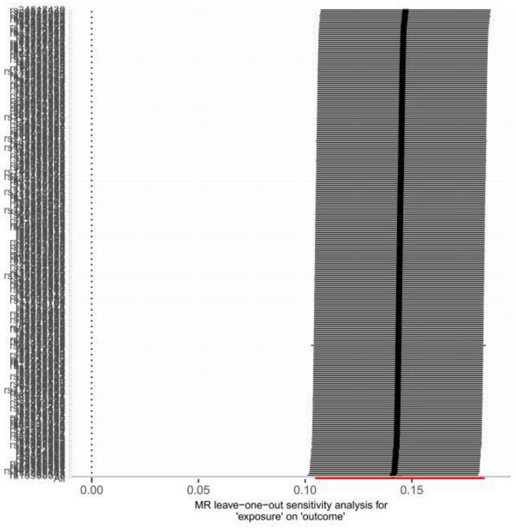

Leave-one-out analysis

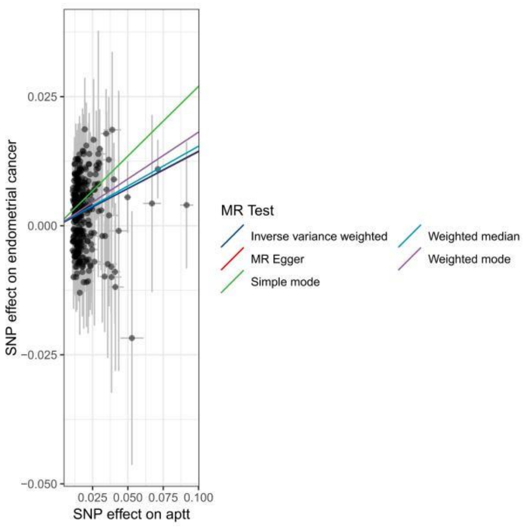

Scatter plot

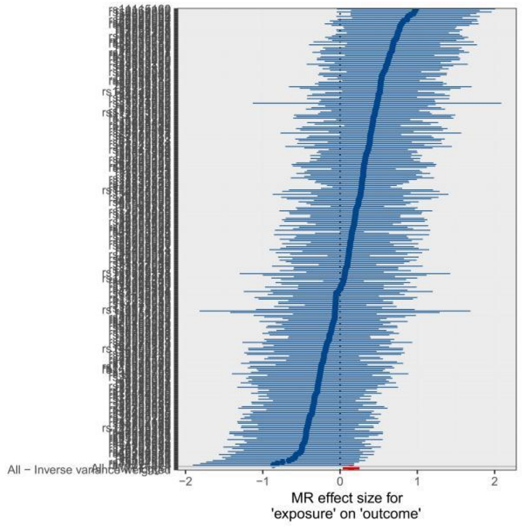

Forest plot

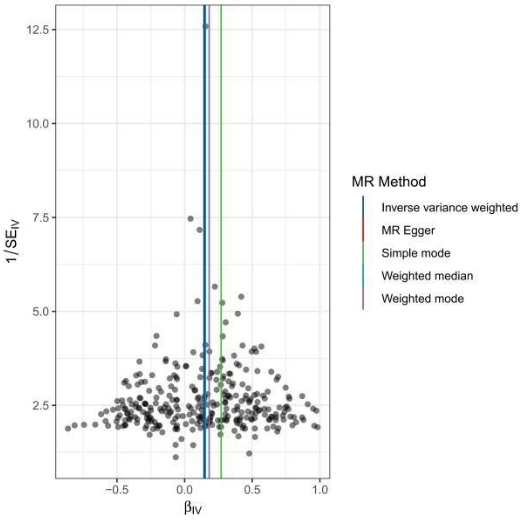

Funnel plot

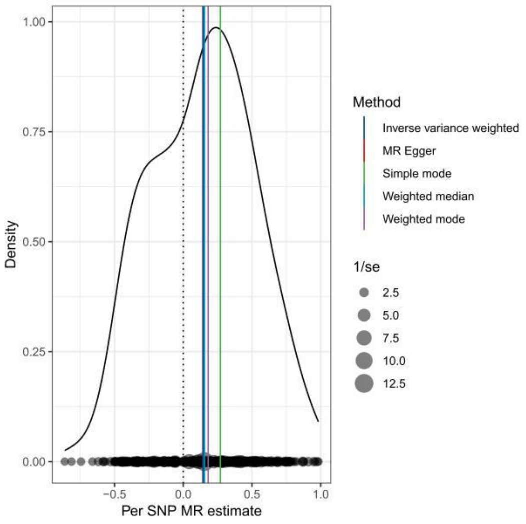

Density plot

Diabetes

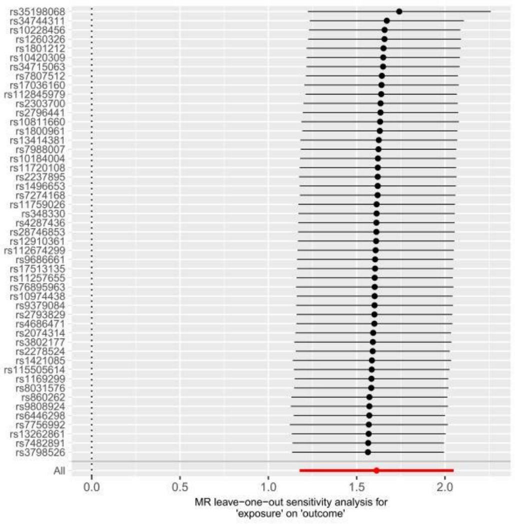

Leave-one-out analysis

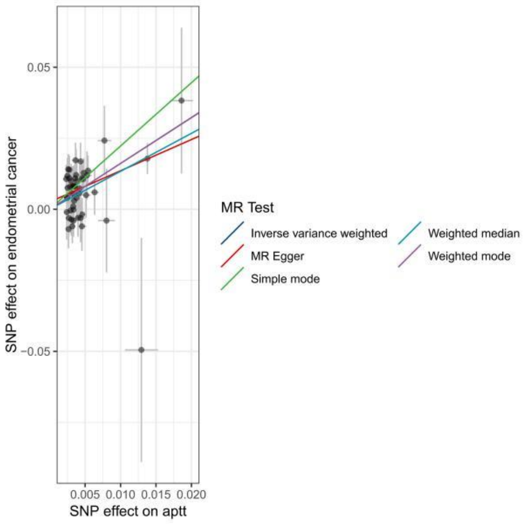

Scatter plot

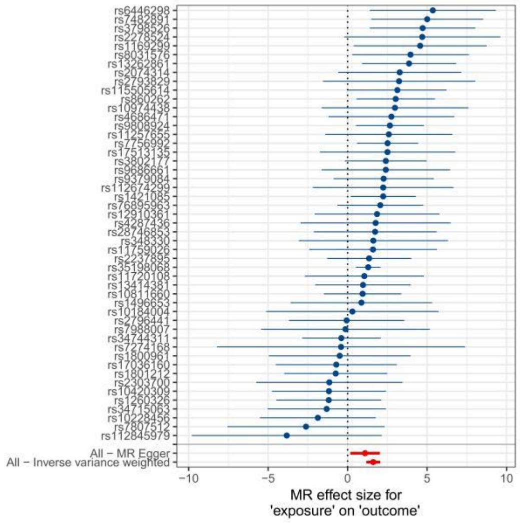

Forest plot

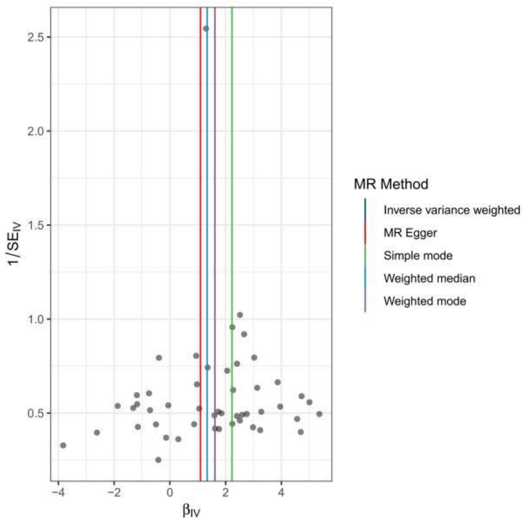

Funnel plot

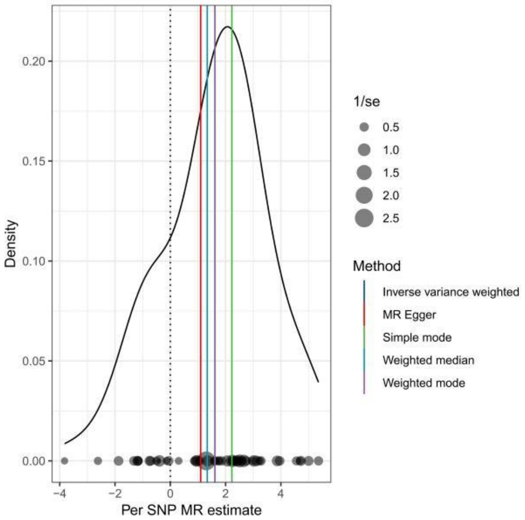

Density plot

HDL-C

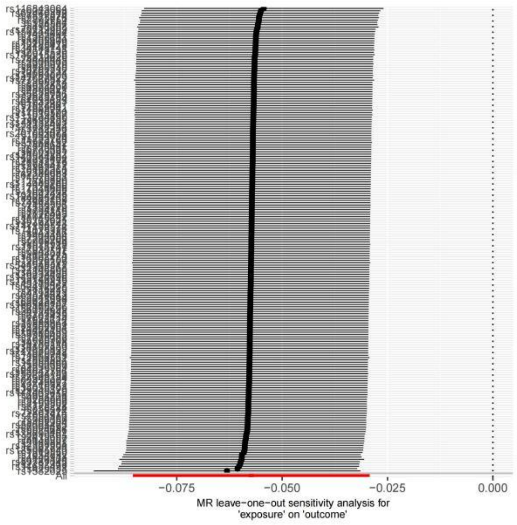

Leave-one-out analysis

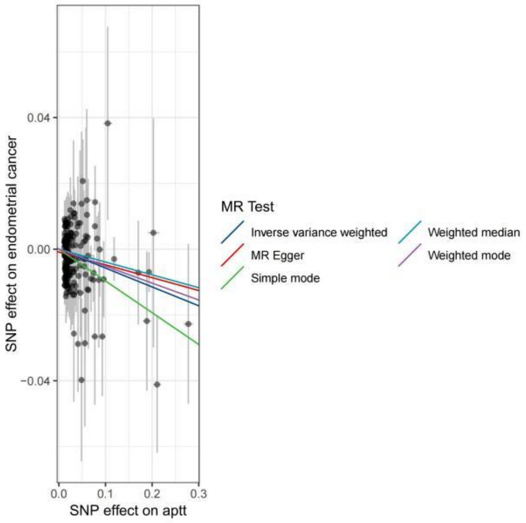

Scatter plot

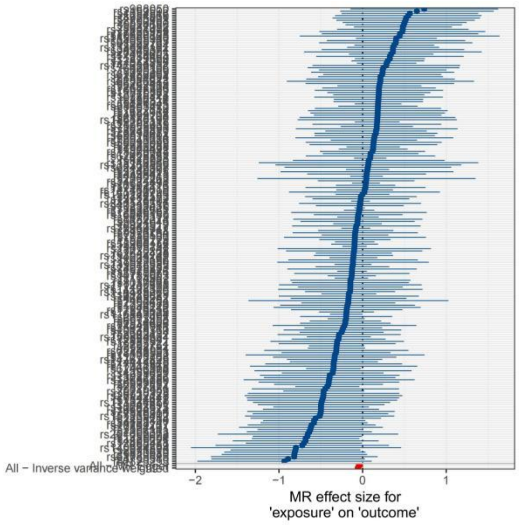

Forest plot

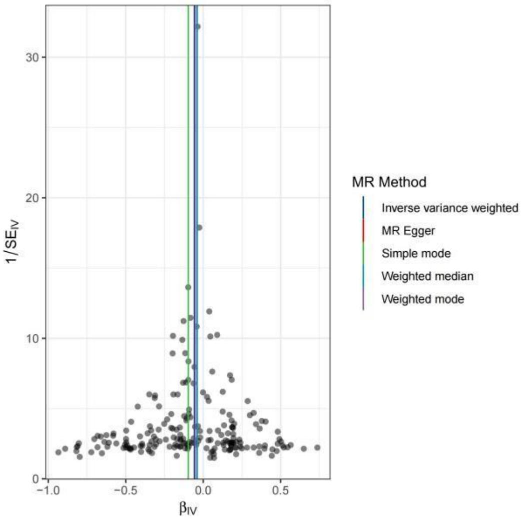

Funnel plot

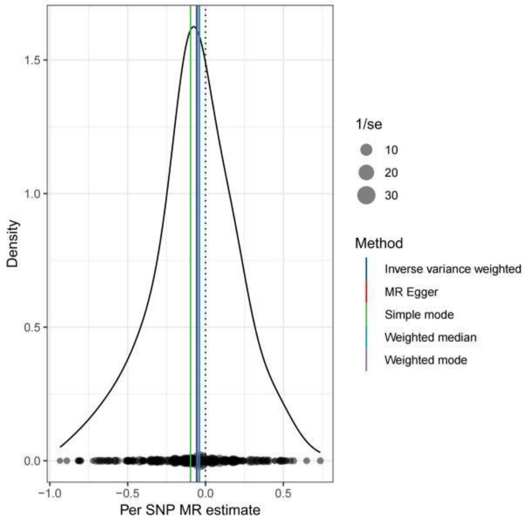

Density plot

LDL-C

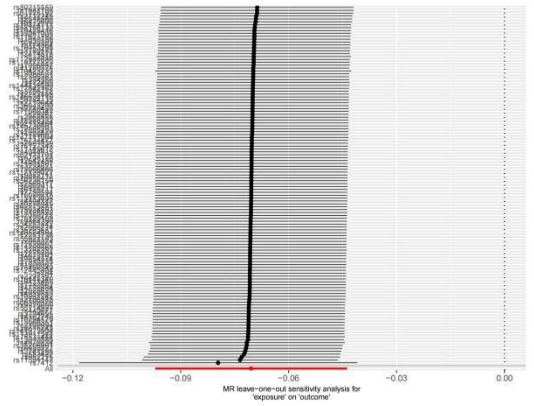

Leave-one-out analysis

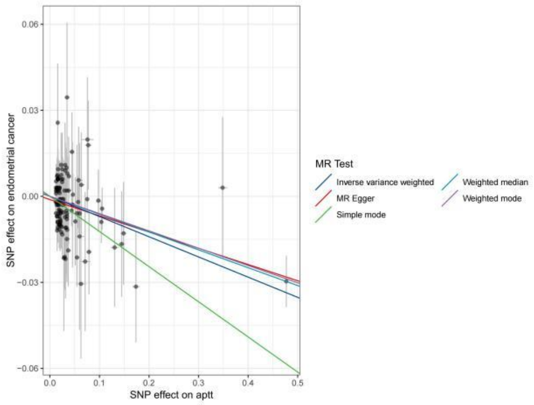

Scatter plot

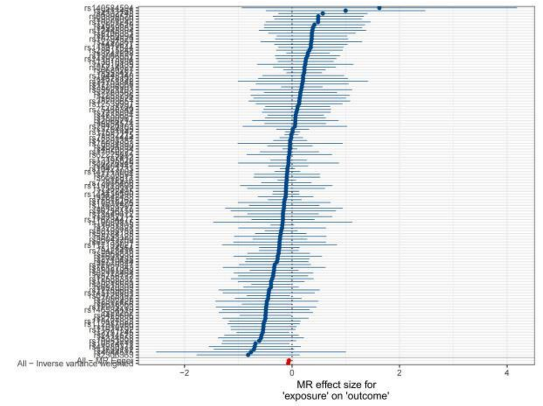

Forest plot

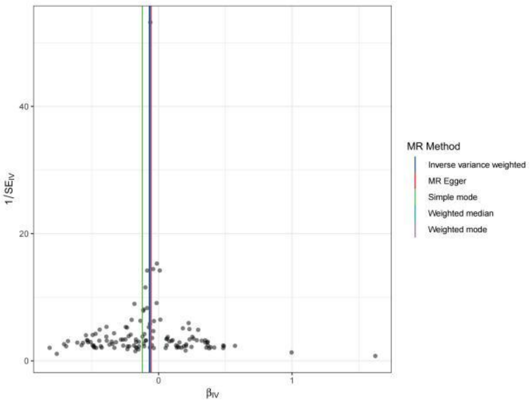

Funnel plot

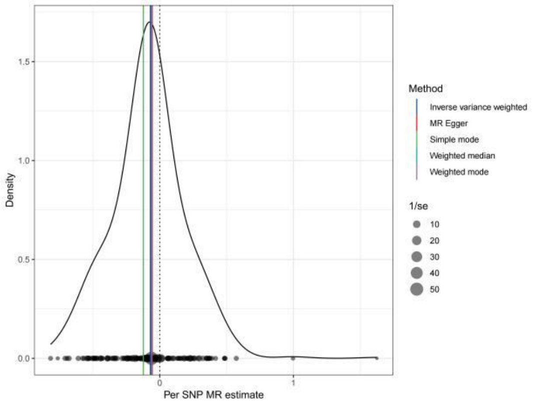

Density plot

Reverse MR

Disorders of magnesium metabolism

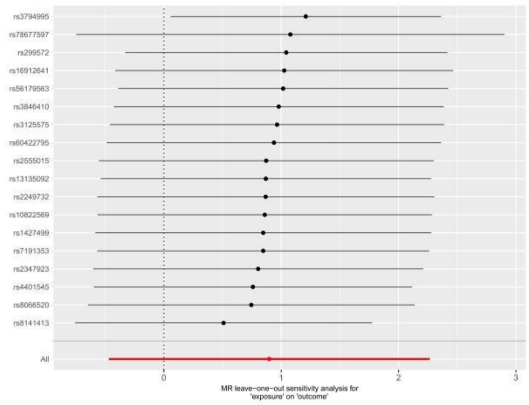

Leave-one-out analysis

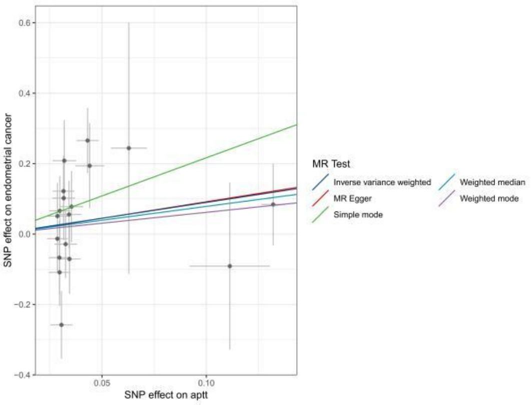

Scatter plot

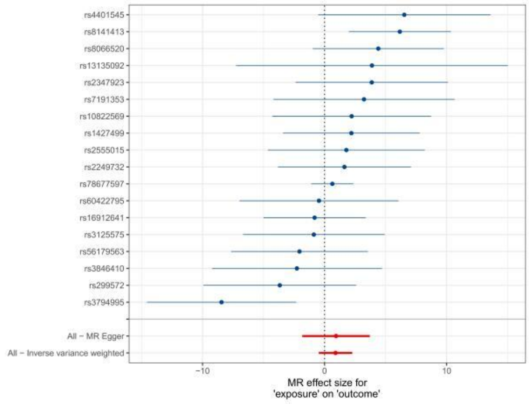

Forest plot

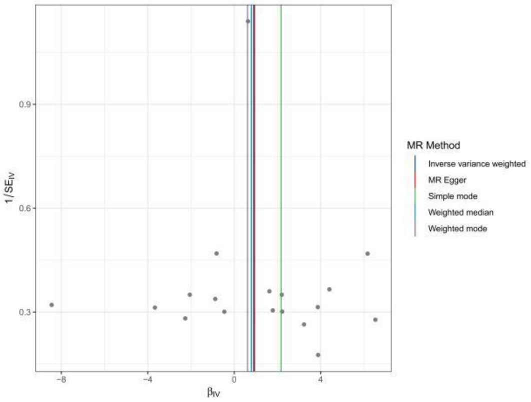

Funnel plot

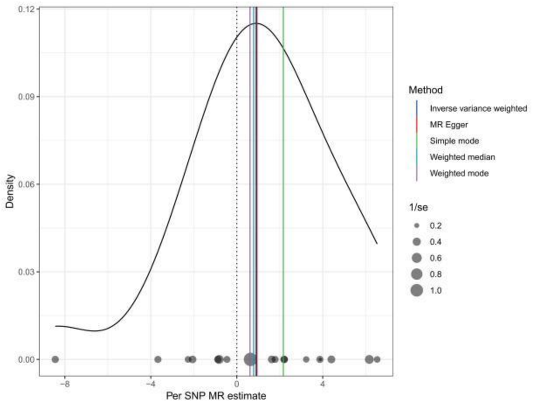

Density plot

BMI

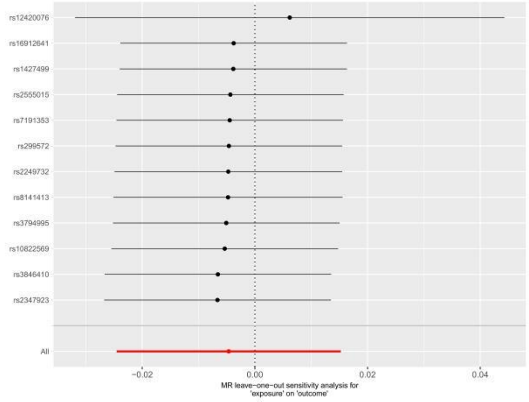

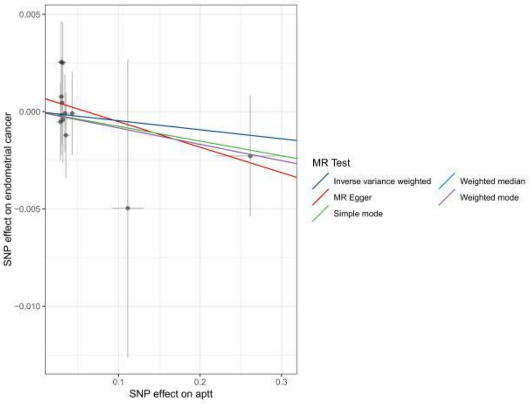

Leave-one-out analysis

Scatter plot

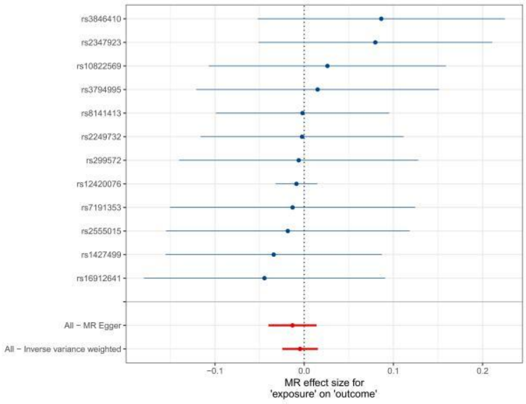

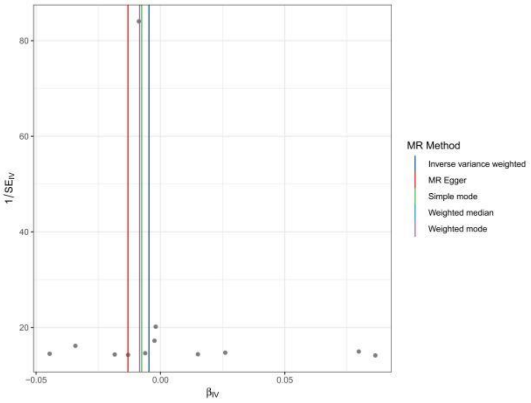

Forest plot

Funnel plot

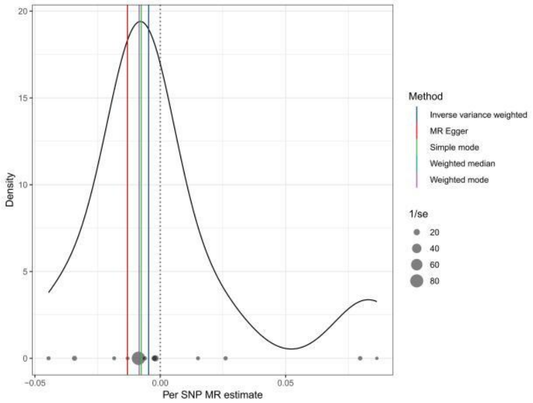

Density plot

Diabetes

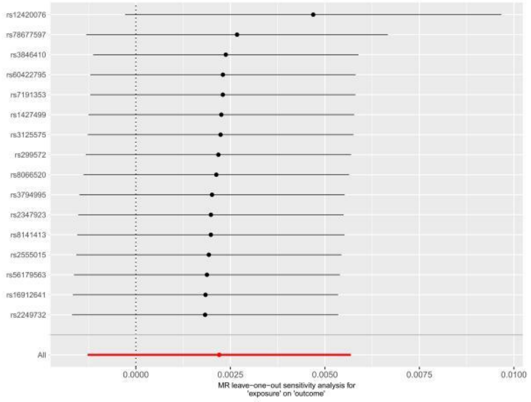

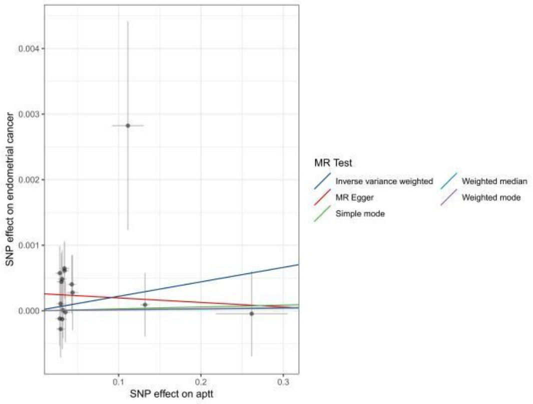

Leave-one-out analysis

Scatter plot

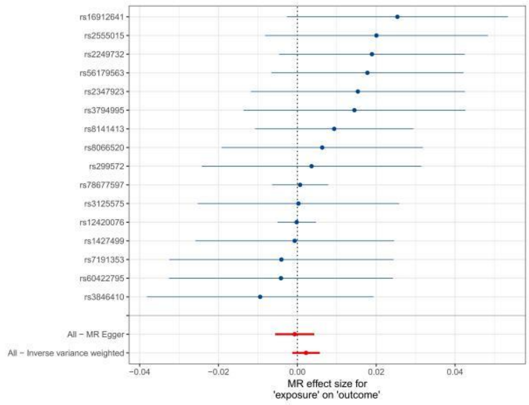

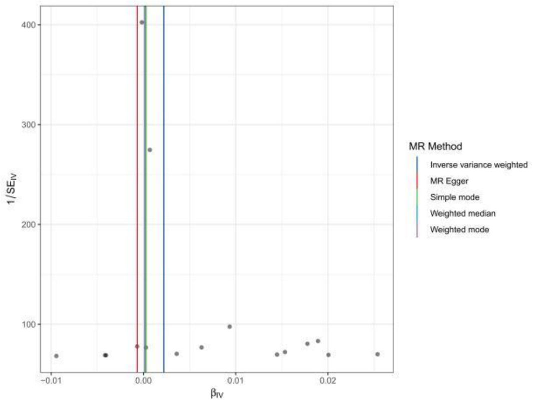

Forest plot

Funnel plot

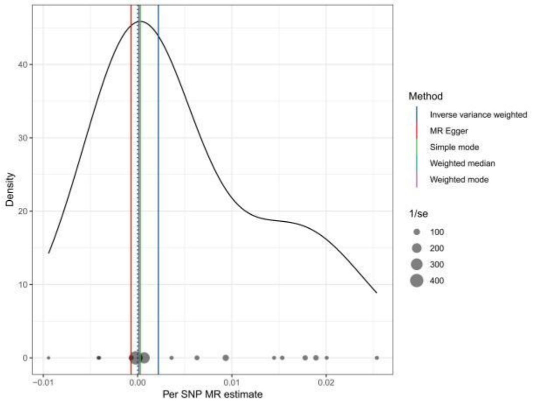

Density plot

HDL-C

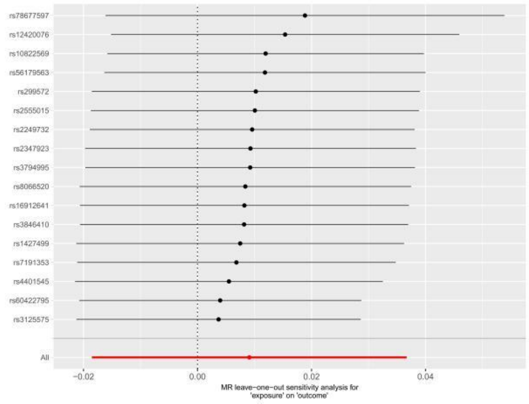

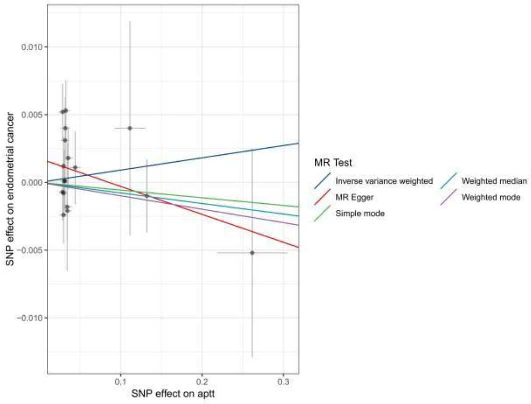

Leave-one-out analysis

Scatter plot

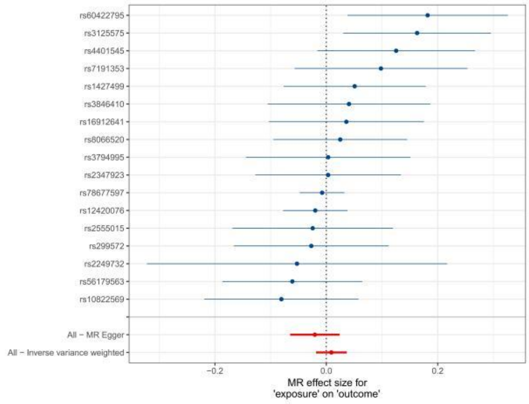

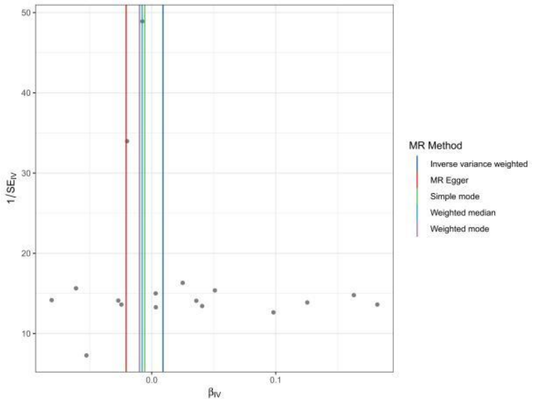

Forest plot

Funnel plot

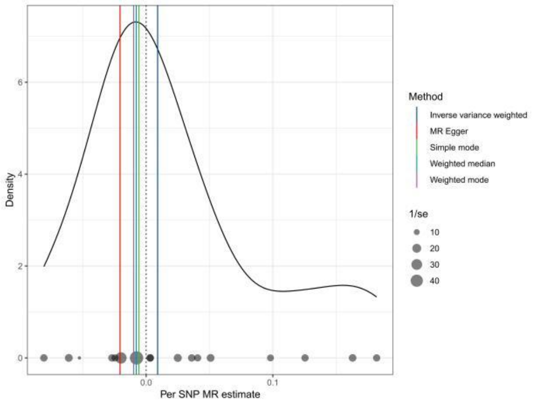

Density plot

LDL-C

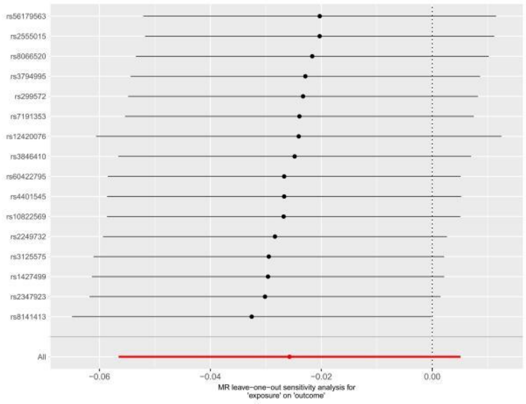

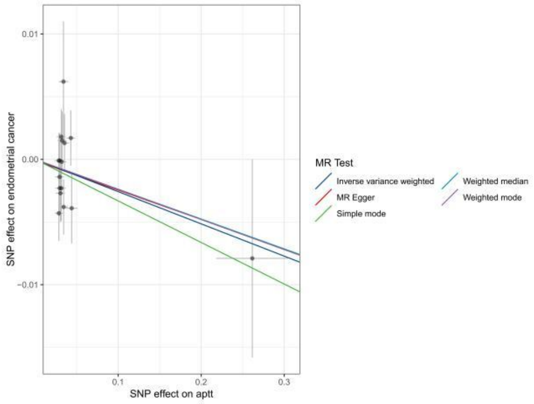

Leave-one-out analysis

Scatter plot

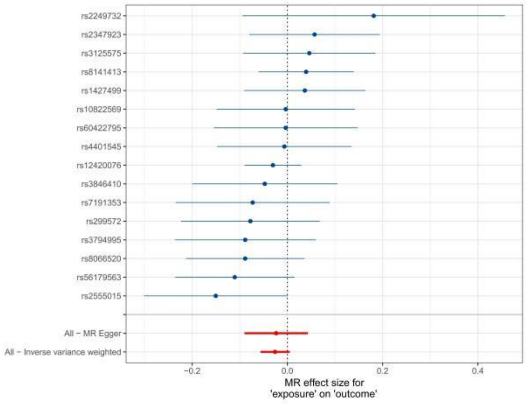

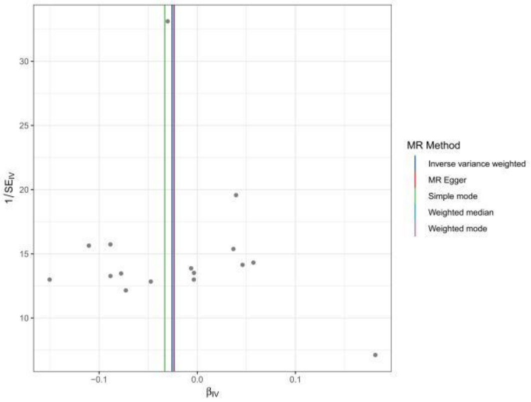

Forest plot

Funnel plot

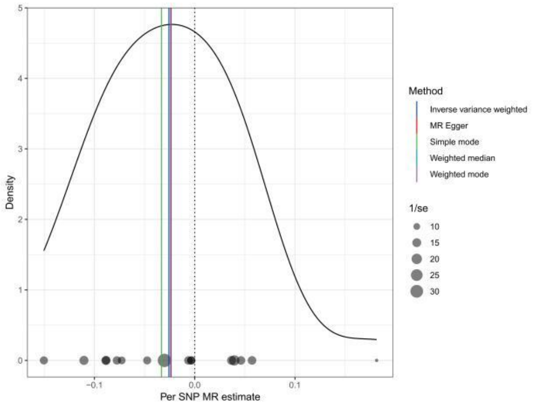

Density plot
