# Supplementary material for: Magnesium depletion score and erectile dysfunction: A cross-sectional and Mendelian randomization study
Source: Medicine (Baltimore). 2026 Jul 24;105(30):e49938. doi: 10.1097/MD.0000000000049938 (PMC13406066; doi:10.1097/MD.0000000000049938)
Supplement: Supplementary file 9 [file medi-105-e49938-s009.docx]

Table S3. Association between the magnesium depletion score (binary variable) and erectile dysfunction.

| Model | Variable | Category | OR (95% CI) | P value |
| --- | --- | --- | --- | --- |
| Model 1 | MDS | < 2 | Reference |  |
|  |  | ≥ 2 | 8.46 (6.95, 10.29) | < 0.001 |
| Model 2 | MDS | < 2 | Reference |  |
|  |  | ≥ 2 | 2.50 (1.90, 3.28) | < 0.001 |
|  | Age |  | 1.09 (1.08, 1.10) | < 0.001 |
|  | Race | Mexican American | Reference |  |
|  |  | Non-Hispanic Black | 0.69 (0.50, 0.96) | 0.040 |
|  |  | Non-Hispanic White | 0.71 (0.49, 1.01) | 0.075 |
|  |  | Other Hispanic | 1.19 (0.62, 2.30) | 0.612 |
|  |  | Other Race | 0.88 (0.39, 1.97) | 0.755 |
|  | Education | Less than 9th grade | Reference |  |
|  |  | 9–11th grade | 1.01 (0.60, 1.69) | 0.981 |
|  |  | High school graduate | 0.51 (0.32, 0.83) | 0.014 |
|  |  | Some college or AA degree | 0.53 (0.32, 0.88) | 0.023 |
|  |  | College graduate or above | 0.53 (0.34, 0.81) | 0.009 |
|  | PIR |  | 0.86 (0.80, 0.91) | < 0.001 |
| Model 3 | MDS | < 2 | Reference |  |
|  |  | ≥ 2 | 2.27 (1.74, 2.98) | < 0.001 |
|  | Age |  | 1.08 (1.07, 1.09) | < 0.001 |
|  | Race | Mexican American | Reference |  |
|  |  | Non-Hispanic Black | 0.69 (0.50, 0.95) | 0.039 |
|  |  | Non-Hispanic White | 0.68 (0.47, 0.96) | 0.049 |
|  |  | Other Hispanic | 1.25 (0.64, 2.42) | 0.529 |
|  |  | Other Race | 0.89 (0.42, 1.89) | 0.762 |
|  | Education | Less than 9th grade | Reference |  |
|  |  | 9–11th grade | 1.04 (0.62, 1.76) | 0.881 |
|  |  | High school graduate | 0.54 (0.33, 0.87) | 0.027 |
|  |  | Some college or AA degree | 0.57 (0.34, 0.93) | 0.044 |
|  |  | College graduate or above | 0.65 (0.42, 1.01) | 0.077 |
|  | PIR |  | 0.87 (0.81, 0.93) | 0.002 |
|  | WC |  | 1.02 (1.01, 1.03) | 0.003 |
|  | Smoking | No | Reference |  |
|  |  | Yes | 1.32 (1.09, 1.61) | 0.016 |
|  | Moderate activity | No | Reference |  |
|  |  | Yes | 0.89 (0.71, 1.11) | 0.332 |
|  | Vigorous activity | No | Reference |  |
|  |  | Yes | 0.69 (0.52, 0.93) | 0.028 |
|  | Total energy intake |  | 1.00 (1.00, 1.00) | 0.059 |
|  | Dietary fiber intake |  | 1.00 (0.99, 1.02) | 0.407 |
| Model 4 | MDS | < 2 | Reference |  |
|  |  | ≥ 2 | 2.17 (1.69, 2.78) | < 0.001 |
|  | Age |  | 1.08 (1.07, 1.09) | < 0.001 |
|  | Race | Mexican American | Reference |  |
|  |  | Non-Hispanic Black | 0.63 (0.44, 0.90) | 0.038 |
|  |  | Non-Hispanic White | 0.68 (0.47, 0.98) | 0.076 |
|  |  | Other Hispanic | 1.11 (0.55, 2.23) | 0.784 |
|  |  | Other Race | 0.77 (0.35, 1.70) | 0.540 |
|  | Education | Less than 9th grade | Reference |  |
|  |  | 9–11th grade | 1.02 (0.59, 1.79) | 0.934 |
|  |  | High school graduate | 0.55 (0.33, 0.90) | 0.050 |
|  |  | Some college or AA degree | 0.54 (0.33, 0.91) | 0.054 |
|  |  | College graduate or above | 0.64 (0.41, 1.01) | 0.098 |
|  | PIR |  | 0.87 (0.81, 0.94) | 0.009 |
|  | WC |  | 1.01 (1.00, 1.02) | 0.035 |
|  | Smoking | No | Reference |  |
|  |  | Yes | 1.30 (1.06, 1.60) | 0.037 |
|  | Moderate activity | No | Reference |  |
|  |  | Yes | 0.91 (0.72, 1.13) | 0.421 |
|  | Vigorous activity | No | Reference |  |
|  |  | Yes | 0.73 (0.55, 0.97) | 0.065 |
|  | Total energy intake |  | 1.00 (1.00, 1.00) | 0.133 |
|  | Dietary fiber intake |  | 1.00 (0.99, 1.01) | 0.614 |
|  | HDL-C |  | 1.00 (1.00, 1.01) | 0.440 |
|  | TG |  | 1.00 (1.00, 1.00) | 0.739 |
|  | Hypertension | No | Reference |  |
|  |  | Yes | 1.04 (0.83, 1.29) | 0.759 |
|  | Diabetes | No | Reference |  |
|  |  | Yes | 2.19 (1.58, 3.04) | 0.002 |
|  | CRP |  | 1.03 (0.93, 1.14) | 0.587 |
|  | Mental health status | No | Reference |  |
|  |  | Yes | 1.91 (1.31, 2.77) | 0.012 |
| Model 5 | MDS | < 2 | Reference |  |
|  |  | ≥ 2 | 2.06 (1.60, 2.65) | 0.002 |
|  | Age |  | 1.08 (1.07, 1.09) | < 0.001 |
|  | Race | Mexican American | Reference |  |
|  |  | Non-Hispanic Black | 0.63 (0.44, 0.89) | 0.050 |
|  |  | Non-Hispanic White | 0.66 (0.46, 0.95) | 0.077 |
|  |  | Other Hispanic | 1.11 (0.56, 2.19) | 0.784 |
|  |  | Other Race | 0.77 (0.35, 1.71) | 0.555 |
|  | Education | Less than 9th grade | Reference |  |
|  |  | 9–11th grade | 1.03 (0.59, 1.81) | 0.916 |
|  |  | High school graduate | 0.56 (0.35, 0.91) | 0.067 |
|  |  | Some college or AA degree | 0.56 (0.33, 0.94) | 0.079 |
|  |  | College graduate or above | 0.67 (0.43, 1.07) | 0.154 |
|  | PIR |  | 0.87 (0.80, 0.94) | 0.016 |
|  | WC |  | 1.01 (1.00, 1.02) | 0.045 |
|  | Smoking | No | Reference |  |
|  |  | Yes | 1.28 (1.04, 1.58) | 0.068 |
|  | Moderate activity | No | Reference |  |
|  |  | Yes | 0.89 (0.72, 1.11) | 0.364 |
|  | Vigorous activity | No | Reference |  |
|  |  | Yes | 0.73 (0.55, 0.96) | 0.077 |
|  | Total energy intake |  | 1.00 (1.00, 1.00) | 0.166 |
|  | Dietary fiber intake |  | 1.00 (0.99, 1.01) | 0.606 |
|  | HDL-C |  | 1.00 (1.00, 1.01) | 0.355 |
|  | TG |  | 1.00 (1.00, 1.00) | 0.742 |
|  | Hypertension | No | Reference |  |
|  |  | Yes | 1.02 (0.82, 1.27) | 0.850 |
|  | Diabetes | No | Reference |  |
|  |  | Yes | 2.16 (1.56, 3.01) | 0.006 |
|  | CRP |  | 1.03 (0.93, 1.14) | 0.586 |
|  | Mental health status | No | Reference |  |
|  |  | Yes | 1.85 (1.27, 2.69) | 0.024 |
|  | CHF | No | Reference |  |
|  |  | Yes | 1.29 (0.78, 2.15) | 0.369 |
|  | CHD | No | Reference |  |
|  |  | Yes | 1.58 (1.09, 2.29) | 0.059 |

Note: Values are ORs with 95% CIs from survey-weighted logistic regression models. Model 1 was unadjusted. Model 2 adjusted for age, race, education, and PIR. Model 3 further adjusted for WC, smoking, moderate activity, vigorous activity, total energy intake, and dietary fiber intake. Model 4 further adjusted for HDL-C, TG, hypertension, diabetes, CRP, and mental health status. Model 5 additionally adjusted for CHF and CHD. Reference indicates the reference category. Abbreviations: MDS, magnesium depletion score; ED, erectile dysfunction; OR, odds ratio; CI, confidence interval; PIR, poverty-to-income ratio; WC, waist circumference; HDL-C, high-density lipoprotein cholesterol; TG, triglycerides; CRP, C-reactive protein; CHF, congestive heart failure; CHD, coronary heart disease.
